# Pseudotetraivprolides from *Pseudomonas entomophila* Provide Insights into the Biosynthesis of Detoxin/Rimosamide‐Like Anti‐Antibiotics

**DOI:** 10.1002/anie.202513287

**Published:** 2025-12-12

**Authors:** Edna Bode, Julia Büllesbach, Kevin Bauer, Yan‐Ni Shi, Simon Reiners, Ziheng Cui, Petra Happel, Yi‐Ming Shi, Kevin Hoffmann, Peter Grün, Bianca Pommerenke, Uli Kazmaier, Martin Grininger, Helge B. Bode

**Affiliations:** ^1^ Department of Natural Products in Organismic Interactions Max Planck Institute for Terrestrial Microbiology Marburg 35043 Germany; ^2^ Molecular Biotechnology Department of Biosciences Goethe University Frankfurt Frankfurt am Main 60438 Germany; ^3^ Institute for Organic Chemistry Saarland University Saarbrücken 66123 Germany; ^4^ Buchmann Institute for Molecular Life Sciences Institute of Organic Chemistry and Chemical Biology Goethe University Frankfurt Frankfurt am Main 60438 Germany; ^5^ Department of Biochemistry & Synthetic Metabolism Max Planck Institute for Terrestrial Microbiology Marburg 35043 Germany; ^6^ Center for Synthetic Microbiology (SYNMIKRO) Phillips University Marburg Marburg 35043 Germany; ^7^ Department of Chemistry Phillips University Marburg Marburg 35043 Germany; ^8^ LOEWE Priority Program Tree‐M Phillips University Marburg Marburg 35043 Germany; ^9^ Present address: Institute of Chemical Biology, Shenzhen Bay Laboratory Shenzhen 518132 China; ^10^ Present address: Institute of Synthetic Biology (iSynBio) Shenzhen Institutes of Advanced Technology (SIAT) Chinese Academy of Sciences Shenzhen 518055 China

**Keywords:** Acetyl transferase protein complex, Natural product, NRPS/PKS hybrid biosynthesis pathway, Thioesterase, Trans‐AT polyketide synthase

## Abstract

Novel variants of known natural product (NP) classes can provide valuable insights into their biosynthesis, mechanisms of action, and potential as drug leads across the entire class. Here, we describe a novel member of the widespread detoxine/rimosamide‐like (DRL) natural products, named pseudotetraivprolide, produced by *Pseudomonas* strains. Pseudotetraivprolide exhibits the characteristic DRL‐activity of protecting *Bacillus cereus* against the antibiotic blasticidin S. Through the generation of multiple deletion and complementation mutants, heterologous expression experiments, identification and structure elucidation of several derivatives, chemical synthesis of main derivatives, enzymatic characterization of individual biochemical steps, and detailed homology modelling of enzyme complexes, we elucidated key aspects of its biosynthesis. Our findings demonstrate that the primary metabolism‐derived malonyl CoA:ACP transacylase (FabD) functions as a *trans*‐AT in the biosynthesis pathway. Furthermore, we suggest an order for all late‐stage modifications and assign a function for the three conserved hypothetical proteins PipDFG acting as last‐step acetylation complex responsible for stabilization and activation of the final product.

## Graphical abstract

Pseudotetraivprolide is a new player among the Detoxin/Rimosamide‐like natural products! Its biosynthesis requires FabD from primary metabolism and a PipDFG complex for final‐step acetylation – unlocking its anti‐antibotic activity.

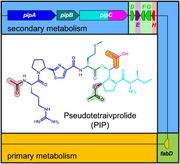

## Introduction

Bacteria carry biosynthesis gene clusters (BGCs) responsible for the production of bioactive natural products (NP). While we are able to speed up identification of NPs and their BGCs based on progress in mass spectrometry and bioinformatic tools like antiSMASH,^[^ ^1^, ^2^ ^]^ we often do not understand well, how and for which ecological function they were evolved. That they were developed for specific functions is very likely, since related BGCs producing structurally similar NPs often share a set of core genes but differ in the presence of accessory genes involved in modifying the core structure. Representative examples include the biosynthetic pathways of glycopeptide antibiotics,^[^ ^3^ ^]^ pyrrolizidine alkaloids^[^ ^4^ ^]^ or detoxin/rimosamide^[^ ^5^ ^]^ NP families among others. In the context of drug discovery and development, the identification of such novel NP derivatives is particularly valuable, as it increases the chance of either discovering suitable drugs directly or enables structural diversification by the use of accessory genes or their corresponding enzymes for compound optimization.

Furthermore, although we are able to rather quickly identify promising BGCs of interest involved in the biosynthesis of desired NP families from thousands of available genomes using a set of great tools,^[^ ^6^, ^7^, ^8^, ^9^, ^10^, ^11^ ^]^ we still have to access both the BGC and the NP to confirm their structure, biosynthesis, and function. Several strategies have been successfully applied for this purpose, including heterologous expression of complete BGCs using different methods,^[^ ^12^, ^13^, ^14^, ^15^ ^]^ activation of BGC expression by varying culture conditions^[^ ^16^ ^]^ or employing modern elicitor‐based approaches,^[^ ^17^, ^18^ ^]^ or BGC activation using targeted promoter exchange approaches to induce expression of silent gene clusters.^[^ ^19^, ^20^, ^21^ ^]^

In our effort to identify novel NPs from entomopathogenic bacteria, we discovered a BGC in *Pseudomonas entomophila* L48 showing high similarity to BGCs of the detoxin/rimosamide family. Detoxin/rimosamide‐like (DRL) NPs are named after the first described members of this structurally diverse NP class.^[^ ^22^, ^23^ ^]^ They are produced by hybrid non‐ribosomal peptide synthetases (NRPS) and polyketide synthases (PKS) found in a variety of Gram‐positive *Streptomyces* ^[^ ^5^, ^23^, ^24^ ^]^ and Gram‐negative genera such as *Pseudomonas*, *Chitinimonas* ^[^ ^25^, ^26^ ^]^ and *Pseudovibrio*.^[^ ^27^ ^]^ DRL compounds have been described as anti‐antibiotics, capable of protecting *Bacillus cereus* against the nucleoside antibiotic blasticidin S,^[^ ^22^, ^28^ ^]^ most likely by blocking peptide ABC‐importers or oligopeptide transporters.^[^ ^29^, ^30^ ^]^

We activated the corresponding BGC using the easyPACId promoter exchange approach,^[^ ^20^ ^]^ which enabled the analysis of the biosynthesis and structural characterization of the produced NPs. In this study, we show the structure of all main derivatives, the chemical synthesis of two main compounds and the detailed biosynthetic pathway of a new DRL from *P*. *entomophila*, named pseudotetraivprolide. We elucidated several unusual yet conserved features of DRL biosynthesis, including novel insights into the activity of the *trans*‐AT PKS module requiring the primary metabolism enzyme malonyl CoA:ACP transacylase FabD. Furthermore, we characterized the function and interaction of three conserved hypothetical proteins, which mediate late‐stage acetylation and stabilization of the final product.

## Results and Discussion

### Identification of a New Type of Detoxin/Rimosamide BGC

*P*. *entomophila* L48^[^ ^31^ ^]^ is the producer of several known NPs: The lipodepsipeptide entolysin,^[^ ^32^ ^]^ the iron siderophores pseudomonine^[^ ^33^ ^]^ and pyoverdin,^[^ ^33^ ^]^ pyreudione^[^ ^34^ ^]^ and the pseudomonas virulence factor (pvf)^[^ ^35^ ^]^ are all derived from NRPS‐encoding BGCs, while the bioactive oxazole‐containing labradorins^[^ ^36^ ^]^ are produced NRPS‐independent (Figure S1.1a).

**Figure 1:**
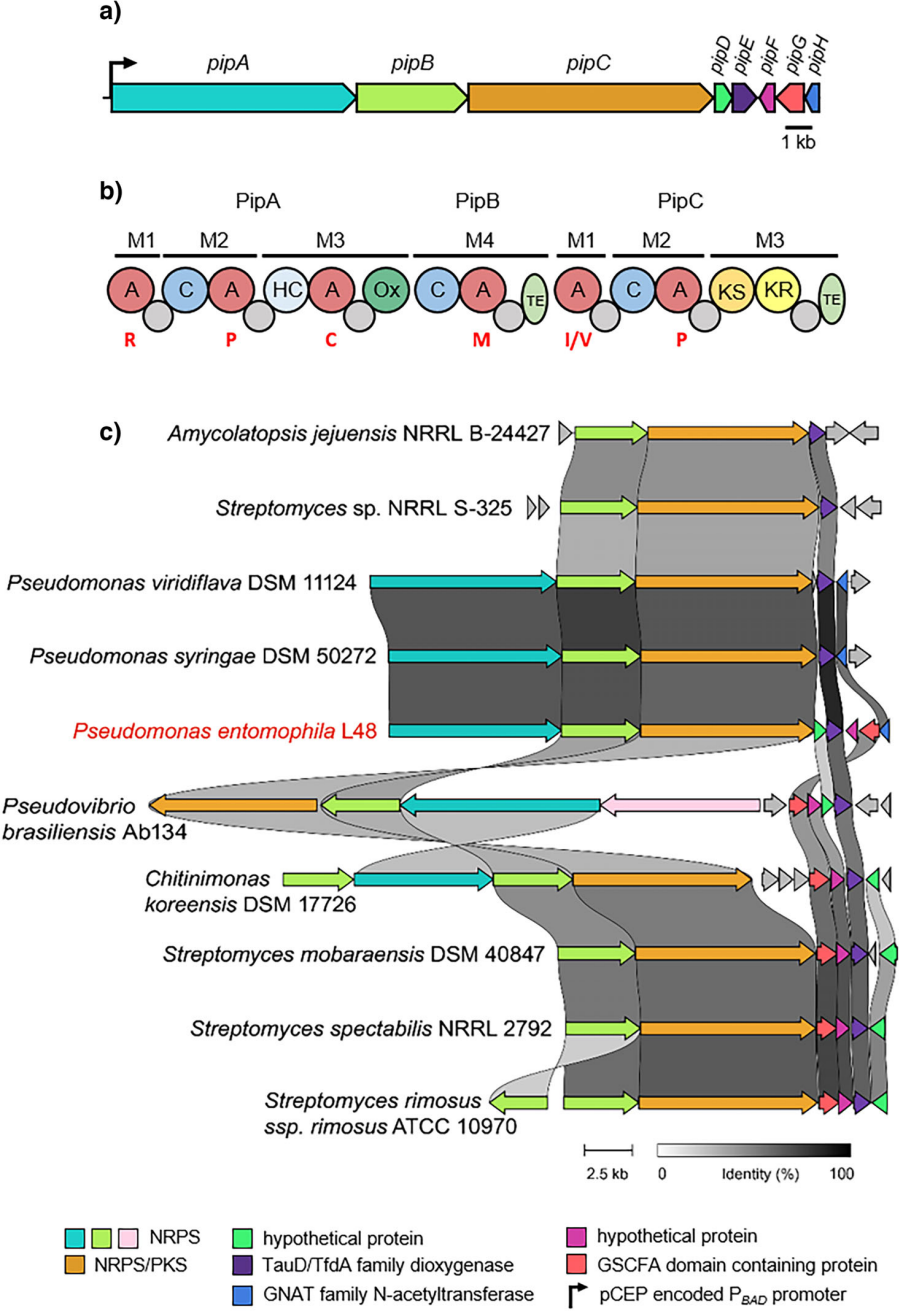
Biosynthetic gene cluster of pseudotetraivprolide **a**) domain organization of the NRPSs PipA and PipB and the NRPS/PKS hybrid PipC **b**). Predicted domains: Adenylation (A), with substrate specificities indicated by one‐letter amino acid codes (red); Thiolation (grey); Condensation (blue); Heterocyclisation (HC) (light blue); Oxidation (green); Thioesterase (TE) (olive); Ketosynthase (KS) (orange); and Ketoreductase domain (KR) (yellow). Alignment of DRL BGCs with the pseudotetraivprolide BGC of *P*. *entomophila* L48 (red) based on CAGECAT analysis **c**).

Besides these known metabolites, a BGC with similarity to the DRL family is present in the genome, representing a putatively new member of this widespread NP family. It is composed of eight genes (PSEEN_RS12600‐RS12635) *pipA*‐*H* (Figure 1a, Table S1.1).

The two NRPS PipAB are involved in producing a tetrapeptide.^[^ ^19^ ^]^ The signature NRPS/PKS hybrid PipC, characteristic for all DRL BGCs, is responsible for the elongated dipeptide unit (Figure 1b). Distribution analysis using the CompArative GEne Cluster Analysis Toolbox CAGECAT^[^ ^37^ ^]^ revealed that the *pipA‐H* BGC is highly abundant among *Pseudomonas* species (Figure 1c). A characteristic feature for the *pip* BGC within *Pseudomonadaceae* is the combination of two NRPS (PipAB) and one PKS/NRPS (PipC). In addition to the highly conserved dioxygenase PipE, present in all DRL BGCs, the *pip* BGC encodes four additional genes: a putative N‐acetyltransferase (PipH), a GSCFA‐domain‐containing protein (PipG), a member of the SGNH/GDSL hydrolase family (named for its conserved N‐terminal motif but with still unknown enzymatic activity), and two hypothetical proteins, PipD and PipF, of unknown function. Notably PipD, PipF and PipG always occur together and are exclusively associated with the respective DRL BGC. Whereas *pipG* and *pipF* are always adjacent, localization and orientation of *pipD* may vary (Figure 1c). Interestingly, the *pipH* gene is found only in *P*. *entomophila, P*. *viridiflava*, *P*. *syringae* and other *Pseudomonas* strains, that share the same NRPS NRPS/PKS gene composition and lack a C_starter_ condensation domain in PipA.

### Activation of the *Pip* BGC

**Figure 2:**
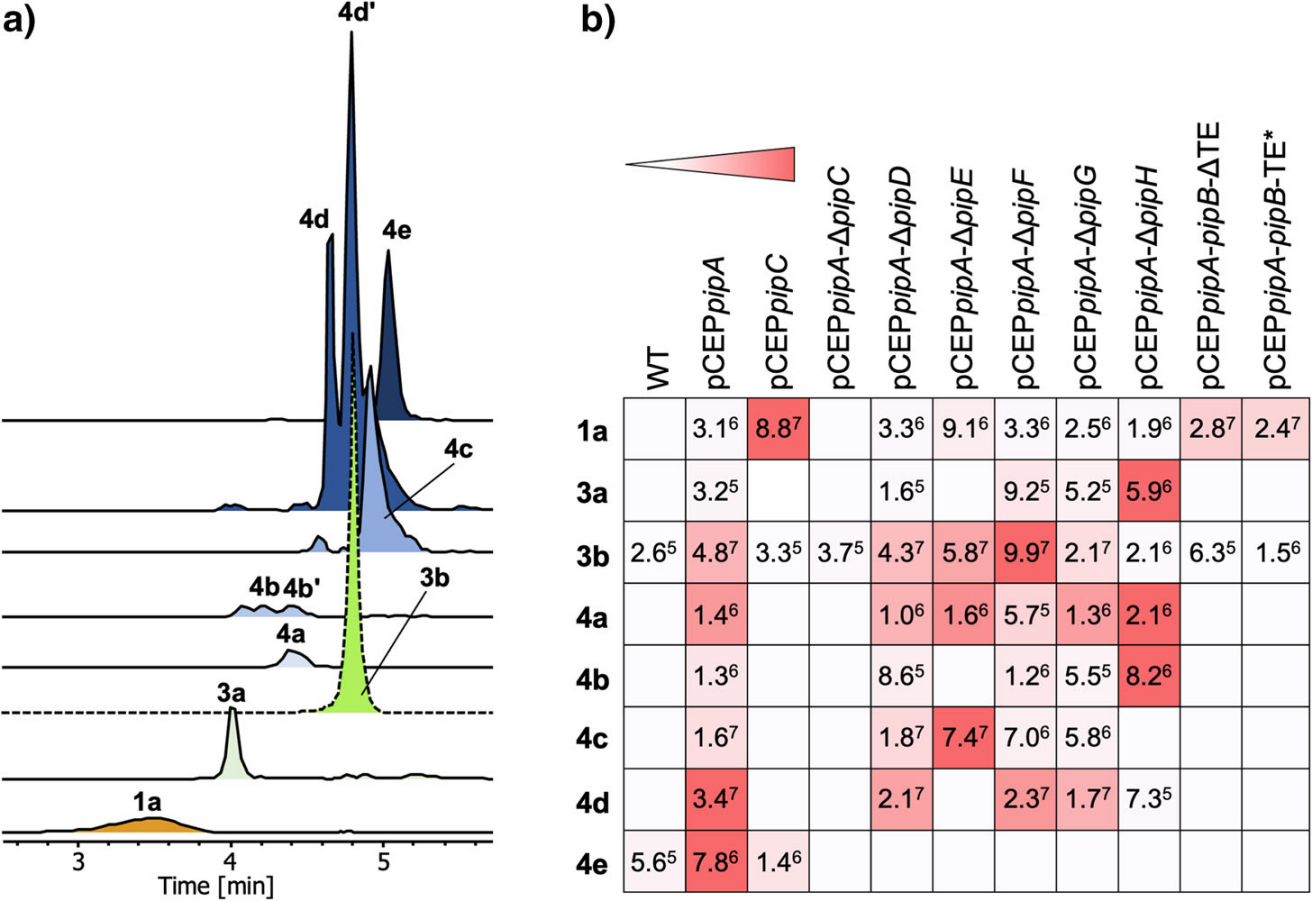
EICs of pseudotetraivprolide derivatives detected in culture extracts of the induced pCEP*pipA* mutant, dashed line indicates a 10‐fold reduced signal **a**). Heat map showing the production of pseudotetraivprolide derivatives comparing selected mutants with dark red indicating highest production to white indicating no production. Signal intensities from the HPLC/MS analysis are abbreviated as 3.1^6^ for 3.1 x 10^6^ **b)**.

**Figure 3:**
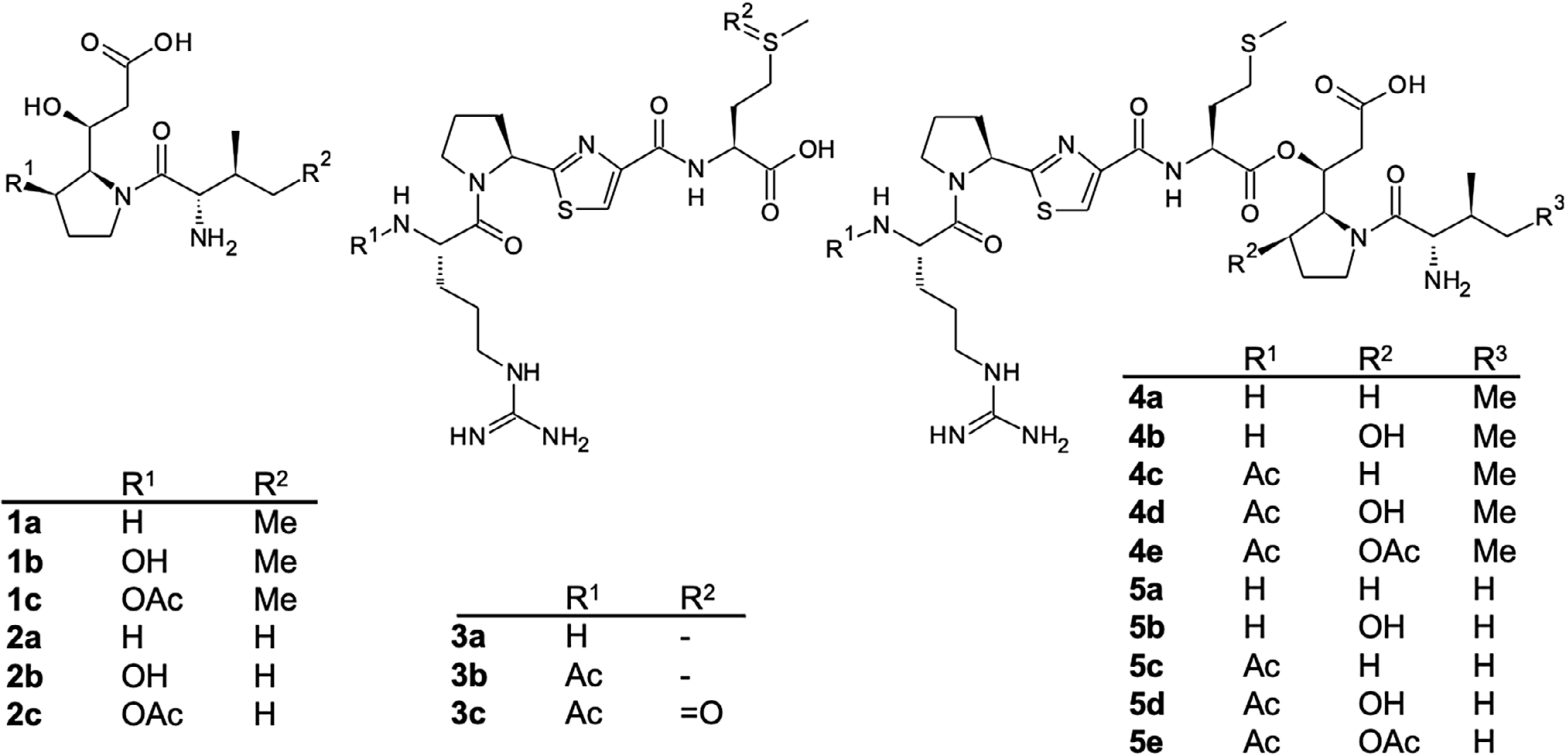
Overview of identified ivprolides (**1** & **2**), pseudotetratides (**3**) and pseudotetraivprolides (**4** & **5**) derivatives.

Activation of the *pip* BGC in *P*. *entomophila* via the easyPACId approach^[^ ^19^, ^20^ ^]^ led to the production of a wide range of derivatives (Figures 2 and 3; Figures S2.1–S2.15 and Table S2.1). In addition to the previously described pseudotetratide (**3b**),^[^ ^19^ ^]^ **3a**‐**3c** were identified as new derivatives. **1a**‐**1c**, named ivprolides, and the full‐length product pseudotetraivprolide (PIP, **4a**‐**4e**) were identified (Figures 2 and 3), while only minute amounts of **3b** and **4e** were detected in the wild‐type strain (Figure 2b, Figures S2.4 and S2.10). Products **3** and **4** differ at the N‐terminus, which may be acetylated. Moreover, the C‐terminal proline ring in both variants might be hydroxylated and acetylated. The structures of all derivatives were elucidated by detailed MS‐MS analysis (Figures S2.1–S2.15) as well as NMR analysis for **1a** (Figures S2.17–S2.21), **4c** (Figures S2.22–S2.28 and Table S2.1) and **4e** (Figures S2.29–S2.32 and Table S2.2; all in *Supporting Information‐2: Compound identification and structure elucidation*). In addition to the main compounds containing isoleucine, corresponding valine‐containing ivprolide (**2a**) and pseudotetraivprolide (**5a**‐**5e**) were found as minor derivatives (Figures S2.1, S2.11–S2.15).

For the full‐length PIP derivatives **4b** and **4d,** which carry a hydroxyl group at the C‐terminal proline, two isobaric peaks were consistently observed (Figures 2, S2.7, S2.9, S2.12 and S2.14), suggesting two distinct ester variants most likely formed autocatalytically (Figure S1.2). Furthermore, methionine sulfoxide derivatives **3c**, **4f**, **4g**, **5f**, and **5g** were also identified under certain experimental conditions (Figures S2.5, S2.7, S2.9, S2.12 and S2.14).

For functional analysis of PIP, we constructed a *pipA* promoter exchange mutant in a low‐background wild‐type strain, Δ*PELP4*, which lacks BGCs responsible for pseudomonine, entolysine, labradorin, pyoverdin and an additional unknown BGC4 (Figure S1.1a). Extracts from this strain, Δ*PELP4*‐pCEP*pipA*, protect *Bacillus cereus* against the antibiotic blasticidin S as previously described for several DRL derivatives^[^ ^22^ ^]^ (Figure S1.1b,c). When testing the pure compounds **3b**, **4c**, **4e**, and **5e**, only the full‐length products but not pseudotetratide **3b** conferred protection against blasticidin S. Interestingly, the presence of the *O*‐acetyl moiety in **4e** and **5e** seems to enhance activity, since **4c** showed reduced protective effects (Figure S1.3). In contrast to pseudovibramide^[^ ^27^ ^]^ and chitinimide,^[^ ^26^ ^]^ no difference in swarming behavior was observed for any of the mutants generated compared to the wild‐type (WT) strain or PIP‐producing mutants (data not shown), probably because *P. entomophila* relies on entolysin for this function.^[^ ^32^ ^]^

### Chemical Synthesis of Pseudotetraivprolides

**Figure 4:**
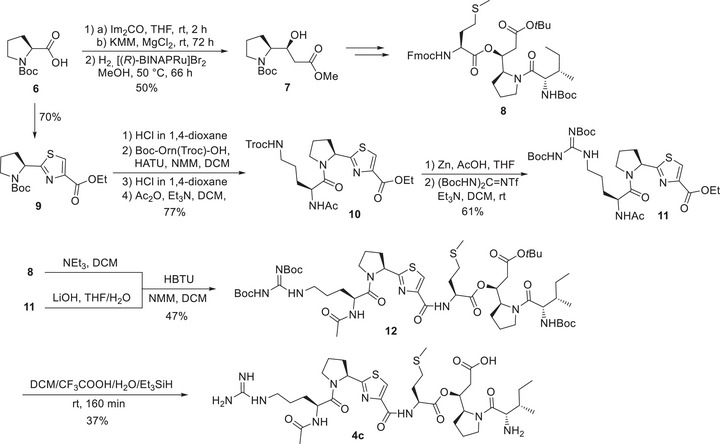
Synthesis of pseudotetraivprolide **4c**. Im (imidazolyl), KMM (potassium methyl malonate), BINAP (2,2′‐bis(diphenylphosphino)‐1,1′‐binaphthyl), HATU (1‐[Bis(dimethylamino)methylene]‐1H‐1,2,3‐triazolo[4,5‐b]pyridinium 3‐oxide hexafluorophos‐phate), HBTU (3‐[Bis(dimethylamino)methyliumyl]‐3H‐benzotriazol‐1‐oxide hexafluorophos‐phate).

To confirm the overall structure of the full‐length derivatives, including the stereochemistry of all incorporated amino acids, the main product **4c** was synthesized (see *Supporting Information* *3* *: Chemical Synthesis* for details), which unambiguously confirmed the structure of the natural product. Briefly, starting from Boc‐protected proline **6** the β‐hydroxyester **7** (Figure 4) was prepared according to Greck et al.^[^ ^38^ ^]^ 1,1′‐Carbonyldiimidazole (CDI) activation and coupling with potassium methyl malonate (KMM) provided the corresponding β‐ketoester which was subjected to an asymmetric Noyori hydrogenation.^[^ ^39^ ^]^ Subsequent standard protecting group manipulations and peptide couplings provided the right‐hand side tripeptide **8**. For the left part of the molecule, **6** was converted into thiazole fragment **9** following a procedure from Deng and Taunton's synthesis of ceratospongamide.^[^ ^40^ ^]^ After Boc‐deprotection, the thiazole building block **9** was coupled with Boc‐Orn(Troc)‐OH via HATU‐mediated peptide coupling with 83% yield. Due to significant epimerization at the α‐position observed in direct couplings with acetylated ornithine and arginine,^[^ ^41^, ^42^ ^]^ a two‐step protocol was employed. After removal of the Boc‐carbamate, acetylation with acetic anhydride gave the desired dipeptide **10** in 94% yield. In the last step, the Troc‐carbamate was removed to introduce the Di‐Boc‐guanidine unit.^[^ ^43^ ^]^ Deprotection of the two peptide fragments **8** and **11**, followed by coupling using HBTU, provided protected pseudotetraivprolide **4c** in acceptable yield. In the final step, global deprotection was achieved using a cleavage cocktail containing Et_3_SiH as a scavenger to suppress side reactions induced by the *tert‐*butyl cations.^[^ ^44^ ^]^ Pseudotetraivprolide **5c** was obtained analogously.

### Functional Analysis of *Pip* Deletions

To elucidate the function of all Pip enzymes, the corresponding genes were deleted in‐frame in order to avoid polar effects on downstream genes, followed by a detailed HPLC/MS analysis of the resulting derivatives (Figure 2b and Figure S1.4). All deletions were analyzed under the control of the P_BAD_ promoter.

In extracts of Δ*pipC* mutant, only traces of the tetrapeptide **3a** and **3b** were detected (Figures 2, S2.3 and S2.4), indicating that their release from the enzyme depends on the presence of PipC. Promoter exchange upstream of *pipC* resulted in overproduction of **1a** and **2a** (Figure S2.1). Minimal amounts of **3b** and **4e** were also produced (Figure 2b, Figures S2.4 and S2.10) due to the activity of the native *pipA* promoter. In the Δ*pipE* mutant an accumulation of all non‐hydroxylated compounds **3b**, **4a** and **4c** was observed (Figures 2, S2.4, S2.6 and S2.8). Deletion of *pipD*, *pipF* or *pipG* led to accumulation of all non‐*O*‐acetylated precursors with **3b** and **4d** as the main products (Figures 2, S2.4 and S2.9). Upon deletion of the *N*‐acetyltransferase gene *pipH*, only compounds lacking an *N*‐terminal acetylation were produced, primarily **3a**, along with **4a** and **4b** (Figures 2, S2.3, S2.6 and S2.7). Traces of **3b** and **4d** detected in this mutant suggest the presence of another acetyltransferase, encoded elsewhere in the genome, that catalyzes this reaction. All deletions were successfully complemented with plasmid‐encoded copies of the respective genes (Figure S1.5) except for *pipG*. However, *pipG* deletion could be complemented by *pipFG* and *pipFGH* (Figure S1.6), indicating that PipF and PipG likely form a catalytic complex for the final acetylation step leading to **4e**. Deletion of all accessory genes *pipDEFGH* resulted in the accumulation of **3a** and **4a** in the corresponding mutant. Complementation of this strain with plasmid‐encoded *pipDEFGH* led to strong overproduction of **4e** (Figure S1.7.j), enabling its preparative isolation and leading to the discovery of new derivatives **4h** and **5h**, each carrying an additional acetyl group at the amine group of Ile or Val, respectively (Figure S2.16). Furthermore, complementation of Δ*pipDEFGH* with plasmid‐encoded *pipDEFG* led to the production of **4i** (Figure S1.7.l), which lacks the N‐terminal acetyl group. Here, additional hydrolysis products of **4i** – namely fully substituted **1c** and **3a** and the cyclic variant **4j** – were detected, although only in trace amounts (Figure S1.8).

### Core Structure Biosynthesis

Both PipB and PipC contain a thioesterase (TE) domain, and it was postulated early on that the PipB‐TE catalyzes ester bond formation.^[^ ^23^ ^]^ Indeed, this was recently shown for the related compound chitinimide through detailed in vivo and in vitro studies.^[^ ^26^ ^]^ Similarly, deletion of the TE domain in PipB or substitution of the essential residue Ser‐2856 with Ala completely abolished production of all full‐length products while resulting in accumulation of **1a** and **3b** (Figure 2b and Figure S1.9), indicating that **1a** can be released from the PipC‐TE by hydrolysis. However, since neither hydroxylated nor *O*‐acetylated products (**1b** and **1c**) can be detected in the *pipB* TE mutants or upon insertion of the inducible promoter upstream of *pipC* (Figure 2b), we postulate that hydroxylation and acetylation predominantly takes place on the full‐length products **4**. This hypothesis was further supported by the heterologous expression of *pipC* or *pipCDE* in *E. coli*, which resulted exclusively in the production of **1a** (Figure S1.10). This biosynthetic timing differs from that observed in the pseudovibramide^[^ ^27^ ^]^ and chitinimide^[^ ^26^ ^]^ biosynthesis, where analogs of **1c** were detected even when parts of the NRPS homologous to PipA were deleted. In *P*. *entomophila*, we attribute the presence of **1b** and **1c** in certain mutants to hydrolytic cleavage of the fully modified, full‐length products **4b**, **4d** and **4e**. Supporting this, we observed increased amounts of these products and methyl esters of **3b** when MeOH was used for compound extraction (data not shown), as also reported for chitinimide.^[^ ^26^ ^]^

**Figure 5:**
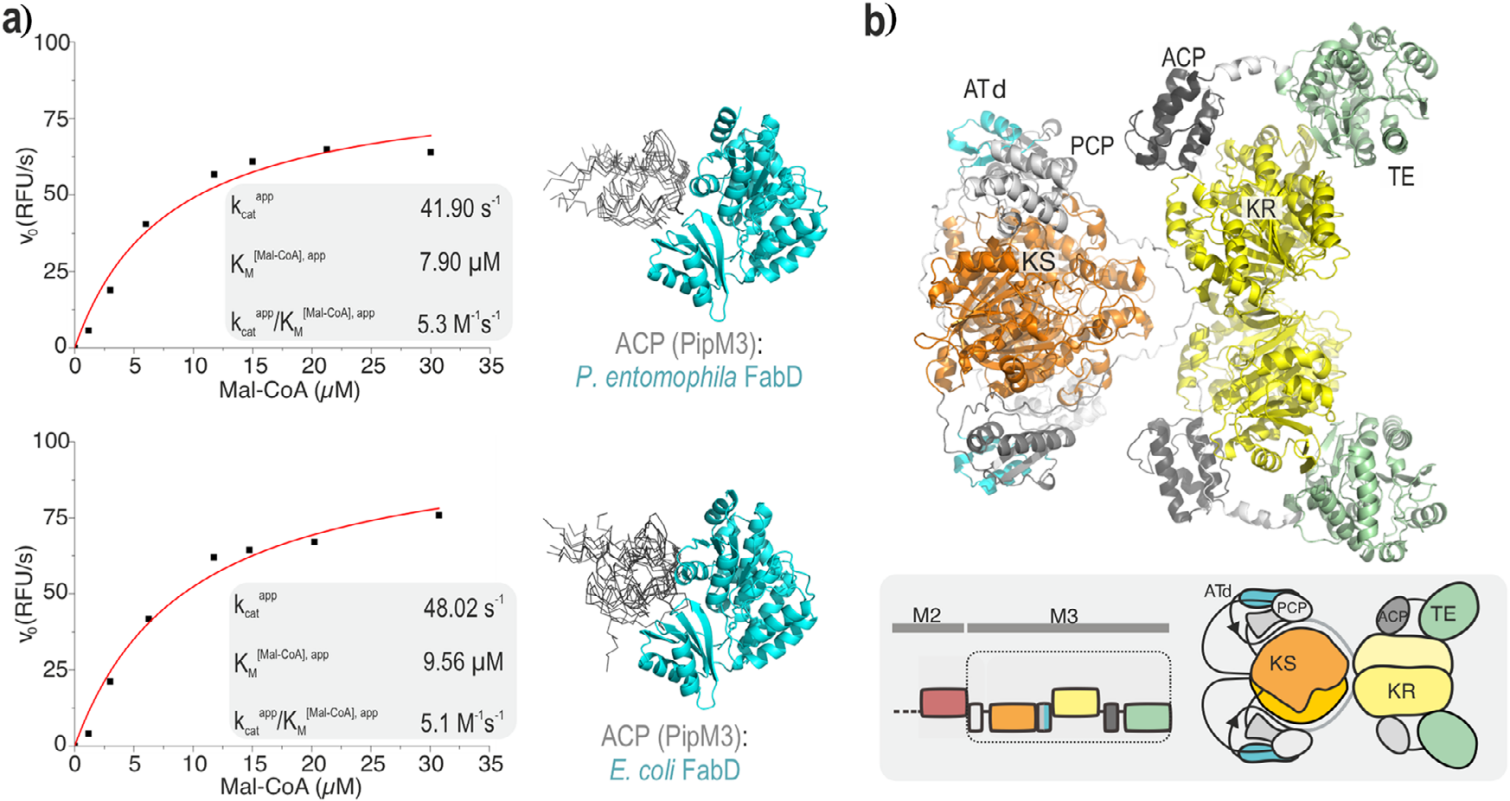
ACP‐FabD interaction and structural modeling of PipC/Module‐3. **a**) Michaelis‐Menten fits of transacylation titration curves of *P. entomophila* FabD (top panel) and *E. coli* FabD (bottom panel) with malonyl‐CoA and ACP in fixed concentration, respectively. Measurements were performed on one biological sample in technical replication. Shown are the two predicted complexes, each represented by five structural models as ranked by AlphaFold based on internal confidence scores (ACP in grey in ribbon representation, FabDs in cyan). The higher positional variability of *P. entomophila* ACP in complex with *E. coli* FabD indicates uncertainty of the prediction. **b**) PipC/Module‐3, including the upstream PCP, modeled by AlphaFold.^[^ ^51^ ^]^ The N‐terminal PCP is positioned at the KS domain, while the internal ACP interacts with the KR domain.^[^ ^52^, ^53^ ^]^ Although PKS TE domains are typically dimeric, the TE domain is modeled here in its monomeric form interacting with the KR. The inset shows PipC/Module‐3 in domain architecture with the upstream C‐domain not included in the AlphaFold model in orange. Domain colors as introduced in Figure 1.

Interestingly, heterologous production of **1a** from *pipC* in *E. coli* did not require an acyltransferase (AT), despite the absence of an AT domain in all PipC homologs from known DRL‐type BGCs. Structural modeling of PipC confirmed the presence of an AT docking domain (ATd) and absence of a canonical AT domain (Figure 5 and Figures S1.11–S1.14) indicating that the Pip pathway, like other DRL pathways, is indeed of the *trans*‐AT PKS/NRPS type. The pip BGC and the entire *P. entomophila* genome lack any typical *trans*‐AT enzymes, as confirmed by BLAST analysis. Therefore, the primary metabolic enzyme malonyl‐CoA:ACP‐transacylase FabD (PSEEN_RS07520) likely acts as AT for PipC. Enzyme kinetic analysis of the transacylation reaction confirmed that FabD from both *E. coli* and *P. entomophila* catalyzes the AT‐mediated transacylation of the PipC acyl carrier protein (ACP) (Figure 5a).^[^ ^45^, ^46^ ^]^ Self‐acylation of PipC‐ACP was observed at concentrations ≥25 µM (Figure S1.10d). For determination of kinetic parameters, initial velocities of FabD‐mediated transacylation were corrected for background self‐acylation rates.

### Biosynthesis of Pseudotetraivprolide

**Figure 6:**
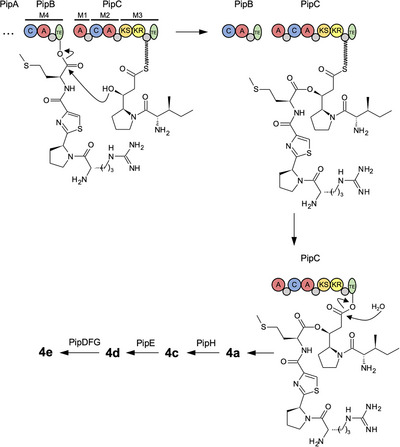
Proposed timing of the late‐stage biosynthesis of pseudotetraivprolide focusing on the TEs from PipB and PipC, as well as PipDEFGH. For domain explanations of PipB and PipC see Figure 1.

Pseudotetratide **3a** is generated by the NRPS enzymes PipA and PipB via canonical NRPS biochemistry. While **3a** might be released from PipB through premature hydrolysis, it is normally connected to the PipC‐bound ivprolide **1a** by the ester‐forming activity of the PipB‐TE (Figure 6). Comparable ester‐forming TE domains are also found in other peptide NPs like the Gq protein inhibitor FR900359 from *Chromobacterium vaccinii* ^[^ ^47^ ^]^ and salinamide from *Streptomyces* CNB‐091.^[^ ^48^ ^]^ Release of the PipC‐bound ester intermediate by the PipC‐TE yields the first full‐length product pseudotetraivprolide **4a**. *N*‐terminal acetylation by PipH to form **4c** is required also for PipE activity (Figure S1.15). PipE introduces the hydroxyl group at the C‐terminal proline ring, yielding **4d**. At this stage, transesterification between the two hydroxyl groups may generate two isomeric forms (Figure S1.2), having nearly identical MS^2^ spectra (Figure S2.9). Finally, **4d** is acetylated by the PipDFG complex to form the fully modified product **4e**.

Complementation experiments with plasmid‐encoded individual genes (*pipD*‐*pipH*) in Δ*pipDEFGH* mutant demonstrated that only PipH, and not PipE, acts directly on the core structure **4a** (Figure S1.7), suggesting that N‐terminal acetylation facilitates subsequent enzymatic modifications. Strikingly, PipH is present only in strains producing compounds with a free N‐terminus and absent from DRL BGCs in which the N‐terminus is already acetylated or carboxylated, as in detoxin and chitinimide, respectively (Figure 1c). This observation supports the hypothesis that a free N‐terminus, as in **4a**, might hinder further modifications by PipDEFG.

A co‐cultivation experiment with Δ*pipC*‐pCEP*pipA* (producing only small amounts of **3b** but possessing functional PipDEFGH) and Δ*pipE*‐pCEP*pipA* (accumulating **4c** but unable to hydroxylate or acetylate it further) resulted in significant production of **4e**. This suggests that unmodified **4c** can be secreted by the Δ*pipE* strain and subsequently taken up by the PipDEFGH‐containing strain for further modification (Figure S1.16) supporting post‐NRPS hydroxylation and acetylation.

In closely related Pip BGCs from *Pseudomonas viridiflava* DSM 11124 and *Pseudomonas syringae* DSM 50272 *pipDFG* is absent (Figure 1c and Figure S1.17). Notably is a truncated C_starter_ domain in PipA and a MbtH‐encoding gene in *P*. *viridiflava*. As expected, extracts from promoter exchange mutants of these strains exhibited compound profiles similar to those of *P*. *entomophila*, except that **4e** was not produced and **4d**/**4dʹ** accumulated as the final products (Figure S1.18). Expression of *pipDFGH* from *P*. *entomophila* in *P. viridiflava* resulted in high‐level production of **4e** (Figure S1.19), raising the question about the presence of an alternative acetylation mechanism or the biological role of non‐acetylated DRL derivatives in this strain.

When a BGC (homologs of *pipBC*; Table S1.9) from *Streptomyces flavogriseus* (*Sf*) with shares close similarity with a detoxin‐producing BGC, was expressed in *E. coli*, the non‐*O*‐acetylated derivatives **6c**‐**6e** were produced. Compounds **6c** and **6e** were also detected in *S. flavogriseus* carrying the *Sf*‐*pipE*, but no hydroxylated variants were observed (Figure S1.20). Hydroxylation of these derivatives to **6** **g** could only be detected in *E. coli* with the *Sf‐pipE* homolog but not with *P. entomophila pipE* (Figure S1.21). No subsequent acetylation was observed in *E. coli* even when plasmid‐encoded *pipDFG* was provided (data not shown). However, complementation of the Δ*pipC* mutant with *Sf‐pipC* led to an increased production of **5e** relative to **4e** (Figure S1.22), consistent with the Val preference of Sf‐PipC (Figure S1.21, compare **6c**/**6d** ratio). Interestingly, compounds **6c** and **6d** showed no protective activity against blasticidin S (Figure S1.3), suggesting that the *O*‐acetyl group is essential for the biological function of DRL‐derivatives with short N‐termini.

**Figure 7:**
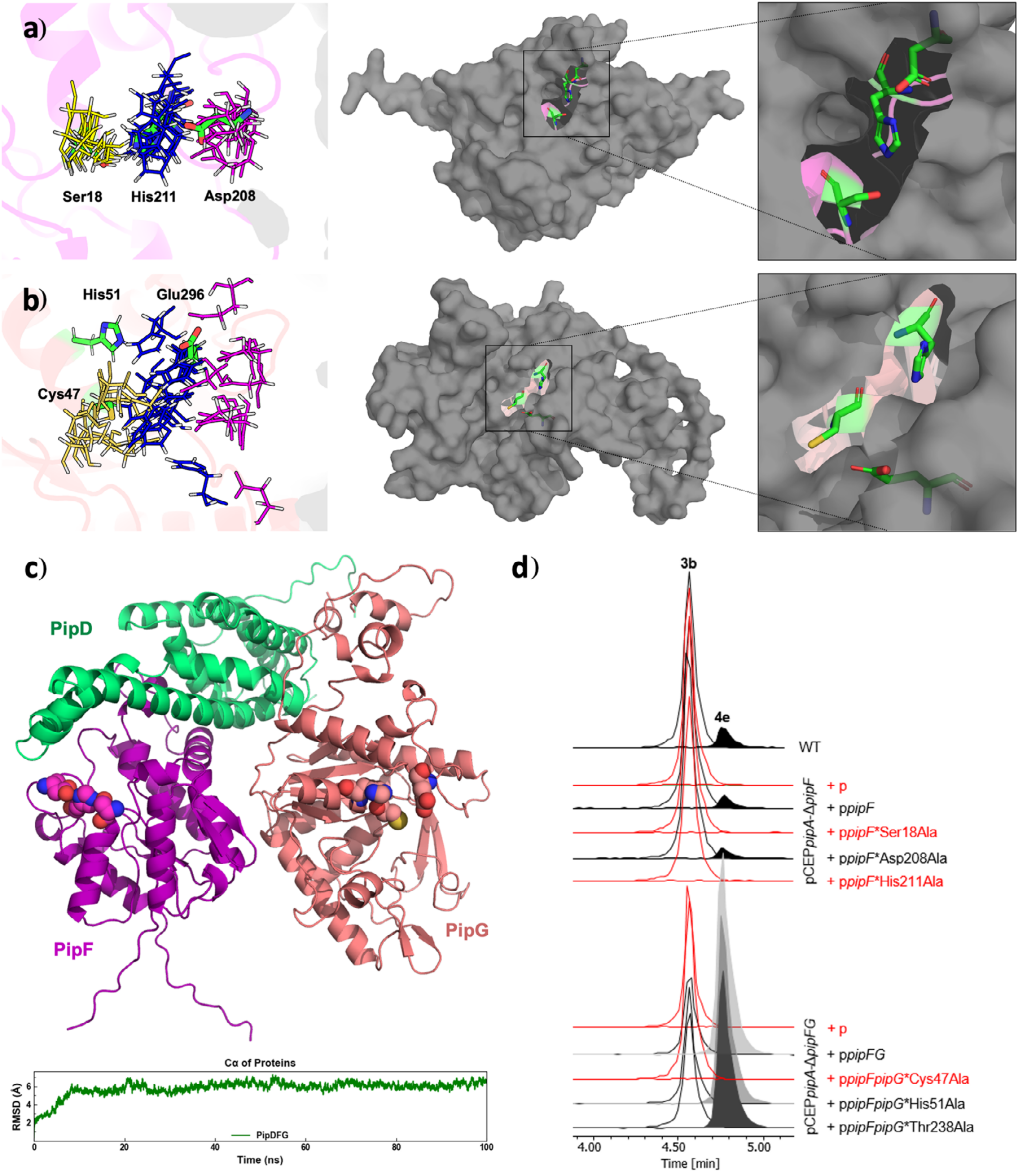
Proposed active sites of PipF and PipG and structure of the PipDFG complex. Proposed active sites of PipF **a**) and PipG **b**) are aligned with the active sites of enzymes identified in the FoldSeek results from the PDB100 dataset (left). The yellow, blue, and purple lines represent the residues corresponding to the nucleophilic, basic, and acidic components of the catalytic triad, respectively. Green sticks indicate the catalytic triad of PipF and PipG, respectively. Position and surface exposure of the catalytic triads in PipF and PipG is shown at the right. **c**) Top: Trimeric complexes of PipD, PipF and PipG as modelled by AlphaFold. The catalytic triads of PipF and PipG are shown as spheres. Bottom: RMSD of Cα in trimeric complexes. **d**) Production of **4e** in variants of PipF and PipG with specific amino acid exchanges confirming the importance of Ser18 and His211 in PipF and Cys47 in PipG, respectively. Lack of **4e** production is shown in red (WT = pCEP*pipA*).

Since Δ*pipD*, Δ*pipF* and Δ*pipG* mutants all showed nearly identical production profiles with accumulation of **4d** as final product (Figure S1.6 and S1.7), all three enzymes were suggested to participate in the terminal acetylation step. As no obvious homology to acetyltransferases was detected for any of the three proteins via BLAST‐P search, we performed CLEAN^[^ ^49^ ^]^ (Table S4.1) and structure‐based FoldSeek^[^ ^50^ ^]^ analysis (Table S4.2 and S4.3), suggesting that PipF and PipG might indeed function as hydrolases, while PipD shows some similarity to an endopeptidase (see *Supporting Information* *4* *: Prediction of structure and function of PipD, PipF and PipG*). For PipF and PipG, active sites for such hydrolytic activities could be predicted based on structural alignment with known enzyme active sites (Figure 7a,b, Figures S4.1 and S4.2) and a trimeric complex of PipDFG was also proposed based on an AlphaFold model, which appears highly stable as indicative from MD simulations over 100 ns (Figure  7c, Figure S4.3). The surface‐accessible catalytic triad in PipF may consist of Ser‐18, His‐211 and Asp‐208. In PipG, the most likely surface‐accessible catalytic triad may consist of Cys‐47, His‐51, and Glu‐296, whereas Ser‐46 or Thr‐238 are unlikely candidate, as they are not equally surface‐accessible (Figure S4.2). In order to test the role of these amino acids, Ser18Ala, His211Ala, and Asp208Ala variants of PipF and Thr238Ala, Cys47Ala, and His51Ala variants of PipG were generated and compared to the parent variants of both enzymes in vivo, confirming the importance of Ser‐18 and His‐211 in PipF and an unexpected role for Cys‐47 in PipG (Figure 7d). The large distance between the identified important residues in PipF and PipG suggests that PipDFG might either occur at a higher oligomerization state (Figure S1.23) or that Cys‐47 has another function, which cannot be clarified without X‐ray structure of the complex ideally with **4d** as the substrate for the acetylation.

## Conclusions

We have elucidated the structure and biosynthesis of the pseudotetraivprolides, novel members of the widespread DRL family of NPs found in *Pseudomonas*. Through systematic mutant construction and complementation, heterologous expression and in vitro experiments, we propose for the first time the specific functions for all enzymes involved in the biosynthesis of this widespread NP class.

Although the precise timing of individual biosynthetic steps may vary between organisms,^[^ ^24^, ^26^, ^27^ ^]^ our work provides a framework for detailed comparative analysis of DRL pathways in different organisms, which might point to different evolutionary adaptations. We demonstrate that DRL biosynthesis depends on the primary metabolism enzyme FabD, revealing an unexpected functional link between primary and secondary metabolism.

We also provide the first evidence for a PipDFG complex mediating the acetylation step, which can yield both *O*‐acetylated (**4e**) and *N*‐acetylated (**4h**) products (Figure S2.16). Further structural and biochemical investigations are required to clarify the precise catalytic and structural roles of these three enzymes in this seemingly simple yet mechanistically intriguing modification.

Finally, we confirm that only completely modified pseudotetraivprolides **4e** and **5e**, bearing the *O*‐acetyl group, fully protect against blasticidin S (Figure S1.1), whereas the *O*‐acetyl‐deficient derivative **4c** is less active. Furthermore, the *O*‐acetyl group seems to be essential for DRL‐derivatives with short N‐termini as seen in detoxin‐type molecules, whereas the longer N‐terminus of pseudotetraivprolide may compensate for its absence (Figure S1.3).

The ecological role of DRL for their producers, however, remains to be solved. The observed protective effect for *B. cereus* against blasticidin S is probably only a proxy for their true function. Further studies are needed to identify the actual molecular targets of DRL compounds in both *B. cereus* and their producers. Previous work in *E. coli* identified transporters involved in blasticidin S resistance^[^ ^29^, ^30^ ^]^ which may also serve as potential targets modulated or inhibited by DRL molecules to protect the producer.

## Author Contributions

E.B., J.B. and P.H. constructed all strains, which were analyzed by E.B. K.B. and U.K. planned and conducted the chemical synthesis. Y.N.S. isolated **1a** and **4c** and elucidated the structure also of **4e** by NMR together with Y.M.S. S.R., Z.C. and M.G. performed the enzymatic analysis of the transacylation reaction, the structural modeling of PipC and the bioinformatics characterization of PipDFG. B.P. isolated *S. flavogriseus* and K.H. and P.G. isolated **6a**‐**6c**. E.B. and H.B.B. wrote the paper with input from all authors.

## Conflict of Interests

The authors declare no conflict of interest.

## Supporting information



Supporting information

Supporting information

Supporting information

Supporting information

## Data Availability

All data and materials can be found within the manuscript, supporting information or can be requested from the corresponding author. There are three files of Supporting Information‐1 (material and methods, Tables S1.1‐S1.8, Figures S1.1–S1.19), Supporting Information‐2 (compound identification and structure elucidation of all identified derivatives and NMR data of **1a**, **4c** and **4e**; Figures S2.1–S2.32), Supporting Information‐3 (chemical synthesis of **4c** and **5c**) and Supporting Information‐4 (PipDFG structure/function prediction; Tables S4.1–S4.3, Figures S4.1–S4.3 and supplementary notes for structure/function prediction).
