# Supplementary material for: Pseudotetraivprolides from Pseudomonas entomophila Provide Insights into the Biosynthesis of Detoxin/Rimosamide‐Like Anti‐Antibiotics
Source: Angew Chem Int Ed Engl. 2025 Dec 12;65(4):e13287. doi: 10.1002/anie.202513287 (PMC12828459; doi:10.1002/anie.202513287)
Supplement: Supplementary file 1 — Supporting information [file ANIE-65-e13287-s001.pdf]

Supporting Information SI-1: Microbiology

Materials & Methods

Cultivation conditions

All *E. coli* strains were cultivated on LB agar plates or in LB medium (10 g/L tryptone, 5 g/L yeast extract, 5 g/L NaCl, for solid plates 1,5 % (*w/v*) agar-agar was added to the medium) shaking with 200 rpm at either 28 or 37°. For *E. coli* ST18 cells the LB media were supplemented with 50 µg/mL δ-aminolevulinic acid (ALA). If needed, antibiotics were added to the media at final concentrations: kanamycin, 50 µg/mL; chloramphenicol, 34 µg/mL; gentamycin, 10 µg/mL; and spectinomycin, 100 µg/mL.
P. entomophila was grown on LB agar plates or LB medium at 28-30 °C. To prevent the cells from swarming, the agar-agar concentration in solid media was increased to 2.5 -3 % (*w/v*). When required, antibiotics were added to the medium in appropriate concentrations: kanamycin, 50 µg/mL; gentamicin, 75 µg/mL for *P. entomophila*, 5 µg/mL for *P. viridiflava* and 2 µg/mL for *P. syringae*.

Production cultures

For small-scale production cultures of *P. entomophila* and *E. coli* strains, XPP medium was used as described[1]. In general, cells were first cultivated overnight in 5 ml LB medium at 28 °C, shaking at 200 rpm. For analytical samples 5 ml of XPP medium were inoculated with 50 µl of the overnight culture. For induction of the P_{BAD} promoter L-arabinose was added to final concentration of 0.2 %. For *P. syringae* and *P. viridiflava* L-arabinose was added to a final concentration of 2 %. The cultures were grown for 48 to 72 h at 28 °C shaking at 200 rpm. Production cultures from *S. flavogriseus* were grown in 10 mL TSBY medium (TSB powder 30 g/L, Yeast extract 5 g/L, and sucrose 103 g/L, pH was adjusted to 7.3). Incubation was performed at 28 °C with shaking at 200 rpm.

DNA isolation

Genomic DNA from *Pseudomonas* strains and *S. flavogriseus* was purified according to the manufacturers` protocol using either Monarch® Genomic DNA Purification Kit (NEB) or Gentra Puregene Yeast/Bact. Kit B (Qiagen).
Plasmid DNA was isolated using either the Monarch® Plasmid Miniprep Kit (NEB) or with the Invisorb® Plasmid Spin Mini Two Kit (Stratec).

Polymerase chain reaction (PCR)

For generation of DNA fragments used in the promoter exchange approach or complementation assays in all *Pseudomonas* strains, Q5® High Fidelity DNA polymerase (New England Biolabs) was used according the manufacturers` instructions. One µL of gDNA was added to 25 µL PCR reaction mix. The addition of 0.5 µL $MgCl_2$ [50 mM] and up to 3 % DMSO increased the yield of the desired fragments. PCR was performed with Lab Cycler Gradient (SensoQuest GmbH) or peqSTAR 96X Universal (VWR Peqlab) thermocyclers. Promoter exchange mutants were verified by colony PCR using Phire® Green Hot Start II Polymerase (Thermo Scientific). In a first step cells were lysed in 25 QuickExtract™ DNA Extraction Solution (Lucigen). One µL of the lysate was used as a template in the PCR reaction mix, 4 % DMSO and 0.5 µL and $MgCl_2$ [50 mM] were added to increase the final yield. In all PCR reactions the initial denaturation at 98 °C was performed for two minutes and further conducted as recommended in the manufacturers` protocol.

E. coli S17-1 mutants, carrying pCEP promoter exchange constructs were verified using BioMix™ Red (meridian BIOSCIENCE™) following the manufacturers` instructions. In a first step, cell material was resuspended in 25-30 µL 0.02 M NaOH, and lysed at 99 °C for several minutes. One µL of the lysate was used as a template in the PCR reaction mix. All primers used in this study are listed in Tables S1.4-S1.9.

Preparation of extracts

If not described differently, compounds of respective cultures were extracted by mixing 100 ml of a 48 or 72 h culture with 400 ml acetonitrile. Bacterial debris was pelleted via centrifugation for 30 min at maximum speed. An aliquot of the resulting supernatant was transferred into an HPLC vial and subjected to LC-MS analysis.

Heat map

Strains for heat map analysis were cultivated in triplicate. Five mL XPP medium was inoculated at a 1:100 ratio from an overnight culture. Metabolite production was induced by the addition of 0.2 % L-arabinose. The cultures were incubated for three days at 28 °C, shaking at 200 rpm. Following incubation, samples from the cultures were diluted at an 1:5 ratio with acetonitrile and subjected to HPLC/MS analysis. EICs were determined for all derivatives and mean values were calculated.

Assembly of pCEP and pEB17 constructs and conjugation

Construction of plasmids for performing promoter exchanges or gene deletions were performed as previously described[1,2], using the plasmids listed in Table S1.3. The vectors pCEP and pEB17 can be propagated only in *E. coli* ST18 and *E. coli* S17-1λpir cells but not in *Pseudomonas* cells.

Construction of pCEP_*xyz* (listed in Table S1.5) for promoter exchange:

Briefly, the first 300-800 bp of the gene of interest were amplified via PCR with primers containing overlapping regions to the vector backbone listed in Table S1.5. PCR was performed using the Q5® High Fidelity DNA Polymerase (NEB) following the manufacturers` protocol for DNA with high GC content optimized by adding of up to 3 % DMSO and 2 % $MgCl_2$ [50 mM] in 25 µl PCR reaction mixture. The resulting fragment was separated in an agarose gel. The fragment was cut out from the gel and extracted using Qiagen MinElute® Gel Extraction Kit or Wizard SV Gel and PCR Clean-Up System (Promega) following the manufacturers instruction. The purified fragment was assembled with PstI and BglII linearized pCEP-km vector backbone by NEBuilder® Hifi DNA Assembly Mix (New England Biolabs). The assembled pCEP-*xyz* construct was transformed either in *E. coli* ST18 or into *E. coli* S17-1 λpir respectively.

The construction of pEB17-Δ*xyz* plasmids for deletion followed the procedure described previously[1]. Briefly, 800-1500 bp upstream and downstream of the target gene(s) including overlapping sequences were amplified using primers listed in Table S1.5. The pEB17 vector backbone was generated with restriction enzymes PstI and BglII. The PCR fragments with overlaps of 25-30 bp to each other and to the vector backbone were ligated by NEBuilder® HiFi DNA Assembly Master Mix according to the manufacturers` protocol and subsequently transformed into *E. coli* ST18 via electroporation.

E. coli clones carrying ligated pCEP-*xyz* or pEB17-Δ-*xyz* constructs were verified by colony PCR using primers VpCEP-fw and VpCEP-rv for pCEP and VpDS132-fw and pCEP-rv for pEB17 constructs, respectively. All primers used for verification are listed in Table S1.4.

The pCEP-gene xyz or pEB17-Δgene xyz plasmids (all vectors used in this study are listet in Table S1.5) were transformed into *P. entomophila* via conjugation adapted from[3]. Briefly, the recipient *Pseudomonas* strain was cultivated in 5 mL LB medium, and the selected *E. coli* donor strain was grown in 5 ml LB medium over night with

appropriate antibiotics and, for ST18 cells, supplemented with δ-aminolevulinic acid (ALA). The next day, donor cells were washed twice in LB medium to eliminate ALA or antibiotics. Donor and recipient cells were adjusted to an OD_{600} of 6.0 and 1 ml donor and 100 µl recipient cells were mixed and pelleted by centrifugation for 1 min at 21,000 *g*, resuspended in 50 µl LB medium and spotted as a single drop on an LB agar plate. The inoculated plate was incubated at 28 °C for 5-6 hours to allow conjugation and transfer of the plasmid via rolling circle mechanism. The cell plaque was scraped from the plate using a sterile inoculation loop and dissolved in 500 µl LB medium. Serial dilutions from 1:20 – 1:100 were prepared and 50 µl of each dilution was plated on LB medium containing kanamycin as selective antibiotic. If *E. coli* S17-1 λpir cells were used as donors, additional ampicillin in a final concentration of 100 µg/ml was also added for counter selection. The inoculated plates were incubated for 24 to 48 hours at 28 °C. The obtained colonies for promoter exchange mutants were verified by colony PCR using VpCEP-fw and a gene-specific verification primer (Table S1.4 and S1.5).

Counterselection and verification of deletion strains

Single colonies of the generated insertion mutants of *Pseudomonas* pEB17-Δ*xyz* were streaked onto no-salt LB agar (NSLB) containing 15 % sucrose[1,4]. The addition of 3 % agar-agar (*w/v*) was necessary to prevent cells from swarming. The plates were incubated at 18 °C for 24-72 h to facilitate a second homologous recombination event resulting in the loss of the vector backbone mediated by the activity of SacB, encoded on the pEB17 vector. The obtained colonies were plated on LB agar and on LB agar containing kanamycin. Clones growing exclusively on LB agar (kanamycin sensitive) were verified by colony PCR using strain-specific verification primers listed in Table S1.5.

Anti-antibiotic activity

Preparation of extracts

The strains EB7001 (WT), EB862 (ΔPELP4_pCEP*pipA*), EB849 (ΔPELP4), (listed in Table S1.2) were used in this study. They were cultivated for 24 h at 28 °C with shaking at 200 rpm in 5 mL LB with 50 µg/mL kanamycin, if needed. To set up a production culture, 100 mL XPPM, supplemented with 2 % XAD-16 adsorber resin, were

inoculated with 1 mL from this pre-culture. Prior to inoculation, the cells were washed twice to eliminate kanamycin. All strains were induced with 0.2 % L-Arabinose. The cultures were incubated for 72 h at 28 °C and 200 rpm. Following incubation, the XAD resin was collected and extracted with 300 mL methanol. The solvent was evaporated to dryness to obtain an oily extract, which was dissolved in methanol to a final concentration of 20 mg/mL. For the anti-antibiotic assay cellulose discs were prepared with 10 µL blasticidin S [1 mg/mL] dissolved in water together with varying concentrations of extracts derived from 862, 7001 and 849.

*Isolation of **6c** and **6d** for bioactivity testing*

E. coli DH10B::mtaA_pEB175+pEB176 (EB 7181) (Table S1.10) was cultivated in 6x 500 mL XPP medium containing 2 % XAD-16 adsorber resin and 0.1 % L-arabinose for induction. The cells were incubated for 48 h at 30 °and 130 rpm. Following the cultivation, the resin was collected and defatted with cyclohexane (3x 500 ml). By using three sequential extractions with 500 mL acetonitrile, the target compounds were eluted from the resin with a final yield of 660 mg. The solvent was lyophilized with a Büchi L-300 Lyovapor and further purified by HPLC on an Agilent Autoscale system with acetonitrile/water gradient containing +0.1 % formic acid (flow rate 20 mL/min, gradient 10-80 % acetonitrile). The purification resulted in a final yield of 3.8 mg for **6c** and 49 mg for **6d**.

Bioactivity assay

The screen for biologic activity was performed as described previously[5]. *Bacillus cereus* ATCC14579 was cultivated in 10 mL ATCC medium 3 (beef extract 3 g/L, peptone 5 g/L, agar 15 g/L, pH adjusted to 7.0) 24 h at 30 °C and 200 rpm. Subsequently 20 µL of the *Bacillus* culture were spread on ATCC medium 3 agar plates. Prepared discs were put onto the fresh inoculated plates. Incubation followed for 24 h at 30 °C.

Cloning of pSEVA constructs

Complementation of deleted genes was achieved by cloning the respective gene into free replicating conjugatable plasmid pSEVA621-Gm[6]. The plasmid backbone was generated via PCR using primers AR-900-fw and AR-970-rv (Table S1.3 and S1.4.). The DNA fragments were assembled with PCR generated pSEVA backbone using

NEBuilder® HiFi DNA Assembly Master Mix. The assembled plasmid was transformed into *E. coli* ST18 cells via heat shock or electroporation. The constructs were verified by colony PCR, using primers V_pCEP_fw and VPEB-796-pSEVA-rv listed in Table S1.4. For complementation of the corresponding deletion mutant plasmids were introduced via conjugation. For each deletion mutant an empty plasmid control was generated. All primers used for generating the pSEVA constructs and their respective strains are listed in Table S1.8.

Plasmids pEB163, pEB164, pEB165, pEB166 and pEB171 were constructed using the KLD Enzyme Mix (NEB) using pEB145 as template for fragment generation, except for pEB170, which was amplified using pEB166 as the template. The resulting constructs were transformed into *E. coli* ST18 and verified by full plasmid sequencing. The primers and *resulting E. coli* mutants are listed in Table S1.8. Verified plasmids were conjugated into 2470, and all resulting *P. entomophila* strains are listed in Table S1.2. Extracts of production cultures of these strains were investigated by HPLC/MS, as described previously.

Genes from *S. flavogriseus* were amplified with primers listed in Table S1.10 and Q5® High-Fidelity DNA Polymerase (NEB) following the manufacturer´s protocol optimized for GC-rich templates. The resulting PCR fragments were cloned into the vector pSEVA621-GM via their homologous flanking regions using NEBuilder® HiFi DNA Assembly Master Mix (NEB). The assembled plasmids were transformed into *E. coli* ST18 generating pSEVA derivatives listed in Table S1.10. These donor strains were subsequently conjugated with selected *P. entomophila* recipient strains to generate transconjugants listed in Table S1.2.

For metabolite production, *P. entomophila* strains were grown in 5 mL XPP medium inoculated at a 1:100 ratio from overnight LB pre-cultures. Expression was induced with 0.2 % L-arabinose and cells were gown for three days at 28 °C and 200 rpm. After cultivation, the cultures were extracted with acetonitrile, and the resulting extracts were prepared for HPLC-MS analysis as described previously.

Cloning of pACYC and pCOLA constructs and heterologous expression

For cloning of *pipC*, *pipCD* and *fabD*, the respective genes were amplified with primers listed in Table S1.6. The amplified fragments were ligated into PCR generated pACYC_ara or pCOLA_ara backbones, respectively, using the NEBuilder® Hifi DNA

Assembly Mix (NEB). The resulting constructs were transformed into *E. coli* DH10B::*mtaA* via electroporation. All generated mutants are listed in Table S1.6.

For heterologous expression of p*pipC*, p*pipCD* and p*fabD*, the respective mutants were cultivated overnight in 5 mL LB medium which the appropriate antibiotics. The following day, 5 mL of fresh LB medium with antibiotics, was inoculated with 50 µL of the corresponding overnight culture. Gene expression was induced with 0.2 % L-arabinose, and cultures were incubated for 24 h at 22 °C with shaking at 200 rpm. Culture extraction was performed as described above.

For cloning of genes from *S. flavogriseus* (*pgaptmp_006718 (Sf-pipB)*, *pgaptmp_006719 (Sf-pipC* and *pgaptmp_006720 (Sf-pipE)*), the respective gene fragments were amplified using primers listed in Table S1.10 and assembled via their homologous arms into the plasmid backbones pCOLA_ara, pACYC_ara or pCDF_ara using the NEBuilder® HiFi DNA Assembly Master Mix (NEB). The resulting constructs, (Table S1.10) were transformed into *E. coli* DH10B::*mtaA* by electroporation.

For heterologous expression 5 mL XPP medium was inoculated (1:100) with an 5 mL LB overnight culture and induced with 0.2 % L-arabinose. Cultures were cultivated for 48 h at 22 or 28 °C with shaking at 200 rpm.

Cross feeding experiment via co-cultivation

P. entomophila strains pCEP*pipA*, Δ*pipC*-pCEP*pipA* and Δ*pipE*-CEP*pipA* were cultivated in 5 mL LB medium supplemented with 50 µg/mL kanamycin at 28 °C and 200 rpm for 24h. For co-cultivation experiments 5 mL of fresh XPP medium were inoculated with 50 µL of the Δ*pipC*-pCEP*pipA* culture and 50 µL of the Δ*pipE*-CEP*pipA* culture. As controls, each strain was also cultivated separately under the same conditions. All cultures were induced with 0.2 % L-arabinose and incubated for 72 h at 28 °C with shaking at 200 rpm.

Inserting point mutations

A point mutation in the thioesterase (TE) domain of *pipB* was introduced by substituting the catalytic serine residue with alanine (codon change TCG>GCG) using overlapping primers listed in Table S1.5. Point mutations in the catalytic triad of PipF

and PipG were generated by PCR with primers listed in Table S1.7, replacing the respective codons with the alanine codon GCG. Plasmids p*pipF* and p*pipFG* (Table S1.7) served as PCR template. The resulting PCR fragments were assembled using KLD Enzyme Mix (NEB), and the obtained plasmids were verified by sequencing. Verified constructs were transformed via conjugation into Δ*pipF*-pCEP*pipA* and Δ*pipFG*-pCEP*pipA*, respectively. Productions cultures of respective mutants were analyzed as described above.

Heterologous expression in *E. coli*

For in vitro enzyme kinetic analysis, *P. entomophila* FabD, *E. coli* FabD and PipC ACP were expressed in *E. coli* BAP1 cells. For transformation, 40 µL of cells were mixed with 0.5 µL plasmid. Electroporation was carried out in a 0.2 cm electroporation cuvette 25 µF, 200 Ohm, 2.5 kV). 350 µL preheated SOC medium (super optimal medium with catabolite repression) was immediately added to the cell suspension. The mixture was incubated at 37 °C for 60 min. The cell suspension was centrifuged (3,000 x g, 2 min), resuspended in 100 µL SOC medium, plated on LB-agar plates supplemented with ampicillin (100 µg/mL) and incubated at 37 °C overnight.

PipC apo-ACP was expressed in BL21 gold *E. coli* cells (Novagen). Transformation was carried via heat shock at 42 °C for 20 sec. All following steps have been described above.

For expression, 20 mL LB-medium (supplemented with 100 µg/mL ampicillin) pre-cultures were inoculated with five clones from LB-agar plates. The cultures were incubated at 37 °C and 180 rpm for 4 - 5 hours. Pre-cultures were used to inoculate 1 L TB medium expression cultures (24 g/L yeast extract, 12 g/L tryptone, 16.4 g/L K2HPO4 · 3 H2O, 2.3 g/L KH2PO4, 0.5% glycerol) supplemented with 100 µg/mL ampicillin. Expression cultures were incubated at 37 °C and 150 rpm until an OD600 between 0.2 – 0.3 was reached and were further incubated at 18 °C and 150 rpm until an OD600 of 0.6 to 0.8 was reached. Protein expression was induced by adding 250 µL 1 M isopropyl β-d-1 thiogalactopyranoside (IPTG) and carried out at 18 °C and 150 rpm overnight.

Protein purification

For cell harvest, expression cultures were transferred into 1 L Beckmann Culture bottles and centrifuged at 4,500 x g for 20 min (Beckmann Coulter Avanti J20-XP, rotor: JLA-8.100). The supernatant was discarded and cell pellets were transferred into 50 mL falcons. Lysis buffer (50 mM NaP_i, 450 mL NaCl, 20 mM imidazole, 10% glycerol, pH 7.6) was added to a total volume of 35 mL and the cell pellets were thoroughly resuspended. 75 µL 0.5 M ethylenediamine-tetraacetic acid (EDTA) and DNAse were added. The cells were disrupted using a French Pressure Cell. The cell lysates were separated from cell debris via

centrifugation at 50,000 x g at 4 °C for 40 min (rotor: JA 25.50). 75 µL 1 M $MgCl_2$ were added to the supernatant prior to protein purification.

For protein purification a two-step protocol was used. The crude extract was applied to an equilibrated His-Ni-NTA resin (gravity flow columns, Takara Inc., CV = 5 mL). The flowthrough was discarded. The column was washed with 25 mL lysis buffer and with 10 mL Wash buffer (50 mM NaP_i, 450 mM NaCl, 60 mM imidazole, 10% glycerol, pH 7.6). The protein was eluted with 25 mL elution buffer (2x 12.5 mL) (50 mM NaP_i, 450 mM NaCl, 250 mM imidazole, 10% glycerol, pH 7.6). Elution samples were concentrated using centrifugal filters (Merck, MWCO = 10 kDa or 30 kDa) and subsequently applied to size exclusion chromatography (SEC) for further protein polishing. The SEC buffer (250 mM KP_i, 10% glycerol, pH 7.0) was filtered and degassed before use and columns were equilibrated with 2 CV. A Superose^{TM} 6 Increase 10/300 GL column (cytiva) equipped on ÄKTA Go system was used for polishing of FabD. A Superdex^{TM} 200 Increase 10/300 GL column (cytiva) equipped on ÄKTA Basic was used for polishing of PipC ACP. Prior to injection via capillary loop, the protein samples were filtered using a 0.22 µm Ultrafree MC-CG Durapore PVDF filter. Elution fractions were identified via detection of UV absorption at 280 nm. Fractions were pooled, concentrated, frozen in liquid nitrogen and stored at -80 °C.

PipC holo-ACP synthesis

PipC apo-ACP was phosphopantetheinylated with Sfp in vitro. For this, 250 µM CoA-SH, 25 µM apo-ACP and 10 µM Sfp were mixed in phosphopantetheinylation buffer (50 mM HEPES, 200 mM NaCl, 10 mM $MgCl_2$, pH 7.0; total volume: 25 µL) and incubated at 37 °C for 10 min.

Urea PAGE analysis

Analysis of PipC apo-ACP and holo-ACP was performed with urea PAGE. According to a literature protocol (https://doi.org/10.1002/anie.202412195), gel electrophoresis was conducted with a polyacrylamide gel that contained urea. The separating gel contained: 15% acrylamide/bisacrylamide, 1.12 M Tris, 7.5 M urea, 0.1% (v/v) N,N,N',N'-tetramethylethyldiamine and 0.03% (w/v) ammonium persulfate. The loading dye (2x) contained 1% bromphenol blue, 25% glycerol, 62.5 mM Tris, 2 M urea and 10 mM N-ethylmaleimide (NEM). Samples (conc.: 25 µM PipC ACP) were incubated in loading dye (2x) for 10 min at room temperature and immediately applied to the gel. The electrophoresis was performed at 70 V for 15 min followed by 200 V for 1 – 1.5 h with running buffer (25 mM Tris, 200 mM glycine) and cooling. The gel was stained in an aqueous solution of 0.1% Coomassie Blue R-250, 50% methanol and 10% acetic acid overnight. For destaining, an aqueous solution of 10% methanol and 10% acetic acid was used.

α-KGDH-coupled activity assay

Measurements of the FabD-mediated transacylation reaction were carried out as previously described (https://doi.org/10.1038/s41557-022-00996-z). The assay was performed in 384-well Small Volume HiBase Microplates (Greiner Bio-one) with plate reader settings as follows (ClarioStar, BMG labtech): excitation: 348-20 nm; emission: 476-20 nm; gain: 1600; focal height: 11.9 mm; flashes: 10; orbital averaging: off. Equidistant measurements were taken every 5 s. Monitoring was conducted for 5 min at 25 °C. Measurements were recorded in technical triplicates and the background noise (initial velocity of self-acylation; assay mixture without AT domain) was subtracted. Four solutions were prepared 4-fold stocks in assay buffer (50 mM sodium phosphate, 10 % glycerol, 1 mM EDTA, pH 7.6), filtered and degased) that were combined manually prior to the measurement to give a total assay volume of 20 µL. Solution 1 comprised the trans-AT domain (supplemented with 0.1 mg mL^{-1} BSA). Solution 2 contained 8 mM α-ketoglutaric acid, 1.6 mM NAD^{+}, 1.6 mM TPP and 60 mU/100 µL αKGDH. Solution 3 included Mal-CoA and Solution 4 contained PipC ACP. Final concentrations of proteins were 0.5 - 3 nM *P. entomophila* FabD or *E. coli* FabD, 1 - 40 µM PipC ACP and 0.5 - 50 µM Mal-CoA.

Supplementary Tables S1

Table S1.1. Genes and putative functions in the *pip* gene cluster and other genes investigated in this study. Asterisk indicate adaptation suggested by antiSMASH.

name	locus tag	lengh [bp]	no. of amino acids	protein id	function
pipA	PSEEN_RS12600	8973	2990	WP_011533898.1	NRPS
pipB	PSEEN_RS12605	4116	1371	WP_011533899.1	NRPS
pipC	PSEEN_RS12610	9036	3011	WP_011533900.1	NRPS-PKS
pipD	PSEEN_RS12615	675*	224	WP_158020252.1	hypothetical protein
pipE	PSEEN_RS12620	870	289	WP_011533902.1	TauD/TfdA dioxygenase family protein
pipF	PSEEN_RS12625	723*	240	WP_158020253.1	hypothetical protein
pipG	PSEEN_RS12630	1035	344	WP_011533904.1	GSCFA-domain containing protein
pipH	PSEEN_RS12635	519	172	WP_011533905.1	GNAT family N-acetyltransferase
fabD	PSEEN_RS07520	939	312	WP_011532885.1	[acyl-carrier-protein] S-malonyltransferase
hfq	PSEEN_RS22860	261	86	WP_011535948.1	RNA-binding protein Hfq
pipA	GHKPGMKM_08200	9066	3021	-	NRPS
pipA	LMDDJHCF_09560	9711	3236	-	NRPS
Sf-pipA	pgaptmp_006718	4023	1340		NRPS
Sf-pipB	pgaptmp_006719	9438	3145		NRPS-PKS
Sf-pipE	pgaptmp_006720	891	296		TauD/TfdA dioxygenase family protein

Table S1.2. Strains used in this work.

Name	Strain	Reference
HBLC 395/EB 7001	*Pseudomonas entomophila* L48 DSM 28517	[7]
PG 4253	*Bacillus cereus* ATCC14579	[5]
HBEC 089	*E. coli* S17-1λpir (Tp, Smr, *recA*, *thi*, hsdRM+RP4::2-Tc::Mu::Km, Tn7, λpir phage lysogen)	Invitrogen
HBEC 118	*E. coli* ST18 (*E. coli* S17-1λpir Δ*hemA*)	[3]
HBEC 004	*E. coli* DH10B (F-mcrA, Δ(mrr-hsdRMSmcrBC) Φ80lacZΔM15, ΔlacX74, *recA1*, endA1, araD139, Δ(ara leu)7697 galU, galK, rpsL, nupG, λ-)	[8]
HBEC 029	DH10B *entD*::*mtaA*	[9]
HBLC 386	*Pseudomonas syringae* DSM 50274	DSMZ
HBLC 388	*Pseudomonas viridiflava* DSM 11124	DSMZ
22	*Streptomyces flavogriseus*	This work
EB 849	*P. entomophila*_Δ*pvsA*Δ*eltA*ΔRS12890Δ*psmDFG*ΔRS10025-10010	This work
EB 862	*P. entomophila*_Δ*pvsA*Δ*eltA*ΔRS12890Δ*psmDFG*ΔRS10025-10010_pCEP*pipA*	This work
2506	*P. viridiflava*-pCEP*pipA* (2369)+p*pipD-H* (pEB141)	This work
EB 7050	*P. viridiflava*-pCEP*pipA*+pSEVA621	This work
2484	*P. syringae*-pCEP*pipA*(2367)+p*pipD-H* (pEB141)	This work
2486	*P. syringae*-pCEP*pipA*+pSEVA621	This work
EB 7018	*P. entomophila* Δ*pipD*Δ*pipH*-pCEP*pipA*	This work
EB 7045	*P. entomophila*_Δ*pipD*Δ*pipH*-pCEP*pipA* + pSEVA621	This work
EB 7046	*P. entomophila*_Δ*pipD*Δ*pipH*-pCEP*pipA* + p*pipH* (pEB132)	This work
EB 7048	*P. entomophila*_Δ*pipD*Δ*pipH-pCEPpipA* + p*pipD* (pEB136)	This work
2512	*P. entomophila* Δ*pipDEFGH*_pCEP*pipA* + pSEVA621-GM	This work
2514	*P. entomophila* Δ*pipDEFGH*_pCEP*pipA* + p*pipH* (pEB132)	This work
2516	*P. entomophila* Δ*pipDEFGH*_pCEP*pipA* + p*pipFGH* (pEB133)	This work
2520	*P. entomophila* Δ*pipDEFGH*_pCEP*pipA* +p*pipD* (pEB136)	This work
2522	*P. entomophila* Δ*pipDEFGH*_pCEP*pipA* + p*pipG* (pEB137)	This work
2524	*P. entomophila* Δ*pipDEFGH*_pCEP*pipA* + p*pipFG* (pEB138)	This work
2528	*P. entomophila* Δ*pipDEFGH*_pCEP*pipA* + p*pipF* (pEB140)	This work
2530	*P. entomophila* Δ*pipDEFGH*_pCEP*pipA* + p*pipE* (pEB143)	This work
2532	*P. entomophila* Δ*pipDEFGH*_pCEP*pipA* + p*pipDE* (pEB144)	This work
EB 7168	*P. entomophila* Δ*pipDEFGH*_pCEP*pipA* + p*pipEH* (pEB163)	This work
EB 7178	*P. entomophila* Δ*pipDEFGH*_pCEP*pipA* + p*pipDFG* (pEB170)	This work
EB 7147	*P. entomophila* Δ*pipDEFGH*_pCEP*pipA* + p*pipDEH* (pEB164)	This work
EB 7170	*P. entomophila* Δ*pipDEFGH*_pCEP*pipA* + p*pipDEFG* (pEB166)	This work
EB 7176	*P. entomophila* Δ*pipDEFGH*_pCEP*pipA* + p*pipDEFH* (pEB165)	This work
EB 7180	*P. entomophila* Δ*pipDEFGH*_pCEP*pipA* + p*pipEFGH* (pEB171)	This work
EB 7186	*P. entomophila*Δ*pipC*-pCEP*pipA*+pSEVA621	This work
EB 7187	*P. entomophila*Δ*pipC-pCEPpipA* + pEB177	This work
EB 7189	*P. entomophila*Δ*pipC*-pCEP*pipA* + pEB178	This work
2607	*P. entomophila* Δ*pipABC*-pCEP*pipD* + pEB180	This work
EB 7198	*E. coli*::mtaA pEB175 + pEB176 + pCDF	This work
2609	*P. entomophila* Δ*pipABC*_pCEP*pipD* + pSEVA621	This work

Table S1.3. Plasmids used in this work.

Name	Description	Reference
pEB17-Km	pDS132 based, R6K *ori*, Km^R, *ori*, *sacB*	[1,10]
pCEP-Km	pDS132 based, R6K *ori*, Km^R, P_{BAD}, *oriT*	[1,2,10]
pCOLA_ara/tacI-Km	ColA *ori*, Km^R, *araC*-P_{BAD}, *tacI*	[11]
pACYC_ara/tacI-Km	p15A *ori*, Cm^R, *araC*-P_{BAD} *tacI*	[12]
pCOLA_ara/tacI-Gm	ColA *ori*, Gm^R, *araC*-P_{BAD} *tacI*	[11]
pCDF_ara/tacI-Spec	CloDF13 *ori*, $Spec^R$, *araC*-P_{BAD} *tacI*	
pSEVA621-Gm	oriV, Gm^R, P_{BAD}, mNeonGreen, *oriT,* RiboJ	[6]

Table S1.4. General primers used in this work.

Name	Sequence	Purpose
V_pCEP_fw	GCTATGCCATAGCATTTTTATCCATAAG	verification primer for pSEVA, pCOLA, pACYC, pCDF and pCEP constructs
VPEB-796-pSEVA-rv	CATCGTTGCTGCTGCGTAAC	verification primer for pSEVA constructs
V_pDS132-rv	ACATGTGGAATTGTGAGCGG	verification primer for pEB17 and pCEP constructs
V_pDS132_fw	GATCGATCCTCTAGAGTCGACCT	verification primer for pEB17 constructs
VP-pCOLAduett-rv	GCTAGTTATTGCTCAGCGG	verification primer for pCOLA, pACYC and pCDF constructs
PEB_1028-fw	CAGCTTAATTAACCTAGGCTGCTG	backbone amplification pACYCara or pCOLA_ara_tac
PEB_1029-rv	GGAATTCCTCCTGTTAGCCCAA	
AR_900-fw	GTCGTGACTGGGAAAACCCT	backbone amplification pSEVA621-Km
AR_970-rv	CTAGTATTTCCCCTCTTTCTCTAGT	

Table S1.5. Primers for the construction of pCEP constructs for promoter exchange of selected genes and resulting *P. entomophila*-pCEP strains. Primers for the construction of pEB17 constructs for gene deletion and the resulting *E. coli* stains. *P. entomophila* deletion mutants with promoter exchange.

Name	Sequence	*E. coli* strain	Purpose	*P. entomophila* strain
PEB_518-fw	TTTGGGCTAACAGGAGGCTAGCAT_ATGCCCGACA CTTCCTCCTTG	EB535 pCEP*pipA* (pEB98)	Promoter exchange to PSEEN_RS12600	pCEP*pipA* (JB32)
PEB_519-rv	TCTGCAGAGCTCGAGCATGCACAT_CTGCAACGG GTACAGCCGTGC			
VPEB_520-rv	GCTGAACACATCGAACGAG			
PEB_580-fw	TTTGGGCTAACAGGAGGCTAGCAT_ATGAGCGTGT TCCAGGAAAC	EB592 pCEP*pipC* (pEB97)	Promoter exchange to PSEEN_RS12610	pCEP*pipC* (EB593)
PEB_581-rv	TCTGCAGAGCTCGAGCATGCACAT_TCGACAGGCA GACAAATCGAT			
VPEB_582-rv	CACCGTGATGAACAAGGTATCGAC			
JUB_1_fw	CCTCTAGAGTCGACCTGCAG_CGTGACAGCACCG GTCGAAG	JB29 pEB17_Δ*pipD* (pJuB-01)	Deletion of PSEEN_RS12615	Δ*pip*D-pCEP*pipA* (JB55)
JUB_2_rv	CTGATGGGTGTTGGCTGGCCACGTG_CTGTGCAT ATTCACCGGCCATG			
JUB_3_fw	GTTCATGGCCGGTGAATATGCACAG_CACGTGGC CAGCCAACACC			
JUB_4_rv	TCCCGGGAGAGCTCAGATCT_GTCGAGCTGGTCG ATGTGCG			
V_JUB_5_fw	AGTCGGTCTGTCGACACAGGAAG			
V_JUB_6_rv	GCGGCGCAGACACATTTGATCG			
JUB_7-fw	CCTCTAGAGTCGACCTGCAG_AAACCACGGTCATC AACAGTATCTGG	JB45 pEB17_*pipB*ΔTE (pJuB-02)	Deletion of the TE domain in PSEEN_RS12605 (*pipB*)	Δ*pipB*-TE-pCEP*pipA* (JB59)
JUB_8-rv	ATTTCCCCTCCTGGGCATCGAAACGTTGCTGGCC GTCCCCTGACCTGAA			
JUB_9-fw	ATCACCTTCAGGTCAGGGGACGGCCAGCAACGTT TCGATGCCCAGGAGG			
JUB_10-rv	TCCCGGGAGAGCTCAGATCT_AATAGGGTGGCAC AGGGCGAAGG			

V_JUB_11-fw	TACAGCCTCAAGGACGTATTCTGC			
V_JUB_12-rv	ATGCCGGTGTCCGGCAAGGGCTGG			
PEB_616-fw	CCTCTAGAGTCGACCTGCAG_GAGCTGACCCTCTACCAGATCTGG	JB20 pEB17_Δ*pipC* (pJuB-03)	Deletion of PSEEN_RS12610	Δ*pipC*-pCEP*pipA* (JB31)
PEB_617-rv	CATATTCACCGGCCATGAACACCCTCCTCCACGGTTTCCTGGAACACGCTCAT			
PEB_618-fw	GGAAATGATGAGCGTGTTCCAGGAAACCGTGGAGGAGGGTGTTCATGGCCGG			
PEB_619-rv	TCCCGGGAGAGCTCAGATCT_GTCCCGAGAAACGCCTGCATC			
VPEB_620-fw	CTACCTGTGGCTCGACAGCCTG			
VPEB_621-rv	CCAGGGCATCTCGCCGTCAT			
VPEB_582-rv	GTCGATACCTTGTTCATCACGGTG			
PEB_622-fw	CCTCTAGAGTCGACCTGCAG_GTGAGTCGCCCAACAGCAGGAAG	JB24 pEB17_Δ*pipH* (pJuB-04)	Deletion of PSEEN_RS12635	Δ*pipH*-pCEP*pipA* (JB57)
PEB_623-rv	CCTTGCCCTCCACTCTCGCTAGGAGCACCCTCGACCTGCACGGATAG			
PEB_624-fw	GTTCATGGGCTATCCGTGCAGGTCGAGGGTGCTCCTAGCGAGAGTGGAG			
PEB_625-rv	TCCCGGGAGAGCTCAGATCT_CAATGCTATGCAGCAAGACCAG			
VPEB_626-fw	CACCGCTGAACGACAGCTGC			
VPEB_627-rv	GAGCAGACTCACCTAACAATCGG			
VPEB_626.1-fw	GTACAGCCGCTCGAGTTCG			
PEB_610-fw	CCTCTAGAGTCGACCTGCAG_GATCGCCGAGGCGATCAATAGC	JB22 pEB17_Δ*pipE* (pJuB-05)	Deletion of PSEEN_RS12620	Δ*pipE*-pCEP*pipA* (JB56)
PEB_611-rv	CTGCATACCCTGAAACAAGCACCTGCGGCCTTCAGTGCCTGTTGATCCAT			
PEB_612-fw	GTATGGATCAACAGGCACTGAAGGCC_GCAGGTGCTTGTTTCAGGGT			
PEB_613-rv	TCCCGGGAGAGCTCAGATCT_GGAGGCGTGGCCTTCGTGATG			
VPEB_614-fw	CATGTGCCTGGCGTACAGGC			

VPEB_615-rv	CTACTTCCCGTCCTACGAAATCATC			
JUB_13-fw	ATCGATCCTCTAGAGTCGACCTGCAG-TTTCATCGACAGCCCGTTCGTGC	JB65 pEB17_*pipB*-S(72)A (pJuB-06)	Point mutation S(72)A in the TE domain of PSEEN_RS12605 (*pipB*)	Δ*pipB*-TE*-pCEP*pipA* (JB67)
JUB_14-rv	GCCGAACGCCAGCCCGGTGAG			
JUB_15-fw	CTCACCGGGCTGGCGTTCGGC			
JUB_16-rv	TGGAATTCCCGGGAGAGCTCAGATCT-AAATCGATCGCCCTGCTGGATATCGAT			
JUB_14.1_rv	GTAGGCCACCAGGCCGCCGAACGCCAGCCCGGT GAGCACCAG			
JUB_15.1_fw	CGGCCACTGGTGCTCACCGGGCTGGCGTTCGGC GGCCTGGTGGC			
Seq_JUB_17 _fw	TCTGCTACCTGTCGCTG			
Seq_JUB_18 _rv	AGCTTGTCGCGGAACTC			
PEB-1002-fw	GATCCTCTAGAGTCGACCTGCAG_CGAGAGTCAG ATGCTGTTGAAC	2318 pEB17_Δ*pip*G (pPH61)	Deletion of PSEEN_RS12630 (*pipG*)	Δ*pipG*-pCEP*pipA* (2334)
PEB-1003-rv	CATCTACCGCCTCGACCTGCACGGATAGCTTCCTG CTGTTGGGCGAC			
PEB-1004-fw	CTAGGTGTGAGTCGCCCAACAGCAGGAAGCTATC CGTGCAGGTCGAG			
PEB-1005-rv	GTGGAATTCCCGGGAGAGCTCAGATCT_CCAGTGT TCACATCGAGCTC			
VPEB-1006-fw	CACCTCGCATATCGTGCAG			
VPEB-1007-rv	GTTGTAGGCGTACGCCAAG			
PEB-1008-fw	GATCCTCTAGAGTCGACCTGCAG_GGATCAACAG GCACTGAAGG	2319 pEB17_Δ*pipF* (pPH62)	Deletion of PSEEN_RS12625 (*pipF*)	Δ*pipF*-pCEP*pipA* (2362)
PEB-1009-rv	GGGATTTCGGGGTGGACTTCTTCACCCTGGACCG CATCGCGGATGAATC			
PEB-1010-fw	GTAGGAGCGGATTCATCCGCGATGCGGTCCAGGG TGAAGAAGTCCAC			
PEB-1011-rv	GGATGAACTGGCTCGATG			

VPEB-1012-fw	GTTGCAACACGTGGCCAG			
VPEB-1013-rv	CAAGGACGCCATCGTCAC			
PEB_963-rv	CATCTACCGCCTCGACCTGCACGGATAGGACCGCATCGCGGATGAATC	2305 pEB17_Δ*pipFG* (pEB138)	Deletion of PSEEN_RS12625-12630 (*pipFG*)	Δ*pipFG*-pCEP*pipA* (2311)
PEB_964-fw	GTAGGAGCGGATTCATCCGCGATGCGGTCCTATCCGTGCAGGTCGAG			
PEB_965-rv	GTGGAATTCCCGGGAGAGCTCAGATCT_GTTGTGTTGTAGGCGTACGC			
VPEB-966-fw	GGCCATCGAACGTATTGCC			
VPEB-967-rv	CAGCTCACCCAATCCGATGG			
PEB-1008-fw	GATCCTCTAGAGTCGACCTGCAG_GGATCAACAGGCACTGAAGG	2305 pEB17_Δ*pipFGH* (pEB142)	Deletion of PSEEN_RS12625-12635 (*pipFGH*)	Δ*pipFGH*-pCEP*pipA* (2471)
PEB-1087-rv	CTTGCCCTCCACTCTCGCTAGGAGCACCGGGACCGCATCGCGGATGAATC			
PEB-1088-fw	GTAGGAGCGGATTCATCCGCGATGCGGTCCCGGTGCTCCTAGCGAGAG			
PEB-625-rv	GTGGAATTCCCGGGAGAGCTCAGATCT_CAATGCTATGCAGCAAGACCAG			
VPEB-1012-fw	GATCCTCTAGAGTCGACCTGCAG_CTCGATCATAAGACACTGCG			
VPEB-627-rv	GAGCAGACTCACCTAACAATCGG			
P-JuB-01-fw	CCTCTAGAGTCGACCTGCAG_CGTGACAGCACCGGTCGAAG	2469 pEB17_Δ*pipDEFGH* (pEB134)	Deletion of PSEEN_RS12615-12635 (*pipDEFGH*)	Δpip*DEFGH*-pCEP*pipA* (2470)
PEB-1081-rv	GCACCGGATGCTGGAGCTGCACACCGTGCAACAGCCGCCAGTAC			
PEB-1082-fw	CCAGGCGTACTGGCGGCTGTTGCACGGTGTGCAGCTCCAGCAT			
PEB-1005-rv	GTGGAATTCCCGGGAGAGCTCAGATCT_CCAGTGTTCACATCGAGCTC			
V-JuB-5-fw	AGTCGGTCTGTCGACACAGGAAG			
VPEB-1007-rv	GTTGTAGGCGTACGCCAAG			
PEB-2087-fw	GATCCTCTAGAGTCGACCTGCAG_GCCGAGCCTCGTGGTTATC			

PEB-2082-rv	CAGGTCGCTATTGATCGCCTCGGCGATCCAAGGA GGAAGTGTCGGGCAT	2599 pEB17_Δ*pipABC*. (pEB179)	Deletion of PSEEN_RS12600-12610 (*pipABC*)	Δ*pipABC* (2600)
PEB-2083-fw	GTAGCCTCGATGCCCGACACTTCCTCCTTGGATCG CCGAGGCGATCAATAGC			
VPEB-2085-fw	GATGTTGTCCAGCACCAGG			
VPEB-2086-rv	CGAATCTCTGCCAGTTGCTC			
PEB-2091-fw	CCGTTTAAACATTTAAATCTGCAG_CCAAACTGCTC GGCAATCATCGCC	2602 pCEP*pipD* (pEB180)	Promoter exchange to PSEEN_RS12615 in Δ*pipABC*	Δ*pipABC*-pCEP*pipD* (2603)
PEB-2092-rv	CCGTTTAAACATTTAAATCTGCAG_CCAAACTGCTC GGCAATCATCGCC			
VPEB-2093-rv	CGTTGGCGTCGGTGCTTAG			

Table S1.6. Primers, generated plasmids and strains for heterologous expression of *pipC* and *fabD* in *E. coli* DH10B::*mtaA*.

Name	Sequence	Plasmid	Strain
PEB_1026-fw	CGTTTTTTTGGGCTAACAGGAGGAATTCC_ATGTCTGCATCCCTCGCATTC	pEB114	2341 *E. coli* DH10B::*mtaA*-pACYC_ara_*fabD*
PEB_1027-rv	GTGGCAGCAGCCTAGGTTAATTAAGCTG_GCAAGCGTCTCCAGATTCAG		
PEB_1030-fw	CGTTTTTTTGGGCTAACAGGAGGAATTCC_ATGAGCGTGTTCCAGGAAAC	pEB115	2342 *E. coli* DH10B::*mtaA* pCOLA_ara_*pipC*
PEB_1031-rv	GTGGCAGCAGCCTAGGTTAATTAAGCTG_TCACCGGCCATGAACACC		
PEB_1030-fw	CGTTTTTTTGGGCTAACAGGAGGAATTCC_ATGAGCGTGTTCCAGGAAAC	pEB118	2356 *E. coli* DH10B::*mtaA* pCOLA_ara_*pipCDE*
PEB_1033-rv	GTGGCAGCAGCCTAGGTTAATTAAGCTG_ACCCTGAAACAAGCACCTGC		

Table S1.7. Primers, plasmids and strains generated to introduce point mutations in the catalytic center of PipF and PipG using p*pipF* (pEB140) and p*pipFG* (pEB138) as template.

Name	Sequence	Plasmid	*E. coli* strain	*P. entomophila* strain
P-Ser18-fw	GCGCACCTAGGCGTCGTCGG	pEB146	pEB146-pSEVA621-GM-*pipF* Ser18*-Ala (2559)	Δ*pipF*-pCEP*pipA* + p*pipF* Ser18* (2565)
P-Ser18-rv	GTCGCCCAACAGCAGGAAG			
P-Asp208-fw	GCGCCGTTGCATGGCAACCTC	pEB147	pEB147-pSEVA621-GM-*pipF* Asp208*-Ala (2560)	Δ*pipF*-pCEP*pipA* + p*pipF* Asp208* (2567)
P-Asp208-rv	GTCCACCTCGCAGAACTCG			
P-His211-fw	GCGGGCAACCTCGCGTTTG	pEB148	pEB148-pSEVA621-GM-*pipF* His211*-Ala (2561)	Δ*pipF*-pCEP*pipA* + p*pipF* His211* (2569)
P-His211-rv	CAACGGGTCGTCCACCTCG			
P-Cys47-fw	GCGTTCGCCCAGCACATC	pEB149	pEB149-pSEVA621-GM-*pipFG* Cys47*-Ala (2562)	Δ*pipFG*-pCEP*pipA* + p*pipFG* Cys47* (2571)
P-Cys47-rv	GGACCCGGCGGTGACGAT			
P-His51-fw	GCGATCGGCCGGGCCCTG	pEB150	pEB150-pSEVA621-GM-*pipFG* His51*-Ala (2563)	Δ*pipFG*-pCEP*pipA* + p*pipFG* His51* (2573)
P-His51-rv	CTGGGCGAAACAGGACCC			
P-Thr238-fw	GCGGCCAGCGCCACCGG	pEB151	pEB151-pSEVA621-GM-*pipFG* Thr238*-Ala (2564)	Δ*pipFG*-pCEP*pipA* + p*pipFG* Thr238* (2575)
P-Thr238-rv	GAGGGGAACCGGCGAGAC			

Table S1.8 Primers, plasmids, generated *E. coli* strains and respective complemented deletion strains.

Name	Sequence	Plasmid	*E. coli* strain	Strain
PEB_1048-fw	GTTTAATACTAGAGAAAGAGGGGAAATACTAG_GTGCCCGCGTGGCTGGGC	pEB136 p*pipD*	pSEVA-ara_RS12615 (p*pipD*) (2444)	Δ*pipD*-pCEP*pipA* + p*pipD* (2520)
PEB_1049-rv	GTCGCCAGGGTTTTCCCAGTCACGAC_CGTTCGATGGCCTTCAGTGCC			
PEB_1051-fw	GTTTAATACTAGAGAAAGAGGGGAAATACTAG_ATGGATCAACAGGCACTGAAGG	pEB143 p*pipE*	pSEVA-ara_RS12620 (p*pipE*) (2504)	Δ*pipE*-pCEP*pipA* + p*pipE* (2530)
PEB_1050-rv	GTCGCCAGGGTTTTCCCAGTCACGAC_CTCGATCTCTAGAGCGCTGCATA			
PEB_1066-fw	GTTTAATACTAGAGAAAGAGGGGAAATACTAG_GTGAAGACGCCATTCTCGATTAC	pEB140 p*pipF*	pSEVA-ara_RS12625 (p*pipF*) (2448)	Δ*pipF*-pCEP*pipA* + p*pipF* (2528)
PEB_1067-rv	GTCGCCAGGGTTTTCCCAGTCACGAC_TCATGGGCTCCGCAACGC			
PEB_1068-fw	GTTTAATACTAGAGAAAGAGGGGAAATACTAG_ATGAACCCCTACCAATACCTGC	pEB137 p*pipG*	pSEVA-ara_RS12625 (p*pipG*) (2445)	Δ*pipG*-pCEP*pipA* + p*pipG* (2522)
PEB_1069-rv	GTCGCCAGGGTTTTCCCAGTCACGAC_CAGCAGGAAGCTAGGCGTAG			
PEB_1044-fw	GTTTAATACTAGAGAAAGAGGGGAAATACTAG_ATGCTGGAGCTGCACACC	pEB132 p*pipH*	pSEVA-ara_RS12635 (p*pipH*) (2424)	Δ*pipH*-pCEP*pipA* + p*pipH* (2514)
PEB_1047-rv	GTCGCCAGGGTTTTCCCAGTCACGAC_GTATTGGTAGGGGTTCATGGG			
PEB_1064-fw	GTTTAATACTAGAGAAAGAGGGGAAATACTAG_ATGGCCGGTGAATATGCACAG	pEB144 p*pipDE*	pSEVA-ara_RS12615-RS12620 (p*pipDE*) (2505)	Δ*pipDE*-pCEP*pipA* + p*pipDE* (2532)
PEB_1050-rv	GTCGCCAGGGTTTTCCCAGTCACGAC_CTCGATCTCTAGAGCGCTGCATA			
PEB_1068-fw	GTTTAATACTAGAGAAAGAGGGGAAATACTAG_ATGAACCCCTACCAATACCTGC	pEB138 p*pipFG*	pSEVA-ara_RS12625-RS12630 (p*pipFG*) (2446)	Δ*pipFG*-pCEP*pipA* + p*pipFG* (2524)
PEB_1067-rv	GTCGCCAGGGTTTTCCCAGTCACGAC_TCATGGGCTCCGCAACGC			

PEB_1044-fw	GTTTAATACTAGAGAAAGAGGGGAAATACTAG_ATGCTGGAGCTGCACACC	pEB133 p*pipFGH*	pSEVA-ara_RS12625-RS12635 (p*pipFGH*) (2425)	Δ*pipFGH*-pCEP*pipA* + p*pipFGH* (2516)
PEB_1046-rv	GTCGCCAGGGTTTTCCCAGTCACGAC_GATTCATCCGCGATGCGGTC			
PEB_1083-fw	CCCGGCTTTGGCGTTGCGGAGCCC_ATGAGCGACCTGCTTGCGCGGGAG	pEB141 p*pipDFGH*	pSEVA-ara_RS12625-RS12635+RS12615 (p*pipDFGH*) (2481)	*P. viridiflava*-pCEP*pipA*+ p*pipDFGH* (2488) *P. syringae*-pCEP*pipA*+ p*pipDFGH* (2484)
PEB_1085-rv	CTAGTCGCCAGGGTTTTCCCAGTCACGAC_TCAGTGCCTGTTGATCCATACG			
AR_900-fw	GTCGTGACTGGGAAAACCCT			
PEB-1086-rv	GATTCATCCGCGATGCGGTC			
PEB-1083-fw	GCCCATGAGACCGCATCGCGGATGAATC_AGCGACCTGCTTGCGCGG	pEB145 p*pipDEFGH*	pSEVA-ara_RS12615-12635 (*pipFGH-DE*) (2552)	Δ*pipDEFGH*_pCEP*pipA* + p*pipDEFGH* (2482xpEB145) (2553)
PEB-2031-rv	GTCGCCAGGGTTTTCCCAGTCACGAC_CTCTAGAGCGCTGCATACCCTG			
PEB-1065-fw	GTTTAATACTAGAGAAAGAGGGGAAATACTAG_GTGGACTTCTTCACCCTGGC			
PEB_796-rv	CATCGTTGCTGCTGCGTAAC			
PEB-2050-fw	AGCGGTGCGCGAACAACTG	pEB163 p*pipEpipH*	pSEVA-ara_RS12620+RS12635 (*pipE*+*pipH*) (7164)	Δ*pipDEFGH*_pCEP*pipA* + pEB163 (7168)
PEB-2051-rv	CTATCCGTGCAGGTCGAGGC			
PEB-2052-fw	AGCGACCTGCTTGCGCGG	pEB164 p*pipDEH*	pSEVA-ara_RS12615+12620+12635 (*pipDEH*) (7173)	Δ*pipDEFGH*_pCEP*pipA* + pEB164 (7175)
PEB-2051-rv	CTATCCGTGCAGGTCGAGGC			
PEB-2054-fw	ATGAACCCCTACCAATACCTG	pEB166 p*pipDEFG*	pSEVA-ara_RS12615+12620+12625+12630 (p*pipDEFG*) (7166)	Δ*pipDEFGH* + pEB166 (7170)
PAR-970-rv	CTAGTATTTCCCCTCTTTCTCTAGT			
PEB-2052-fw	AGCGACCTGCTTGCGCGG	pEB170 p*pipDFG*	pSEVA-ara_RS12615+12625+12630 (p*pipDFG*) (7172)	Δ*pipDEFGH*_pCEPpipA+ pEB170 (7178)
PEB-2061-rv	GATTCATCCGCGATGCGGTC			
PEB-2050-fw	AGCGGTGCGCGAACAACTG	pEB171 p*pipEFGH*	pSEVA-ara_RS12635+12630+12625+12620 (p*pipDFGH*) (7179)	Δ*pipDEFGH*_pCEP*pipA* + p*pipEFGH* (7180)
PEB-2061-rv	GATTCATCCGCGATGCGGTC			

Table S1.9. Primers, plasmids, generated *E. coli* strains and promoter exchange strains in *P. syringae* and *P. viridiflava*

Name	Sequence	*E. coli* strain	Purpose	Strain
PEB_1038-fw	TTTGGGCTAACAGGAGGCTAGCATATGCTCGACAGATCTTCCTC	2365 pCEP_LMDDJHCF_09560 (pPH65)	Promoter exchange to LMDDJHCF_09560	(2369) *P. viridiflava*-pCEP*pipA*
PEB_1039-rv	CCGTTTAAACATTTAAATCTGCAGCCTGGTGATGATCACTGAC			
VPEB_1040-rv	CTCGACAACCTGCTGCAATGTG			
PEB_1041-fw	TTTGGGCTAACAGGAGGCTAGCATATGGTCGACAAATCTTCCTC	2366 pCEP_GHKPGMKM_08200 (pPH66)	Promoter exchange to GHKPGMKM_08200	(2367) *P. syringae*-pCEP*pipA*
PEB_1042-rv	CCGTTTAAACATTTAAATCTGCAGCGAGGTGTAGATCACATACG			
VPEB_1043-rv	CTCGCTCAACAGGCGATACAG			

Table S1.10. Primers, plasmids and generated strains for heterologous expression of genes derived from *S. flavogriseus.*

Name	Sequence	Plasmid	Strain
PEB-2068-fw	CGTTTTTTTGGGCTAACAGGAGGAATTCC_ATGCCACACGCCTCACGC	pEB175 pACYC_ara_pgaptmp_006718-CM	*E. coli* DH10B::mtaA-pEB175+pEB176 (EB 7181)
PEB-2063-rv	GTGGCAGCAGCCTAGGTTAATTAAGCTG_GGTCGCGCATCACGCCTC		
PEB-2070-fw	GGCAGGCCGTCGACAGGATGGCCGACGGAGCCGACATCTCCCTGC	pEB176 downstream fragment pCOLA_ara_pgaptmp_006719-KM	*E. coli* DH10B::mtaA-pEB175+pEB176 (EB 7181)
PEB-2065-rv	GTGGCAGCAGCCTAGGTTAATTAAGCTG_GTCGGTGGTGGTCAGCGTG		
PEB-2064-fw	CGTTTTTTTGGGCTAACAGGAGGAATTCC_ATGCGCGACCACCTCCTC	pEB176 upstream fragment pCOLA_ara_pgaptmp_006719-KM	
PEB-2071-rv	CAGTCGGCGCAGGGAGATGTCGGCTCCGTCGGCCATCCTGTCGAC		
PEB-2066-fw	CGTTTTTTTGGGCTAACAGGAGGAATTCC_ATGGAGGAGTACGCACTCCAG	pEB183 pCDF_ara_pgaptmp_006720-spec	*E. coli* DH10B::mtaA pEB175+pEB176+pEB183 (2614)
PEB-2067-rv	GTGGCAGCAGCCTAGGTTAATTAAGCTG_CGAGGTCAGGTCGTCGAACC		
PEB-2094-fw	CGTTTTTTTGGGCTAACAGGAGGAATTCC_ATGCTGGAGCTGCACACC	pEB182 pCDF_ara-*pipDEFGH*-spec	*E. coli* DH10B::mtaA pEB182 (EB 7194)

PEB-2095-rv	GTGGCAGCAGCCTAGGTTAATTAAGCTG_CTCTAGAGCGCTGCATACCCTGGTGGCAGCAGCCTAGGTTAATTAAGCTG_CTCTAGAGCGCTGCATACCCTG		*E. coli* DH10B::mtaA pEB175+pEB176+pEB182 (EB 7199)
PEB-2072-fw	GTTTAATACTAGAGAAAGAGGGGAAATACTAG_ATGCGCGACCACCTCCTC	pEB177 pSEVA_ara_pgaptmp_006719-GM	*E. coli* ST18_pEB177 (2597)
PEB-2073-rv	GTCGCCAGGGTTTTCCCAGTCACGAC_GTCGGTGGTGGTCAGCGTG		
PEB-2072-fw	GTTTAATACTAGAGAAAGAGGGGAAATACTAG_ATGCGCGACCACCTCCTC	pEB178 upstream fragment pSEVA_ara_pgaptmp_006719-006720-KM	*E. coli* ST18_pEB178 (2598)
PEB-2075-rv	GCGATGCCTGCGTGTGCGAGGGCCTGCACGCAGGCCATCAGCAG		
PEB-2074-fw	CAGCGGCACCTGCTGATGGCCTGCGTGCAGGCCCTCGCACACGCA	pEB178 downstream fragment pSEVA_ara_pgaptmp_006719-006720-KM	
oPH98-r	GTCGCCAGGGTTTTCCCAGTCACGAC_CGAGGTCAGGTCGTCGAACC		
PEB-2062-fw*	GTTTAATACTAGAGAAAGAGGGGAAATACTAG__GTGCCACACGCCTCACGC	pEB181 pSEVA_ara_pgaptmp_006718+006719-GM first fragment	*E. coli* ST18+pEB181 (2605)
PEB-2098-rv	GTGGCGAACAGCGGAGGGCTCTGCACCACCTCGTCGTGGAGCACCAC		
PEB-2099-fw	GTCTGTGTGGTGCTCCACGACGAGGTGGTGCAGAGCCCTCCGCTG	pEB181 pSEVA_ara_pgaptmp_006718+006719-GM second fragment	
PEB-2075-rv	GCGATGCCTGCGTGTGCGAGGGCCTGCACGCAGGCCATCAGCAG		
PEB-2074-fw	CAGCGGCACCTGCTGATGGCCTGCGTGCAGGCCCTCGCACACGCA	pEB181 pSEVA_ara_pgaptmp_006718+006719-GM third fragment	
PEB-2073-rv	GTCGCCAGGGTTTTCCCAGTCACGAC_GTCGGTGGTGGTCAGCGTG		

Table S1.12. DNA sequences of *S. flavogriseus* (Sf) *pipBCE*.

>pgaptmp_006718 (*Sf-pipB*)
GTGCCACACGCCTCACGCCATGTCACTCCCCCCTCGCCGACGGCTCCGGAGCCGGTCGGGCCACGCGATTCCGGTGTC
CCGGCGGCTGTCAGGCCGGCCTCGTTCGCCCAGAAGCGCCTCTGGTTCCTGGCCCAGCTCCCCAGCGGGAACGCCGCG
TACAACGAACCGCTCGCCTTCCGGCTCGACGGGCCGCTCGACCGCGACGCCCTGGCCCACTGCCTCGACATGCTGACAG
AGCGTCATGAAGCGCTCCGGACACGCCTGGTGGAGAGCGAGGGGGAGGTGTGGCAGCACATCGACCCGCCCGGCGCC
GGTTTCTCCCTGGCTCTCGTCGACCTCACGGACAGCGCTGACCCCGAGGCGCGCCTCGAAGCGCTCAGGGCGGAGGAG
GCCGGCACCCCCTTCGACCTTGCCGTCGGACCGCTGGGACGCGGAACCCTGGTCACTCTGGGGGAGGGGAGCCAGATC
CTCCTGCTGACGTTCCATCACACCGTCTACGACGGACATTCGATGGGCGTCATGATGCGAGAGCTCGGCCGCCTCTACGC
GGCCCGGACATCCGGTGACACCGACCCGCTGCCTCCGCTCGACGTGCAGTACGCCGACCACGCGCAGGTTCAGCACGA
CGCCGTCACCGACGGTGCTCTGTCCGCCCAGGAGTCCTACTGGCAAGGCGTGCTCGCGGGCGCCCCCGCCGTGCTGGA
GCTGCCCGCCGACCGCACGCGCCCCGCCGAGCAGGCCTACGAGGGCGGCCGGGTCGAGTTCGCGGTCGACGCGGCGA
CCACCGACGCGCTGCGAGCCATGGCACGCAGGCACGGCGCCACGCTCTTCGTGACCGTCCTGACCGGGTGGGCGATCC
TGCTCTCCCGGCTCTCCGGACGCGAAGACATCGTTGTCGGCAGCCCGGTCGCCAATCGCCGAGGGCCCGCCGCCGCGG
ACCTGATCGGCTTCCTCGTCAACTCGCTGCCCCTACGGGTCGACCTGTCCGGCTCACCCACAGCGGCCGAGGCGCTGAC
GCGCACCCGCGCCGTCGTACGCGAGGCGCTCAGGAACCAGGACCTGCCTTTCGAGCGCATGGTGGAACTGGTCAACCC
GCCTCGCAGCGCCGCCCACACGCCCCTCTTCCAGACGATGTTCGCCTGGCAGCCGGACCGGCACGACCTCCTCGACCTG
CCCGGAGTGCAGGTGCGGCCCCTTCCGATCGGCCATGCTCCCGCGCGTTTCGAACTGTCGCTGGTGGCGGTCGACGCC
GGGGACCGCGTCACCGGCCACCTGGACTACGCGACCTCCCTCTACGACCGGGCGACGGCGCAGCGCTGGGCCCGACAC
CTCGAACACCTGCTCCGGGACATGGCCCGCGCGCCGGAGCGGACCATCGGGGCGCTGGAGCTGATGGAAGACGAGGAG
CAGTTGCGCCTGCTGCGCGACTGGGATGCCACCGGCAGCGTCCTGGAGCTCTCACCGCACGCCCCCGAGGGCTCGGTG
ACGCCGAAGTCCGTCCTGGCGGCCACCGGGCTCGTGGAGCTGTTCGAGGATCAGGTGCGCGCGCGGCCGCACCACACC
GCCGTCGTGGGTCGGGACGGTACCTTGGACTACGCGGCCCTCGACCGTCGCGCCGACCGCCTCGCCCACGCTCTCACC
GCCCGCTCCGTGGGGCCCGGCGACGTGGTGGGCCTGCATGTCAGCCGCACCACCGCACTGGCCGTCGGCATCCTCGGC
ATCCTCAAGGCCGGTGCCGCCTACCTCCCTCTCGACCCCGGGCAGCCTTACGAGCGGCTCGCCGCCATGATCGAGGACG
CGGCCTGCCCGGTGGTGCTGAGCGACGGTGTCGGCGACGGACGCCCGGGCGGGTGGCTGGACCTGGCGGCCCTCGAG
GCCGAGGGAACCGGCGAGCGGGCGCCGGGCAGCATCGGCCACACCCCGGACCGGCTCGCCTACGTGATCTTCACCTCC
GGTTCCACCGGACGGCCCAAGGGCGTCGCGGTGGAGCACCGCAGCGTGCTCAACCTGTTCGCCAACTGGCAGTCCCGC
ATGGGGTCCGGCCCCGGCGAGCCCGGCTCCGCCTGGTCGAGCATCGGGTTCGACGCCTCGGTCCACGAACTGCTGCTC
CCACTGATCACCGGGGGTACGCTCCACATCGTCCCCGAGGAACTGCGCGGCGACCCGGCCTCCCTCATGACGTGGATGG
GCGAGCACGGCATCACCCAGGCCTTCCTGCCGCCCGCCTACGTGAAGTGGATCGACGAGGACCCCGCGCTGCGGCTGG
CCGGACTCCGGCTGCGTACCCTGCTCACCGGCGTCGAGTCGCTCACCGAGGCCGCCCTGCACCGCCTGACCCGGCACC
TGCCCGGCCTGCGGATCTGCTTCGGCTACGGACCCACCGAGACGACGCTCTACAGCACCGCCTACTACGAGCCCGGCCC
GCTGGAGCGGCAGTGCCCCGTGGGGCGCCCGCTCGGCAACACCCGTCTGTACGTGCTCGACGCGGACCTGCGACCGGT
CCCGCCCGGCGTGGTCGGCGAGGTCTACCTCGGCGGTGACTGCCTCGCCCGGGGCTACCTCAACCGCCCCGACCTCAC
CGAGGAACGCTTCCTGCCCGACCCGTTCCTGCCCGGCGAACGCGTCTACCGCACCGGTGACCTCGCCCGCCGCCTCCC
CGACGGCCAGGCCCATTACGCCGGCCGCGCCGACGACCAGGTGAAGCTGCGCGGCTTCCGGATCGAGCCGGCCGAGAT
CGAGGCCGCGCTGGGCACACTGCCCGGGGTCCGGGAAGCGGTCGTCCTGGTCGACCCGGACACGGAGGGGCAGCCCC
GGCTCGTGGGCTGTGTCGGGCGGGGCGAGGCGGTACAGCGCACCACCCAGGACTGGCGCACCGAGCTCTCCCGCCTG
CTGCCCGACTACATGGTCCCTGCCGTCTTCGTGGACCTGCCGGCCCTCCCGCTCAACCGCAGCGGCAAACTCGACCGGG
AGGCGCTGCTCGAACTCGCACGCTCCCGGACGTCCCACGGGGTCAACGCGGCCAGCCCGCGCGACCACATAGAGCACG
AGCTGTACCGGATATGGGGAGAGATCCTGCTCGCCCCGGACATAGGCATCAGCGACGACTTCTTCGAGAGGGGCGGCAC
CTCCCTGTCCGCGATCAAGATGGCCCACCGTGTCTCCGAAACCTTCGGCCGCTCCATACCGGTGGCCGAGATCATCCTCC
GGCCGACCATCGAGGCCCTCGGCGCGGTACTGCGCGAGGGAGGGACCCCCGGCGCCACGGGCAACGTCATCGAGTTCC
GGGGCGGCACGGGCGCGCGGGTGATCTGCGTACACCCCGCCGGAGGCACGGCCTTCTGCTATCTGCCGCTGGCCGGAC
TGCTCCCCGACTCGGTGGCCCTGCACGGCATCCAGTCCCCGGGGCTGAACGCGGGGGAGAGCCACATGCCGAACGTCG
AGGCGATGGCCGAGACGTACCTACGGCTCCTGGAGCCGCTGGGTCCGGGGCCGATCGTGCTGACCGGCCTGTCCTACG
GCGGGCTCGTCGCCCACGAGATGGGACGCCGACTCGCCCTCGGCGGCCGCACCGACGTCAGCGTCGTCCTGCTCGACA
CCCAGGCCACCGACGACCCGGCCGCCCGCGAAGCCGTCGCCCCCGTGGACATGGCCGAGTTCCGCGACAAGCTGGTGC
GGTTCAACGGCATGTACCCCGGTATCGAGGACGCCCAGGTGGAGCGGTACTTCCAGATCTACAACCACAACCGGATGAC
CGCCCGGGACCACGTCCCCGCCACCTCCGGTGCACGCGTCGTCCTCGCCCAGGCGGTGCCGGGGCTGGAGGACACCC
CCTTCCACGCGGAGGTGCGCGACTTCTGGCGTCGCCGTTGCCCCGGGCTCCGGATCGAGACCATGGACTGCGACCACTG
GGAGATACTGGAAAGCGCCGAGGTCCCACAGGTGGCGGACATCATCGCCGCCGAGCTCGCGCACCACACGATCCTGCC
GGTCACCGGTGACGCCCTCCCGGCCCGGGAGGCGTGA

>pgaptmp_006719 (*Sf-pipC*)
ATGCGCGACCACCTCCTCCCGTCGGCCGGTGCCCCGGCTCTCCCTTCGCTCCCGCCGAACCCGAGCCTGGCCGAGGCC
GTGCTCGCCCGCGCCCGGCACACTCCCGGCGCACCTGCCGTCGAGGACGGAGCGGTCGTCGTCGACTACGCCGGACTC
GACCGCCTCAGCGGCCGGGTGGCGTCAGCCCTGCGTGCCGCCGGTGTACGCCCGGGGCAGGCGGTCGCCGTGTGCCT
CCCCCGGTCGTGGCAGCTCGTCTGCGTCATGCTCGGCATCCGCCGCGCCGGGGCGACCGTCGTGCCGCTGGACCGTCT
CAGCCCCGACCGGCGCCGCCGGCACGTACTCGAGGACTCGGGTGCCGTGGCGGCCGTCCATCGCGCTGCGGACGGGA
TCGACCTGCCGGCCTCCCTCCCGGCGCTCGACGCGGACCTGCTGATGGCGGGAGAGGACGAGCCCGCCGACGCGTCGT
CGCCCGCCGAGCCGCGGCAGGACCCCGCCTCCGAGGCGGCCTTCGTCTTCTACACGTCGGGGACCACGGGCCTGCCCA
AGGGCGTCGAGGTCACCGACGCGGGCGTACTCAGGCTGGCCGGAGCGCGGTACGTGACGCAAGGGCGGAGCAGCCGC

TTCGGCTGCCTGTCCAACCCCGCCTTCGACGCACTCAGCTTCGAGGTCTGGACACCGCTCCTGACCGGCGGCGTCTGTG
TGGTGCTCCACGACGAGGTGGTGCAGAGCCCTCCGCTGTTCGCCACGGCCCTCCGCGACCTGGGCATCGACACGATGTT
CATCACCGTGTCCCTGTTCAACACCGTCGTGGCGGCGGTGCCCGACTGCTTCACCACCGTCGGACGGGTACTGATCGGG
GGCGAACAGCTGAACGCCCACGTCGTACGGCGCTGGTACGAGAACAACCCGGGCAGCGGCACGGTCCTGTTCAACGCG
TACGGACCCACCGAGGCCACCACGTTCGCCCTGTGCCATCCCGTGCCCAGGGAGTTCACCGGTGACGCGGTCCCCATCG
GCACCGCGCTGCCCGGCACCGGACTCCTCCTGCGCACCTCCGACGGGCGCATCGCCGAACCCGGCGAGACGGCCGAAC
TCCTGCTCACGGGCGAGGCGCTGGCCCTCGGCTACCGCAACCTTCCCGAGGAGACCGAGCGGCGCTTCGTCCTCCTGC
CACGGGAGGACGGCGGCGAGGAACGCTGGTACCGCACGGGCGACCTCGTACGGGCGGACGACGAGGGCCGCCTCACC
CACCTCGGCCGCGCCGACCGCCAGGTCAAGGTGCGTGGTTTCCGCATCGAGCCGGGGGAGGTGGAACGCAACCTGCTG
GCCCATCCCGCCGTGTGCCAGGCCCACGTGTGCACCCGGCGCGAGGACGCCGACCAGCACGAACTGCTGGCGTTCCTC
GTGCTGGAAGGCGGCGTACCGGAAGACGAACTCTCCTACGAGGCCTACGAACGGCACCTCGCCGACACCCTCCCGGCGT
ACATGCGTCCCCACCGCACATACCTGGTCGAGCGGATCCCCCTCAGCGCCAACGGGAAGACGGACGAGGTCGCCCTGCT
CGGCACCGCCGGCACCCAGGAGCCCTGGCGTGGCGGACACCGTGACGACCAGGTGGCGACCGCCGCCCAGCGGGAGA
TCCTCGACCTCGCCGCCGACGTGCTCGGGATCGCAGGGCTGCGTCCCTCCGACCGGCTGACAGCGAGCGGCGGCGACT
CGCTCAAGGCGCTGCGCCTGCGGTTCGACATCCAGCGTCGGTTCGGTGTGGACCTTCCCCAGGACCTCGTGCTGCGGGC
CGACTTCGCCGCCATCGCGGACGCCGTGCACGCCCCGGACGCCGCCGGTGACATCCACCCGCCCGTACCGACCGCCGC
CCCGGGCCCCACCTCGCCCGCCACCAGCGAGCAGGAGCGCCTGTGGCTCCAGCACGAACGCGACCCCGAGGACGCCG
CCTACGACGTCCCGCTCGCCTTCGAGGTGCGCGGACCGTACGCCTCCCCGCGCTCCGGCGGGCGGTGCGGACGGTG
GTGGAGCGCCACACATCGCTGCGCACCCGCCTGGTCCCCACCGCGCAGGGACTGCTGCAGGAGGTGGAAGCCCCCTAC
GACCCGTGGCAGCCGTGCGAGGCCGACGCCGACGAGTCGTGGCAGGACTCGGCCTCCCGCTTTTTCGCCCACCGCTTC
GACCTGCGCTCCCCGTACATGTGCCGTGCCACCTGGCTGCCGTCCGCCGAGCCGGACACGGACGGCCCGGAGCGCGGC
GACGGCGCGGCTTCAGGCGCCGCCGACGGGGGAACCCTCCTGCTGCACCTGCACCACATCGCCGTGGACGGCTGCTCG
CTCGGCATCATCCTCGGCGACCTCACCGAGGCGTACGGAGCGGCGTGCGAGGGCGCGGAATCCCCGGCCGCCCCGGG
CCCGGCCACGACCGTCGCGCAGTTCGCGCACTGGCAACGGGCCTGGCGCGCGAGCCCCGCCTACGACGAACGGCGCA
CCCGTCTGCGCGCGTACTACGAGCAGGACGCCGAGACCGCGCCCGCCCCCGCGGTGTCCCGCGCACCCTCCAGGCCCT
CCGCCCGCCTGCTGCGCACCACCCTGGACACGGTCCGGCGCGGCGCGCTCGACCGGCTCGCCGCCGAGCAGGGCCGC
ACCCGCTTCCAACTACTGCTGTCCGCATACGTGTGGAGCCTCTACGGCGTGACCGGCCAGACCCGGCCACTCGTGGCCG
CACCGGTGGCGAACCGGCCCCGGCCCGAGTTCGCGGACACGGTCGGCATGCTGGCGAACACCGTGCTGCTGCCGCTGG
ACATGGAGCCCGAGGCCCCGCTGCGCCTCCTGCTGGACCGCCACGCGGCGACGTCCGCGGAGGTCCTGCGAGGACAG
GAGGTGCTGCTCGCCGACGTGGTGGCGGACCGGCCGGCCGGCTCCCGGGGCGAACTCTTCGACTACCTCTTCGTCCAG
GAGAACACCGACTTCTCGGCCCTGCGGCTTCCCGGCTGCACCGTCGGCCCGCTCTGGCCGGCCCCGCTCGGAGCCAAG
TGCAGGATCACCCTCTCTGTCGTCGAGCACGCCTCCGGACTCGACTGCCTGTGGGAGTACCGCGAGGACGTCGACGAGA
ATGAGGTACGGGCCGCCGCCCGCCTCCTCGGGCAGGCCGTCGACAGGATGGCCGACGGAGCCGACATCTCCCTGCGCC
GACTGGTGGCACCGTACCGCCGCACGCTCCCCGAGCACGGCCGAGGTCCCGCGTCCGAACCGGAGTTCGCCACCGTGG
CCGAGGGTTTCGAGCGCCAGGCGCGCCGTACCCCTGACGCCCCTGCGGTCGCGACGGCTGACACCACCCTCAGCTACG
CCGAACTCGACGCACGGGCAGCCGTGCTGGCGGAGGACCTGCGCCCCTATCTGCCCGCCGATCCGGCAGCACCGGCGG
CCGTGGCCCTCTACCTGGAGCCCTCGGTCGAGCACGTGGTGTCGCTCCTTGCCGCCGCCCGGCTCAACCTGACCGCCGT
GCCGCTGGACCCGTCGTACCCGCCGTCCCTGCTGCGCCAGGTTCTCGCCCAGGCCGCGCCGGTGTGCGTCCTGGTGCC
ACCGGACCGTGCCGACGCGCTCGACGCCCTCGCACCCGCGGACCTGCCCCGCCACCCCCTGGTGTGCCCGGCGGACCC
CCCTGCCGCGAAGCCCTCGCCAGGACACCGGGCGCTGCGCCCCCTGTACACCCTGTTCACCTCGGGCTCCACCGGGAC
ACCCAAGGGCGTGCAGGTGCCCGACCGTACACTGTGCAACCTCCTGACCTGGCAGCGCGAACACGGCGGACTGACCGCT
CCCGCCGTCACCCAGCAGTTCTCCATGCTGTCCTTCGACGTCTCCTTCCAGGAGATCTTCACCACCCTGTGCTCCGGGGG
CCTGCTCCGCCTGGTCGACCCCGCCTGGCGGCAGGACATGCCCACCCTCCTGGACCGGTTGGAGAGCGCGGGCGTCGA
GCGGCTCTTCCTCCCCTACGTCGCCCTGCAACTCCTCGCGGAGCACGGAGTGCGGACCGGCCGCCATCCTTCCCGGCTA
CGGGATGTCATCACCGCGGGCGAGCAGTTGGTGTGTACGGACGCGATCCGCCGATGGTTCGCCGCGATGCCCTCCGCG
CGGCTCTTCAACCACTACGGCCCCACCGAGACACACGTGGTCAGCGGCCTCCGACTGGACGGCGACCCCGCCCGATGG
CCACTGCGGCCGGCGATCGGACGGCCCGTCTCGGGCGCCCTGCTCCGCGTGGTGGACGAGGACGGCCACGCCGTTCC
GCCCGGAGCGACCGGCCACCTCTGGCTCGGCGGCCCCATGGCCGGGCGCTGCTACCTGGGCGACACCGAACTCAACCG
CACCCGCTTCGTGGACGACCCCGAGGCGGGCACCTTCTACCGCAGCGGCGACCTCGCCCGCTTCGACCGCCAGGGCCT
GCTGCACTACGCGGGACGGACCGACACCCAGGTCAAGATCAGTGGACACCGGCTGGAACTGGGCGCCGTCGAAGCCGC
GCTGCTCCAGTACCCCGGGGTGACCAACGCGGTCGTCACAGAGACGCACGGCTCCCTCGTGGCCTGCCTCCAGGCCGA
CCAGGACCCCGACGCCGACGCGCTCGACCGCCACCTGAGCGCGATGCTCCCGTCCCACGTCCGCATCAGCCGCTTCCG
TCGCCTGCCCCGGCTGCCGCGCACGCCCAGTGGAAAAGTGGACCGCCAGGCGGCCCTGTCCGCCTCCGGGCACGACCT
GCGCCCCGGCGGGGGCGCCGCCCCTGTGCTCACCGAGATGGAACAACGGCTCACCGACCTGTTCCGGTCGGTGACCGG
CCGGAGCCCCGGTGCGCGGCAGCGGTTCTTCGACGCGGGAGCCACCAGCCTGGACCTCATGCGCTTCCAACTGCGCTG
CGCGGGCGAGGGCGATCTGCACTTCGGCGTTCCGGAGCTGTTCGAGCACGTCACGATCCACTCCCTGGCGCGCCTCATC
GAGGAGAGAGCCGCCCAGGCGGCCGGCGCCGGGACACCCCCCGCAGCCTCCGCACCCGCCGCCTCCGGCCCGGACCT
CCCCGAGGCCGCCCCGGACACCGGCCGCACACCTCCCGCCGGGGGCAACGAACCGGTCGCCGTCATCGGTATGGCCGT
CCGCCTGCCCGGCGCCGCCGACCTGGCCGCGTTCTGGGACCTGGTCACCTCCGGCGGACGCGGCATCGAGGACTTCCC
CGCCGCCGACGGACTGGTCGGGGCCCGCAGCCAGATGGACGGGCTCCTGGCCTTCGACCCGGGCCACTTCGGCATCAG
TCCGCACGAGGCCCGGCTCATGGACCCGCAACAGCGGCACCTGCTGATGGCCTGCGTGCAGGCCCTCGCACACGCAGG
CATCGCCGACGCCGGAGCCGGGCGGGTCGGCCTCGTCGCCGCGTGCGGCGAGAACACCTACTTCCAGAGCCTGCTGAG
GGAGGCCGATCCCGCGACACTGCCCGACTCCTTCCGGCTCGCCCTGCACCACGAGAAGGACTTCCTGTCCACCCGGGCC
GCGTACCACCTCGGCCTGCGGGGGCCGGCGCTCACCGTCCAGTCGGCCTGCTCCAGCTCCCTCGTCGGCGTCCACCTC
GCCGCCGGGCTGCTGCGACAGGACGACGCCGACGTGATGCTCGTCGGCGGCGTCCTCGTGGACCGTGAACTGACGGAC
GGGTACATCCACCGGCCGAACCACATCTTCTCCCCGGACGGCCACTGCCGGCCCTTCAGCGCGGACGCCGCCGGCACC

GTGGGCGCCAGCGGTGTGGGCGTCGTCGTCCTCAAGCCGCTCGCCGCGGCGCGCCGCGACGGCGACACCGTCTACGC
GGTGATCACAGGCTCCGCCGTCAACAACGACGGTGCCGACAAGATGAGTTACAGCGCTCCCTCGTTGGCCGGACAGCGG
GCCGCAGTGAGCACCGCCCTGCGCCGCAGCGGTCGTACGGCCGCCGAGGTCGGCTACGTGGAGGCCCACGGAACCGG
AACCCGGCTGGGCGACCCGATCGAAGCCGCCGCCCTACGCGCCGGTTACGAGCTGGCCGACGACGCAGGTCTCGCCCT
GTCCTCCGTCAAGAGCCAGATCGGGCACCTGGGCGCGGCCGCGGGTGTGGTGGGGCTGGTGCGGGCGGTGCTCGCCG
TCCACCACGGCACCATCCCGCCGACCGTCGACTTCGACCGTCTCAACCCGGCCGTCGACGCGGGTCCGTTCCGGATACC
GGTGACCGCGCAGCCCTGGCCCTCCACGGCGCCACGCGTCGCGGGCGTCAGCAGCTTCGGCATCGGCGGTACCAACGC
CCATCTGCTGGTGGAGAGTCCGGACGGCACGGACACGGCACCGCGGCCTCGCGACCAGCCTCTCCGGCCACCGACGCC
CTGCCTCGTCCTGTCCGGCGACTCACCCGCCGCCCTGATCGAGGACGCCCGCCGGATCGCCGCCCACCTCAGCGCCGG
CCCTGGCGACTACGCACAGGTGTTGCGCCACCTCCAGGCGGGCCGTCCCGTACGGGCGTACCGGGCCGCCGTCATGTG
CGAGGACCCCGAATCGGCCGTCGCGTGGCTGCGCGCACTCGTACCGCCGGCCGACCCGGTGTCACCGAGCGGCGTTCC
GGCCTCGGGCATCGACGCGCTGGAGCCGGCCACGGCCTGGACCGCCGGCCACACCGTCGACTGGACGCCCGGTCCGG
CCTCCGCCCCCTGGGACTTCCCGCCGCCCGCGTTCGACCTGGCGGACTACGACTTCCCGCGCGCGGGACCGACGGCAC
GGACATCGGCGGACGTCCCCGCTCCGCGCACACCGACACCGGTGCCGGGCCGGACCCCGGACCCGCAGCGGCTCCCG
GCCGACCGGTGGACGCACCAGCCGCAGTGGACCCGGCTCCGCAGGGCACGGACCGACGTGTCGGCACCGACACGGAC
CGCCGTGATCGTCGCCGACGAGCGGACCCACCCGACGGCATGGGCGGAACTGGAGCGGCGGTACACGCGGGTGGTGT
GGCTGCTTCCGGGCAAGGACCTCGTACGGATCGACGACGACCGCTACGAGGCGGACCCCTCGGACACCGGGCACCTGG
CGGCCGTACTGCTCGAGGCGGGCGCACCCGGCCCGGCGGGCACCGCGGTCGACTGGCTGCACGCACTGCCCCTCGCC
ATCGACGGGGACATCGGCGAGGAGTCGCTCGACACCGCCCGGTGGGCCTGCCTCGACACCGTCGCCGCCCTGTCCCGG
GCCGTCGCCGACGTCGTGCGGACACACGGCCGCGACCGGCCCCGCGTGTGGTTGCTGTCCTGCGGGGCCCAGCCGGT
CACCGGTCCGGTGCGGCGGCCCGAGGCGGGTCTGCTGACGGCGGCGACGCAGGTGCCCCGACAGGAGCTCGGCACGG
AGATGAACTGGATCGACCTGCCCGGCCCCGACCCCGCCCCGTGGGCCGCGTTCCTGCCCGACCTCCTTCTCGACGCCCC
GGCGGAGACGGCCGCCGTCGCTCTGCGCGACTCCTTCTGGTGGCACCGCGGCGTCCACCCCGTGGCGGCCCCGGCGA
CGCCCCGGAGCGACACGGAACGCGGTCGCGAAACAGGGGCACGGAGCATCGGCACCCACCTGGTCCTCGGCGGCACC
GGGGGCATCGGCGCCGTCCTGGCCGCCCGGATCCTGCGGCACCCGGAGAACCGTGTCGTCCTGCTGTCCCGCCACGCC
CTGCTCCCTCCGGCCCTGGAACCATCGCGAGACCGGATCACCCTGGTGCGCGCGGACCTCGCCCTGGAGGAGCCCGCC
GACCTCGCGGACCGCCTGGCACCCCACCTGGGGGGAGGCCTGGCCGGCATCGTGCATGCGGCCGGAACGGCCGCCGG
TGGGCTGCTCGCGCTGCGCGACGCGGAAACCGCCCGCCGGGGCACGGAGGCCAAACTCCGTGGCGCCCTGCTCATGGA
GCGGCTCATCGCATCGCACCACCCCGGCCACGCCGTCTACTGCTCGTCCATGGCCTCCCTGTTCGGCGGCGTCGGACAG
TTCGACTACGCCGCGGCCAACGGCTGCCTGGACGCGTTCGCCCAGTACGGGGGCGACGACGGCACGAGCCGAACGGTC
CGCATGGGCATCGGCTGGGACGTCTGGCGCGAGGCCGGTATGGCGCTGAGCGCGCTCGGCCACGACGGACAGCACCAA
AGACACCTGCTGACCGGCCTGACATCCGACGAGGGTGCCGCCGTGTTCGATCAGGCCATGCGGCTGCAACTCCCGCACC
TCATGGTCAACACCACCGACCTCGCCCAGGCACGGAGCTTCTACGAACGCCCCGCGACCACCTCCGAGGAGACGCCCCA
CCCTCCCACCGAAGCGGCACGGCCGACGCCCGGCCACGACCGCGTCGCGCAACTCGTCGACACCGTGGCACCGCTGCT
CGGGGTGGACGACGTGGCGACCGACGCGTCGCTGTACGACCTCGGCGCCGACTCCCTGATGCTGCTGGAACTGGCGGA
CCGGATAGAGCAGCGCTTCGGCGTGGACCTCGACCTCTCCCGTTTCAGCCACCGGGTCAGCGTGGCGGAGATCTCCGGC
CTCATCGACGCCGCCGCACGCGACGCACGTCCCACCACCTCGGATGCTCCGGTGCAGGTCGAGGTGTGGCAGCGGGGC
GTCGACAGCTCGGACGTGCTCTGTCTCGTCCATCCGGTCGGCGGTGACATCCAGGCCTACCGCCCGCTCGTGTCCGCGC
TGGGGGACCGGCTCACCGTATGCCTCATCGCGGACCCGGCACTGCGCACCGCCCCGGACCGGCCCGCCGAGCCCCTCG
CCGACCGTGCCGCACGCTACCTGGAGGCGGTCCGGGCCGAACAGGCCGGATCCGGTGGCCGGCTTCTCCTGGCCGGCT
GGTCCTTCGGCGCCTGGATGGCCCTGTCGATGGCCGCTCTCGCGGAGGAGCACGACCTGCCTGCCGCCGGCCTGTACC
TGCTGGACCCGCCGCCGCCCGGGGCGGGCGCGGCGGTCGCGGCCTACGACGACCGGCAGGTGGACGCCGTCTTCGTC
CGCGAACTGGGCGGCAACCGCGCGGGCGGGCTGAGCGGGAGCGGACGTGAATACGCCGAACAGCTGGCCCGGTGCTG
CCGTGCCAACCTGTCGGCCATGGCCGGCCACCGGCTGCCACGCCTGAGCCGCACCCCGAGCTCCCTGTGGATCGCGGA
ACGTACCGTCCCCGACGTGCCGTCCCTCGCGCCGGAGCCGGTGCCCACCGAGGAGTGGGAGGCCCATCTCCCGTCCCC
GGTCCGCGCCCACCGAATCGACGCGGACCACTACACCCTCGTCGCCGCACCCCATGTGGAGTCCATAGCCGCCGTCATC
CTGCAGGACGCGGCACCGCGCGGAGAGGCCGGACACGGGCACCGGACCGACACCGACGAGGGACTCCCGGCCCCACG
CTGA

>pgaptmp_006720 (*Sf-pipE*)

ATGGAGGAGTACGCACTCCAGGCGGTCGAACGCCTGACGACCCGTCCGGCCGAACCGTACGAGACGATCTCCGTCACCC
CCGTCACCCCGGTACTGGGTGCCGAGGTGACGGGCCTCGACCTGTCACAGGAACTGACGGACCGGCAGGAAAAGGAAC
TCGTAAACGCGTTCCTCGCCCACCATGTGCTGGTCTTCCGCGACCAGGACATCACCCCCGAGCAGCACAAGCGGTTCGC
CGCCCGCTTCGGTGAGCTGCACCCTGTGGCGCTCGCCGCCGAGGGTTCGGACCCGCACATCCTGGAGATCAGGGCCAC
GAAGGAGTCCCGGGCCATCGCGGGCAACGGCTGGCACGCCGACGGCACTGCCGACACCGACCCCTCGCTCGGCTCCAT
GCTGTACATCACGGAGATCCCCGAGGGGGGCAGCGGTGGAGACACCCTGTTCGCCAACATGCACCTGGCCTACGAACTC
CTCTCCCCGGCCATGAGGTCCTTCCTGGACGGGCTCAGCGCCCTGCACGACGGCGCCCTGCCCTGGGCCACCGCCGGA
CAGACCCCGCCGGCGGACTACGACATCCCCCGTACCGAGCATCCCGTGGTCGTCCTGCACCCGGAGACCGGACGCAAG
CTGCTCTTCGTCAACGGCCCCTACACCTCCCACATCACCCAGCTCGCGAGGCCGGAGAGCGATGCGCTCCTTCAGATGCT
CTACGCCCACATAGCCCGCACACCTCTGCTCCAGTGCCGGATCCGCTGGCAGCCGCGCACCCTGGTCTTCTGGGACAAC
CGCTGCGTACAGCACCACGCGGTCTGGGACTACTTCCCCCACGGCCGTTACGGCCAGCGGGTGGCTATCAACGGAACTC
GTCCCCAGCGGGGTTCGACGACCTGA

Supplementary Figures S1

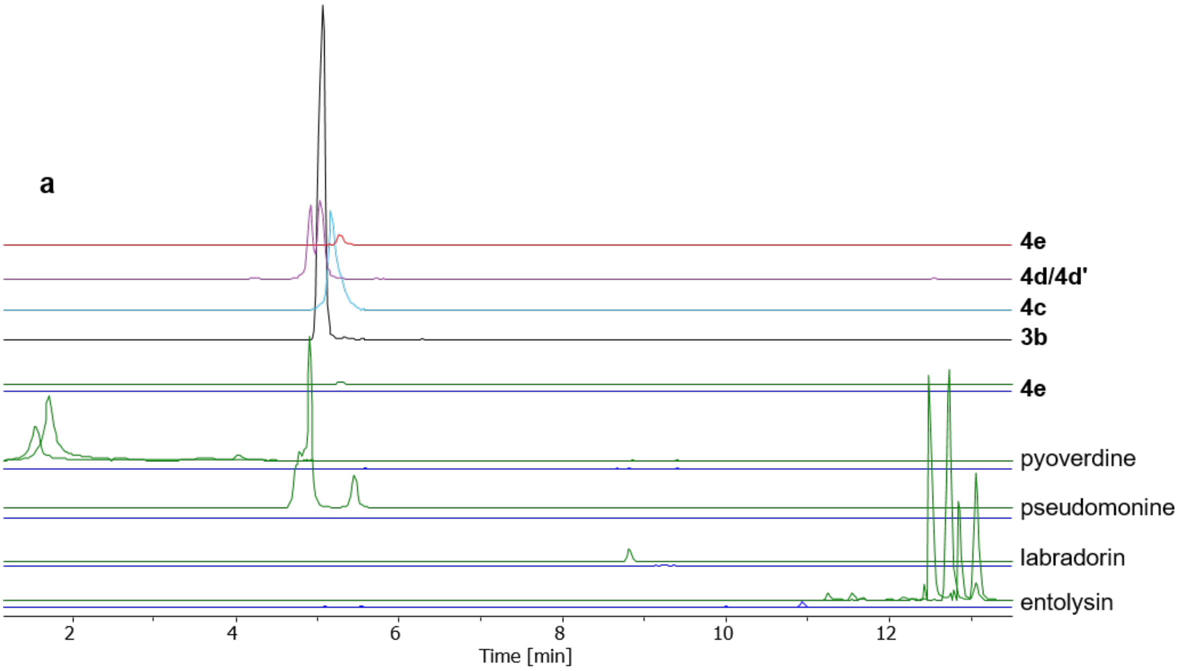

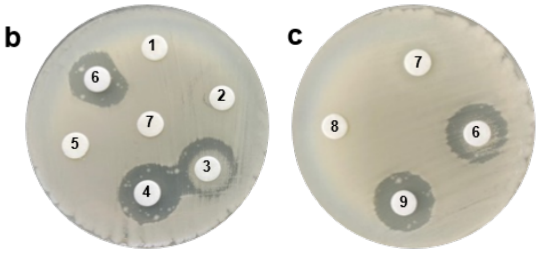

disc	862	BCS	7001	849
1	20	1		
2	2	1		
3	0.2	1		
4	0.02	1		
5	20			
6		1		
7	MeOH			
8		1	20	
9		1		20

Fig. S1.1. Bioassay for anti-antibiotic activity. (**a**) EICs of crude extracts from ΔPELP4_pCEP*pipA* (top four lines), ΔPELP4 (blue) and WT (green). EICs for the WT are pyoverdines *m/z* 665.8 $[M+2H]^{2+}$ and 657.8 $[M+2H]^{2+}$, pseudomonine *m/z* 331.1 $[M+H]^{+}$, labradorin *m/z* 241.1 $[M+H]^{+}$, and entolysines *m/z* 861.1 $[M+2H]^{2+}$, and 870.1 $[M+2H]^{2+}$. (**b** & **c**) Agar diffusion assay of different extract/blasticidin S combinations with *B. cereus* as test organism for anti-antibiotic activity testing. Concentrations of applied extracts in mg/mL are listed in the Table. 10 µL of extract and/or blasicidin S was applied on the discs and dried before placing it on the inoculated agar surface. Inhibition zones indicate the antibiotic activity of blasticidin S.

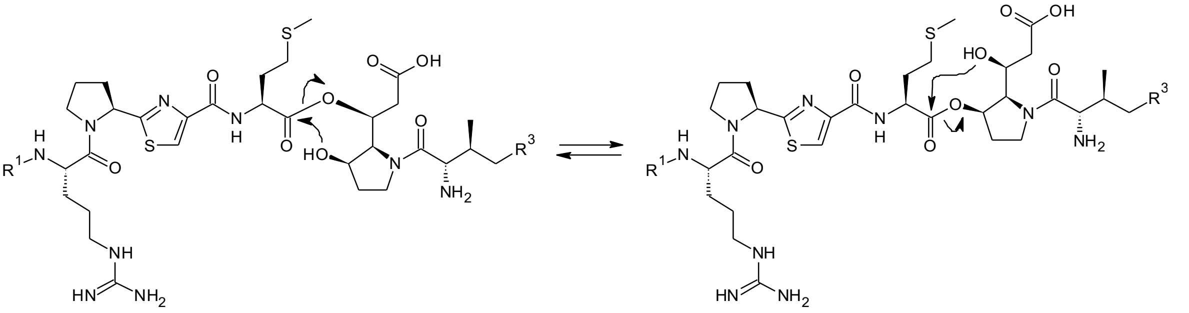

Fig. S1.2. Postulated transesterification of hydroxylated derivatives **4b** and **4d**, leading to two isobaric variants with an almost identical fragmentation pattern (see Fig. S2.7 & S2.9).

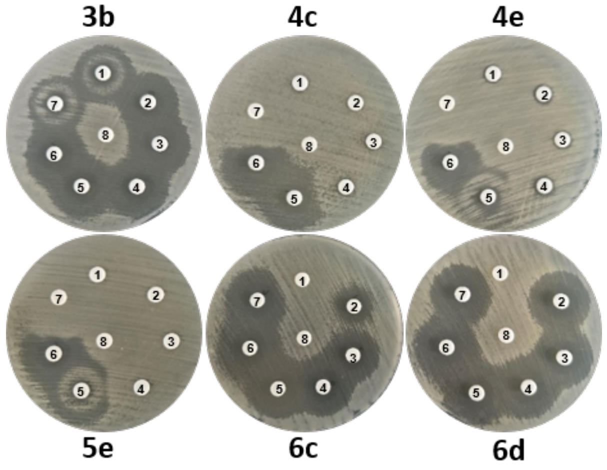

disc	BCS	compound
1	1	1
2	1	0.5
3	1	0.1
4	1	0.01
5	1	0.001
6	1	0
7	0	1
8	0	0

Fig. S1.3. Bioassay for anti-antibiotic activity. Agar diffusion assay of different compound/blasticidin S (BCS) combinations with *B. cereus* as test organism. Concentrations of BCS and compounds **3b**, **4c**, **4e**, **5e**, **6c** and **6d** in mg/mL are listed in the Table. 10 µL of extract and/or blasicidin S was applied on the discs and dried before placing it on the inoculated agar surface. Inhibition zones indicate the antibiotic activity of blasticidin S.

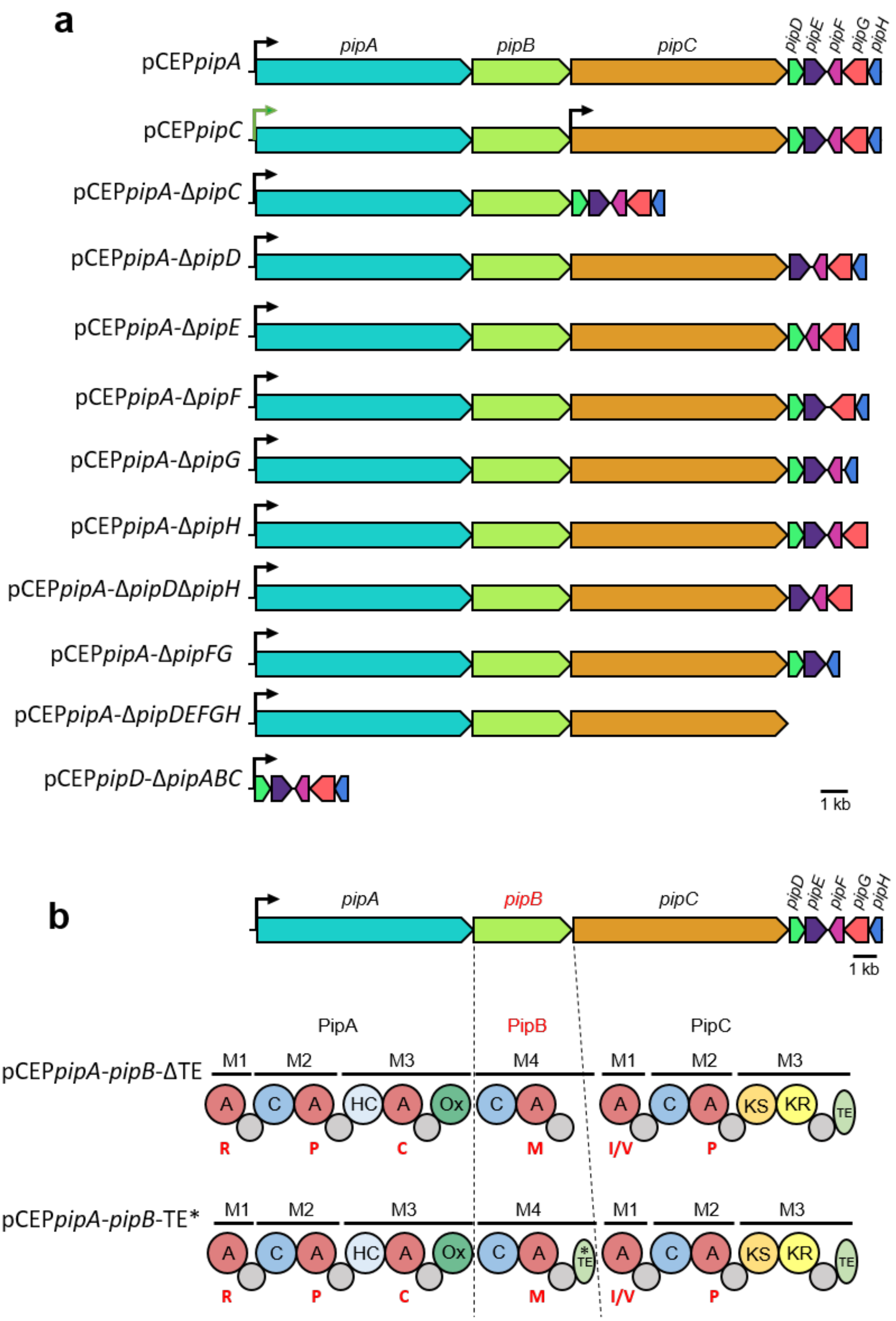

Fig. S1.4. (**a**) BGC of *pipA*-*pipH* and its genetic modifications via promoter exchanges and deletions of selected genes. Black arrow indicates the inserted promoter P_{BAD}, green arrow indicated the natural promoter. (**b**) BGC of pseudotetraivprolides focused on the modification of the TE domain in *pipB*. Top: Deletion of the TE domain, bottom: point mutation Ser2856Ala within the TE domain indicated by an asterisk. A adenylation domain, C condensation domain, HC heterocyclisation domain, Ox oxidation domain, KS keto synthase domain, KR keto reductase domain, TE thioesterase domain, small grey cycle thiolation domain.

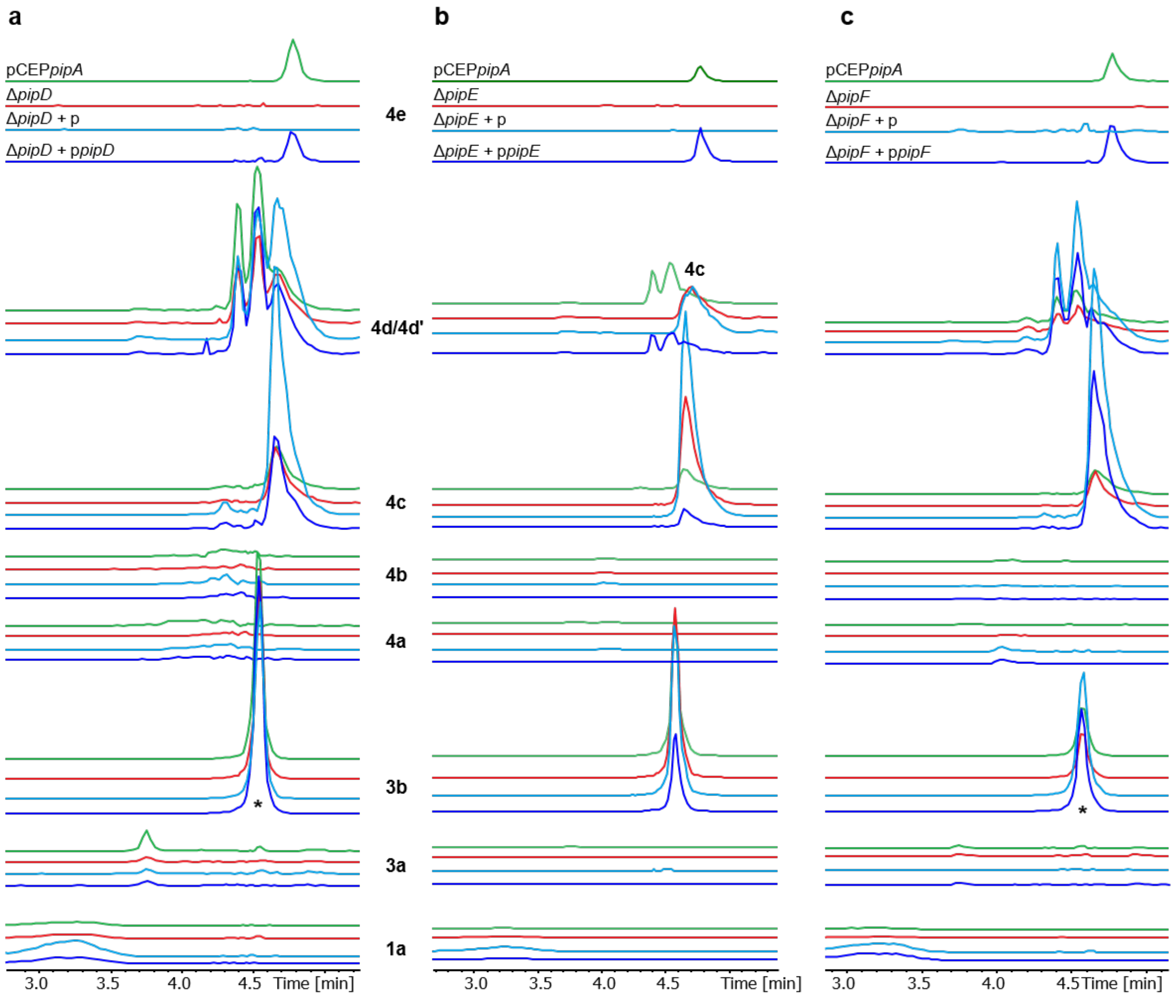

Fig. S1.5. Plasmid based complementation of deletion mutants. Depicted are the EICs of selected pip derivatives found in culture extracts of respective mutants. (**a**) Complementation of pCEP*pipA*-Δ*pipD* (red) with plasmid encoded p*pipD* (blue). (**b**) Complementation of of deletion mutant pCEP*pipA*-Δ*pipE* (red) with plasmid encoded p*pipE* (blue). (**c**) Complementation of deletion mutant pCEP*pipA*-Δ*pipF* (red) with plasmid encoded p*pipF* Shown are the EICs of selected pip derivatives found in culture extracts of respective mutants. Deletion mutants transformed with the empty vector pSEVA621-Gm as empty vector control (light blue) and compared with WT pCEP*pipA* (green). Asterisks below the peaks indicate a 10-fold reduced signal.

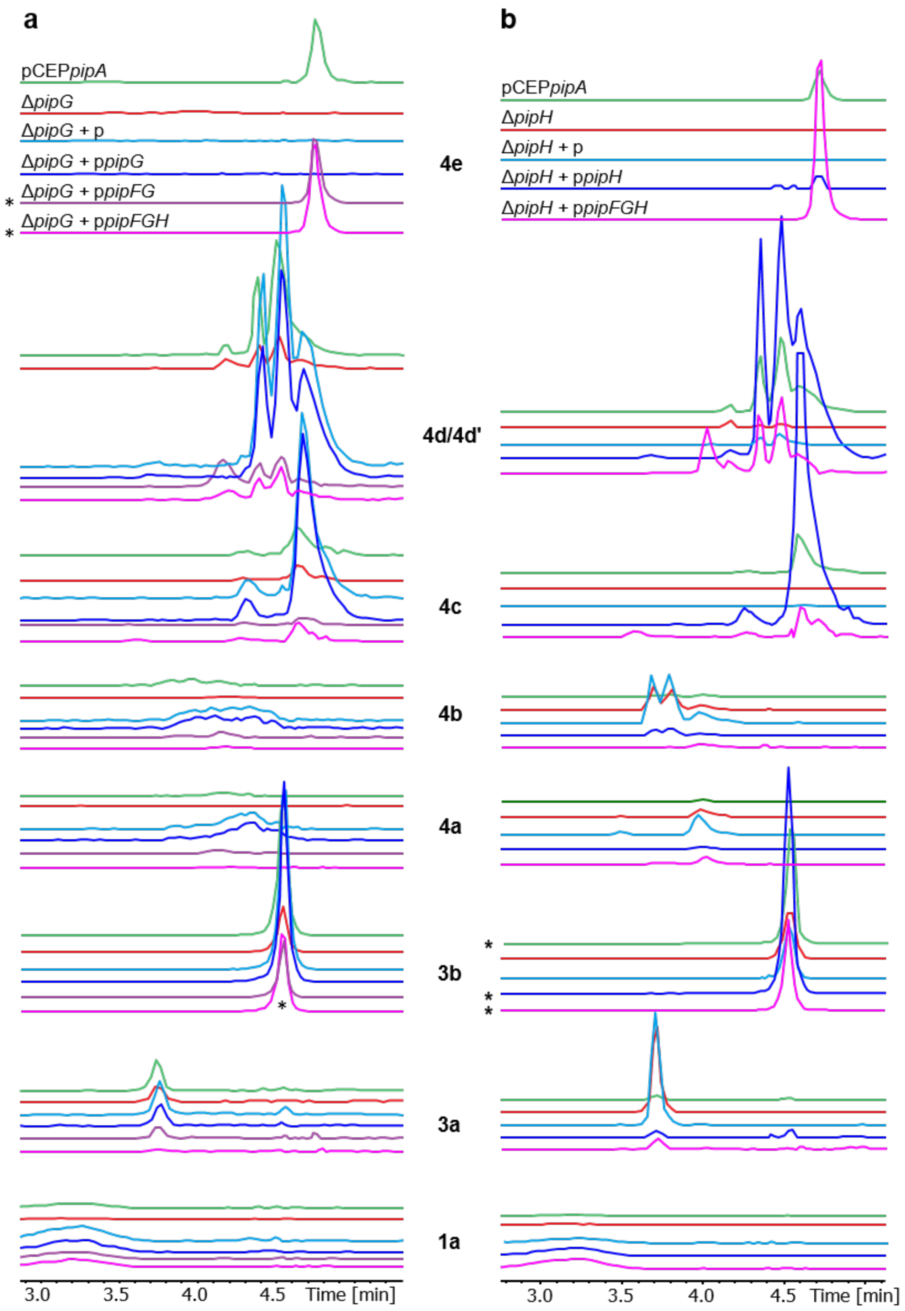

Fig. S1.6. (**a**) Plasmid based complementation of deletion mutant pCEP*pipA*-Δ*pipG* (red) with plasmid encoded p*pipG* (blue), p*pipFG* (violet) and p*pipFGH* (magenta), compared to pCEP*pipA* (green) and control strain Δ*pipG* + p (light blue). Depicted are EICs of selected pip derivatives, detected in culture extracts of respective mutants. (**b**) Plasmid based complementation of deletion mutant pCEP*pipA*-Δ*pipH* (red) with plasmid encoded p*pipH* (blue) and p*pipFGH* (magenta), compared to pCEP*pipA* (green) and control strain Δ*pipH* + p (light blue). Depicted are EICs of selected pip derivatives, detected in culture extracts of respective mutants. Asterisk indicate a 10-fold decreased signal.

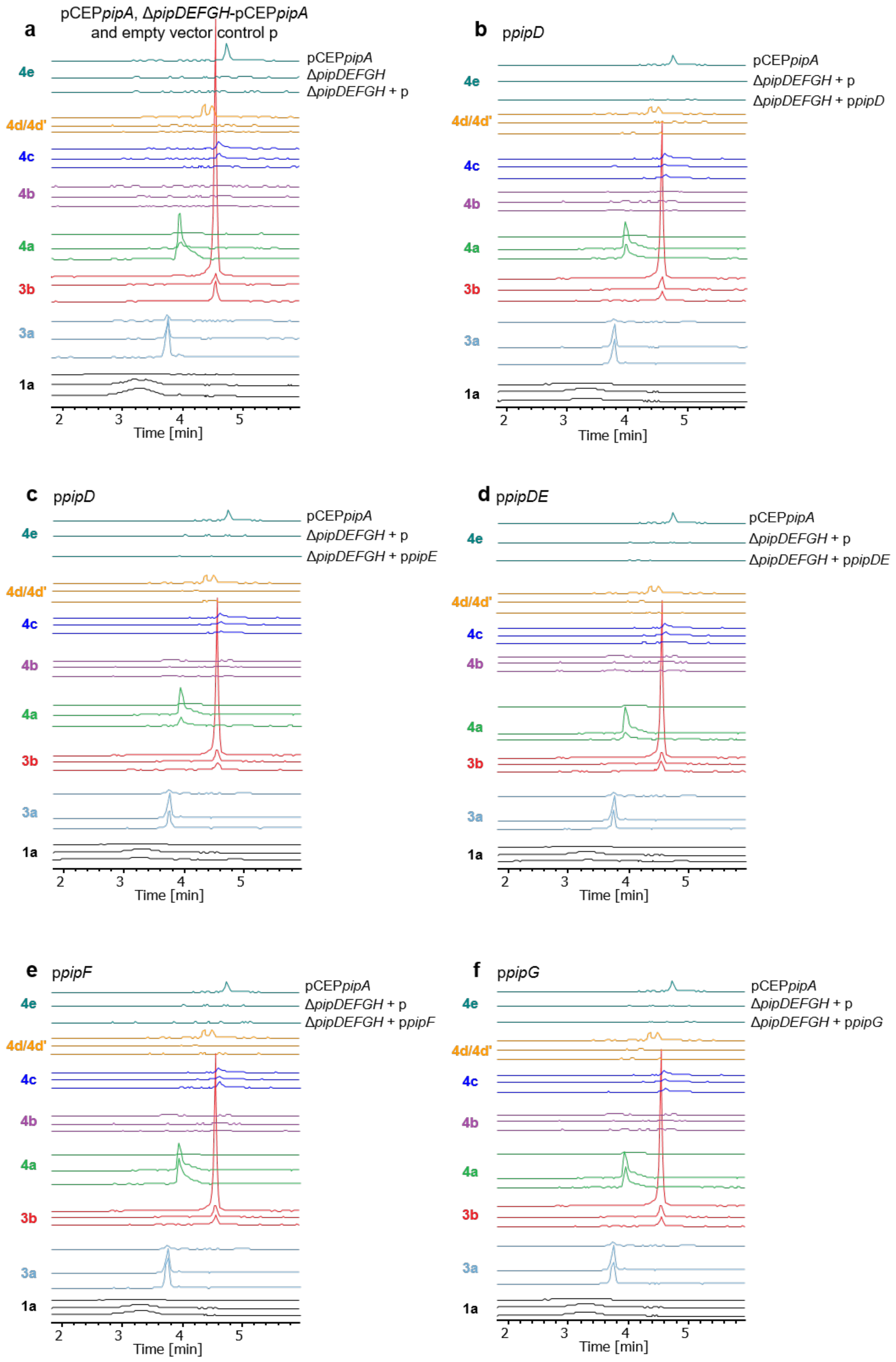
a
pCEPpipA, ΔpipDEFGH-pCEPpipA
and empty vector control p
pCEPpipA
ΔpipDEFGH
ΔpipDEFGH + p
4e
4d/4d'
4c
4b
4a
3b
3a
1a
2
3
4
5
Time [min]
b
ppipD
pCEPpipA
ΔpipDEFGH + p
ΔpipDEFGH + ppipD
c
ppipD
pCEPpipA
ΔpipDEFGH + p
ΔpipDEFGH + ppipE
d
ppipDE
pCEPpipA
ΔpipDEFGH + p
ΔpipDEFGH + ppipDE
e
ppipF
pCEPpipA
ΔpipDEFGH + p
ΔpipDEFGH + ppipF
f
ppipG
pCEPpipA
ΔpipDEFGH + p
ΔpipDEFGH + ppipG

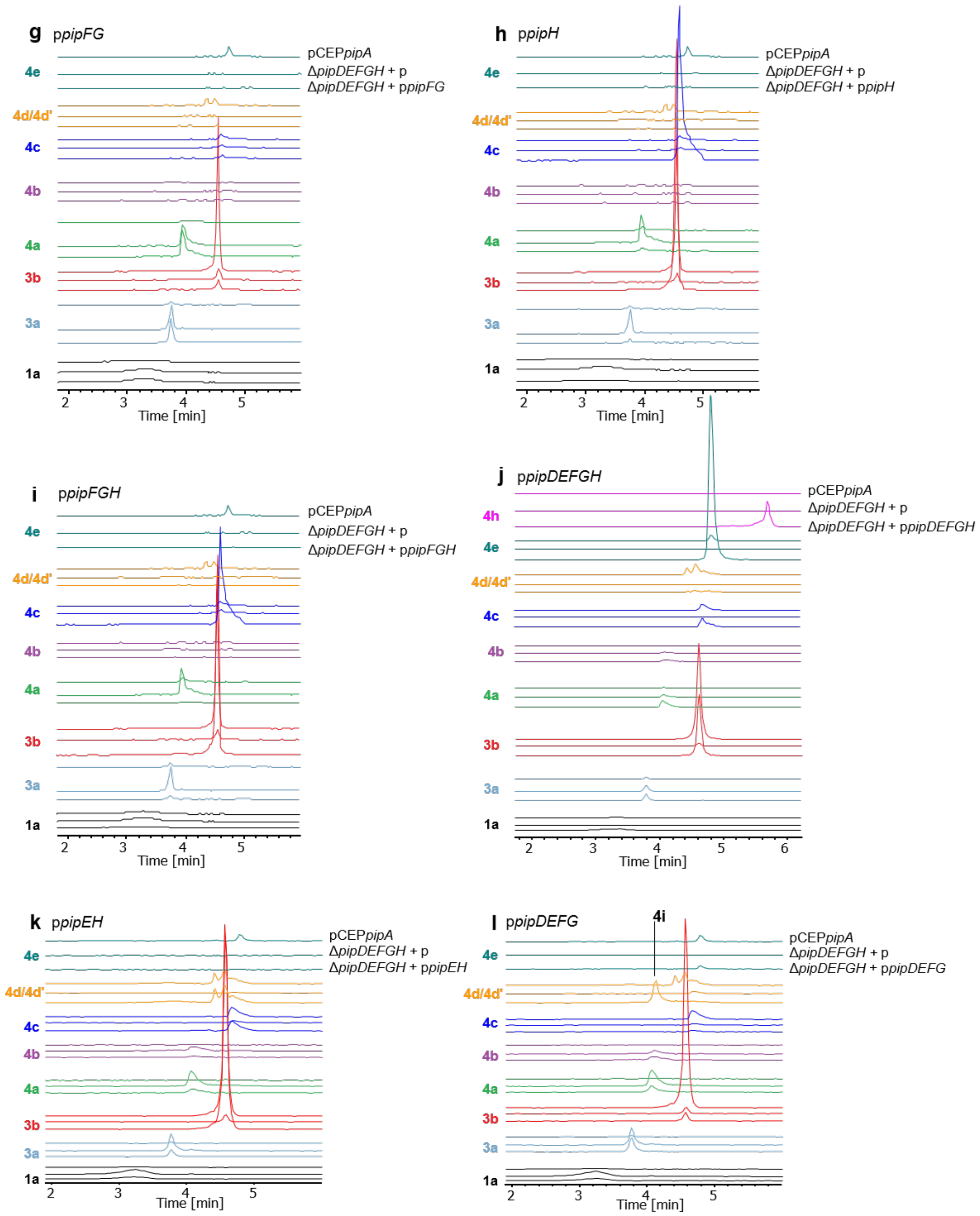

g ppipFG
pCEPpipA
ΔpipDEFGH + p
ΔpipDEFGH + ppipFG
4e
4d/4d'
4c
4b
4a
3b
3a
1a
2
3
4
5
Time [min]
h ppipH
pCEPpipA
ΔpipDEFGH + p
ΔpipDEFGH + ppipH
i ppipFGH
pCEPpipA
ΔpipDEFGH + p
ΔpipDEFGH + ppipFGH
j ppipDEFGH
pCEPpipA
ΔpipDEFGH + p
ΔpipDEFGH + ppipDEFGH
4h
6
k ppipEH
pCEPpipA
ΔpipDEFGH + p
ΔpipDEFGH + ppipEH
l ppipDEFG
4i
pCEPpipA
ΔpipDEFGH + p
ΔpipDEFGH + ppipDEFG

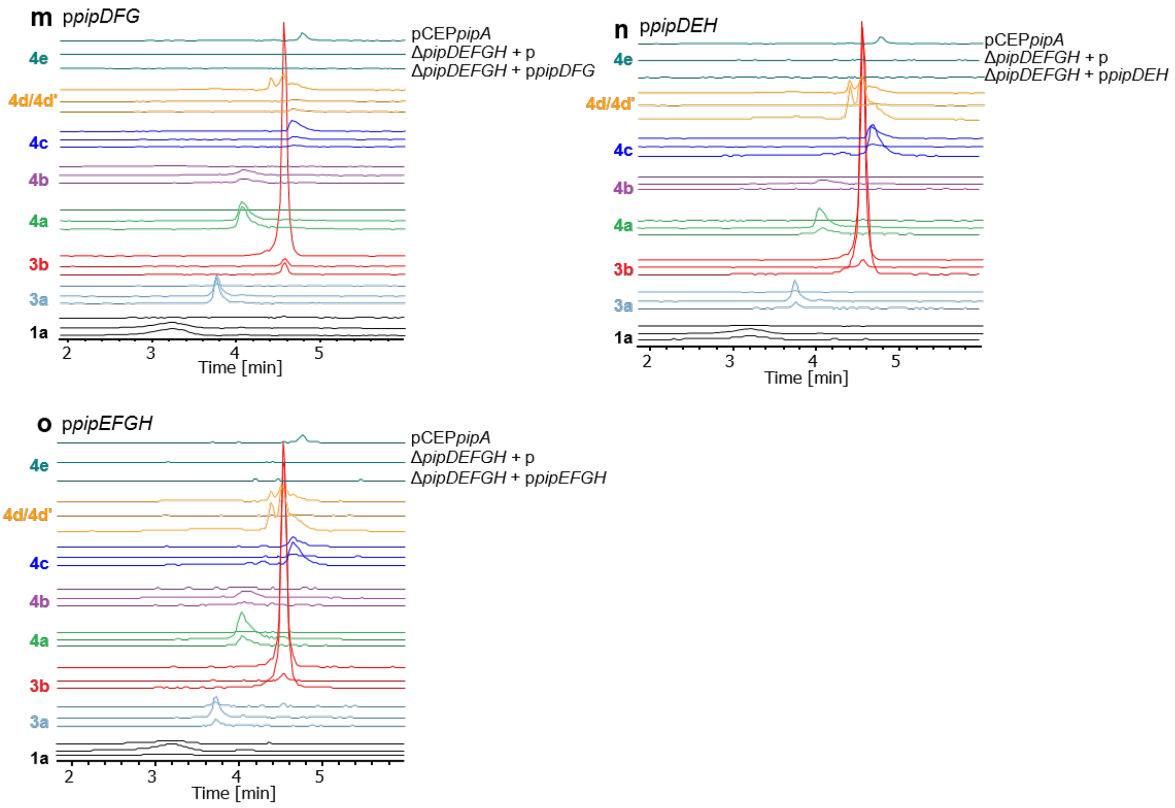

Fig. S1.7. Plasmid based complementation of deletion mutant Δ*pipDEFGH*-pCEP*pipA*. Shown are the EICs of selected compounds from culture extracts after HPLC/MS analysis of respective mutants. (**a**) pCEP*pipA*, Δ*pipDEFGH*-pCEP*pipA* and empty vector control p (**b**) p*pipD*, (**c**) p*pipE*, (**d**) p*pipDE*, (**e**) p*pipF*, (**f**) p*pipG*, (**g**) p*pipFG*, (**h**) p*pipH*, (**i**) p*pipFGH*, (**j**) p*pipDEFGH*, (**k**) p*pipEH*, (**l**) p*pipDEFG*, (**m**) p*pipDFG*, (**n**) p*pipDEH* (**o**) p*pipEFGH*.

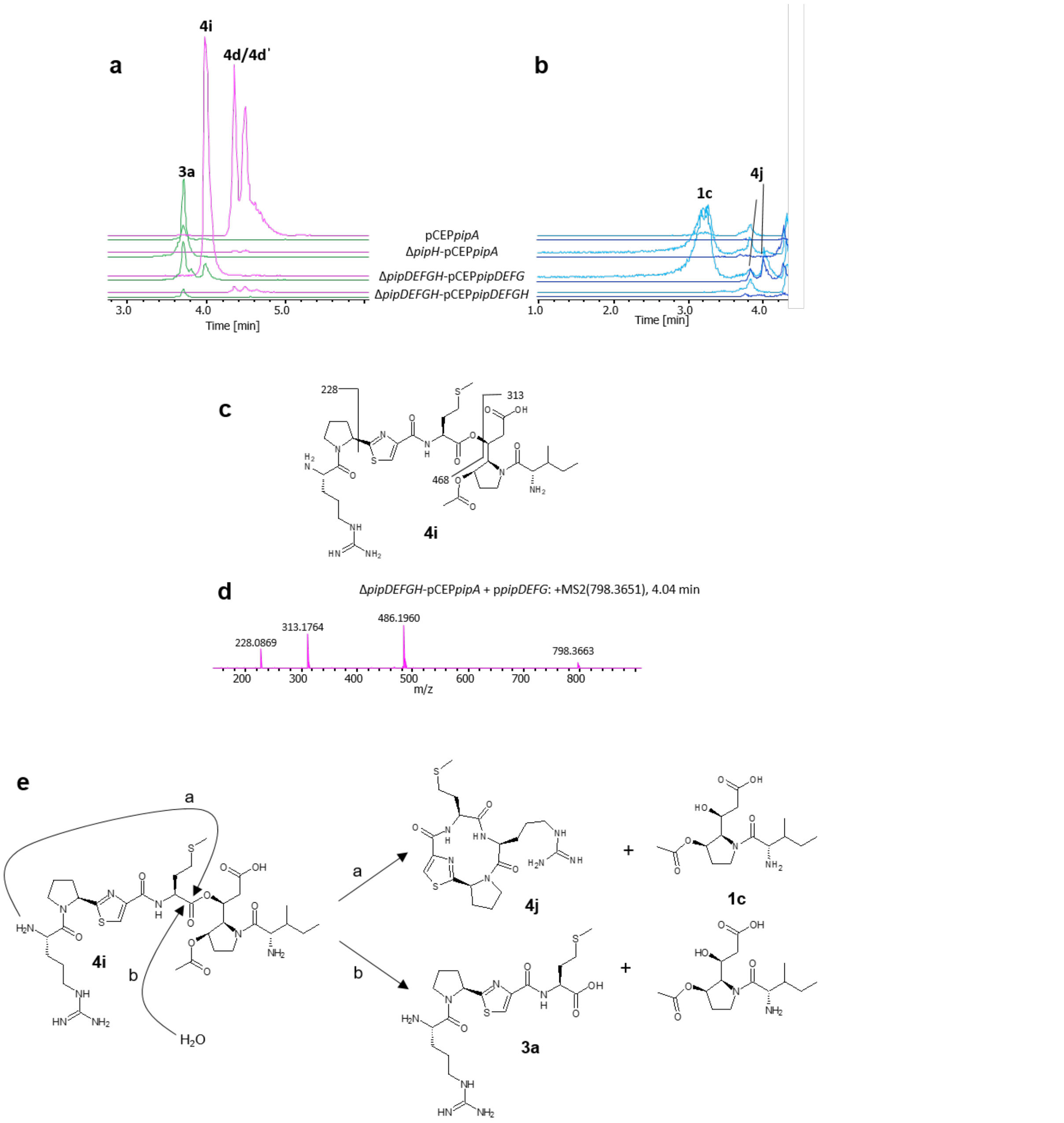

Figure S1.8. Identification and structure elucidation of **4i** and **4j** produced by Δ*pipDEFGH*-pCEP*pipA* complemented with p*pipDEFG*. (**a**) EICs of selected compounds produced by pCEP*pipA*, Δ*pipH*-pCEP*pipA*, Δ*pipDEFGH*-pCEP*pipA* + p*pipDEFGH* and Δ*pipDEFGH*-pCEP*pipA* + p*pipDEFG*. Structure (**c**) and MS/MS data (**d**) of **4i**, and (**e**) proposed hydrolysis of **4i** into **4j**, **3a** and **1c**.

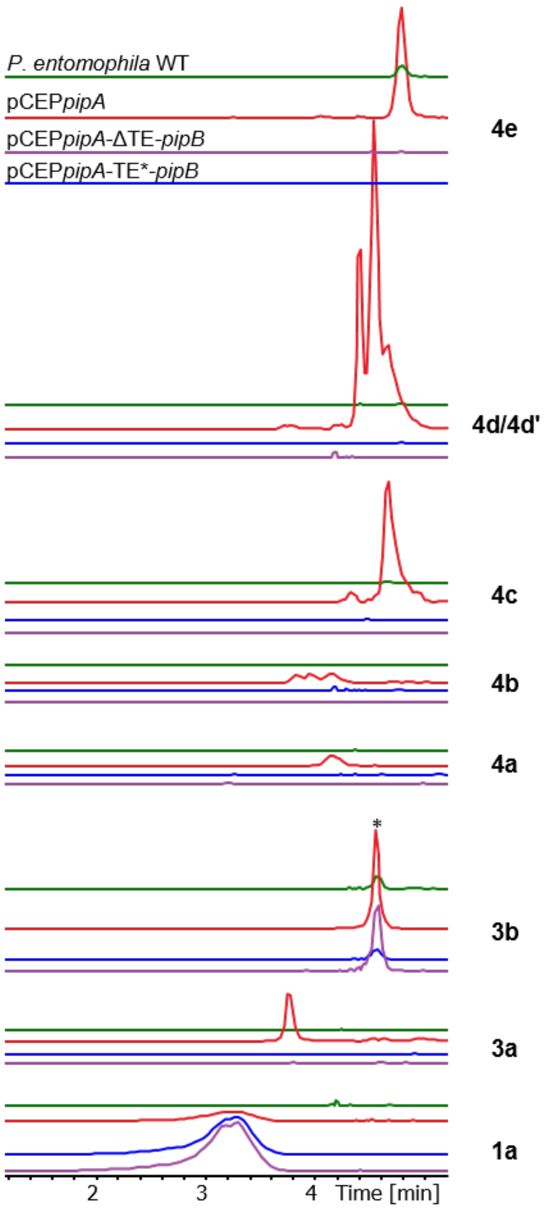

Fig. S1.9. Investigation of the TE domain of *pipB*. EICs of selected derivatives of pseudotetraivprolide detected in culture extracts of *P. entomophila* WT (green), pCEP*pipA* (red), pCEP*pipA*-ΔTE-*pipB* (blue) and pCEP*pipA*- TE*-*pipB* (violet). Asterisk indicate a 20-fold reduced signal.

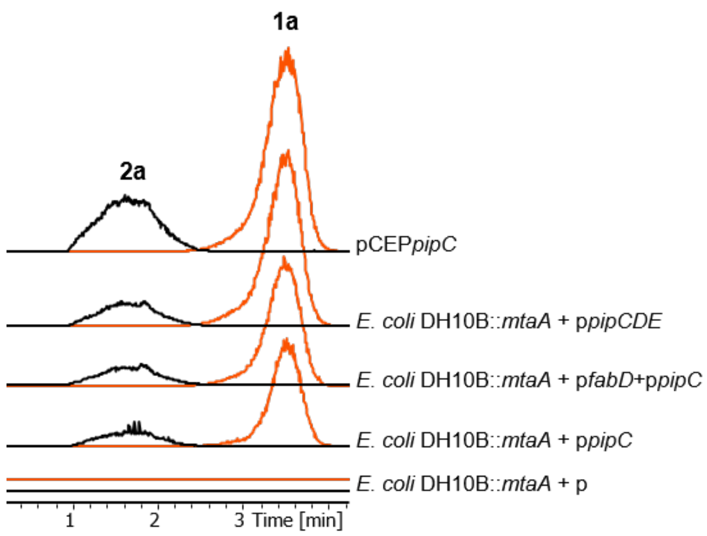

Fig. S1.10. Heterologous expression of p*pipCDE* and pPSEEN_RS07520 (p*fabD*) in *E. coli* DH10B::*mtaA*. EICs of **1a** and **2a** in HPLC/MS analysis of culture extracts from pCEP*pipC*, plasmid encoded p*pipC* and co-expression of plasmid encoded p*pipC* and p*fabD* in *E. coli* DH10B::*mtaA*. No hydroxylated or O-acetylated derivatives, **1b** and **1c,** were detected.

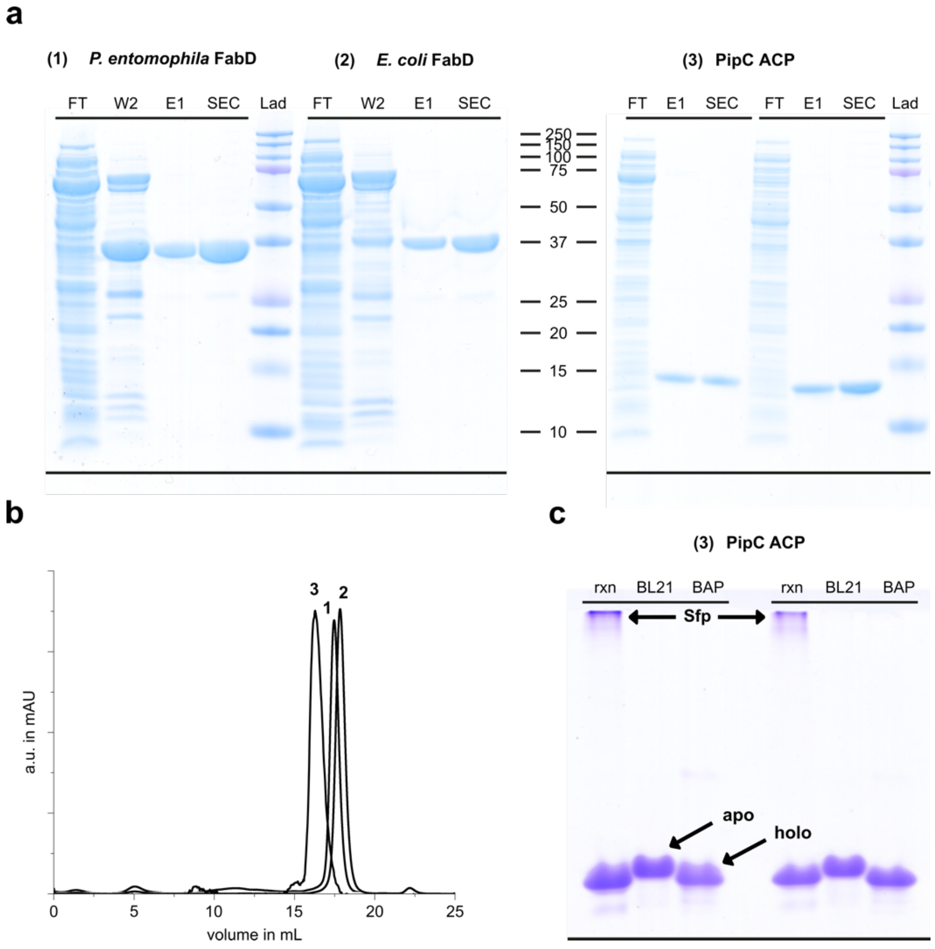

Figure S1.11. Protein Quality Control of *P. entomophila* FabD, *E. coli* FabD and PipC ACP. Highly pure protein was obtained for both AT domains as well as ACP domain (shown in biological duplicate) from heterologous expression in BAP1 cells. (**a**) SDS-PAGE to monitor purification protocol. Proteins were purified from 2-step protocol (Ni-chelating affinity chromatography followed by polishing via size exclusion chromatography). Protein sizes were observed as expected: *P. entomophila* FabD: ≈ 36.9 kDa, *E. coli* FabD: ≈ 37.1 kDa, PipC ACP: ≈ 14.0 kDa (FT = flowthrough, E1 = Elution, SEC = Size Exclusion Chromatography, Lad = Precision Plus Protein Ladder). (**b**) Size exclusion chromatograms of purified proteins. *P. entomophila* FabD (1; V_E = 17.5 mL) and *E. coli* FabD (2; V_E = 17.8 mL) were purified using a Superose™ 6 Increase 10/300 GL column, and PipC ACP (3; V_E = 16.2 mL) was purified via a Superdex™ 200 Increase 10/300 GL column. (**c**) Conformation-sensitive urea PAGE for analysis of PipC ACP domain. PipC ACP was expressed in apo- (BL21 (DE3), middle) and holo-form (BAP1, genetically-encode 4'-phosphopantetheinyl transferase from *Bacillus subtilits* (Sfp), right). For comparison, phosphopantetheinylation was conducted *in vitro* using purified Sfp, CoA-SH and apo-ACP (expressed in BL21 (DE3) cells, left). Expression of PipC ACP in BAP1 cells solely provided holo-ACP.

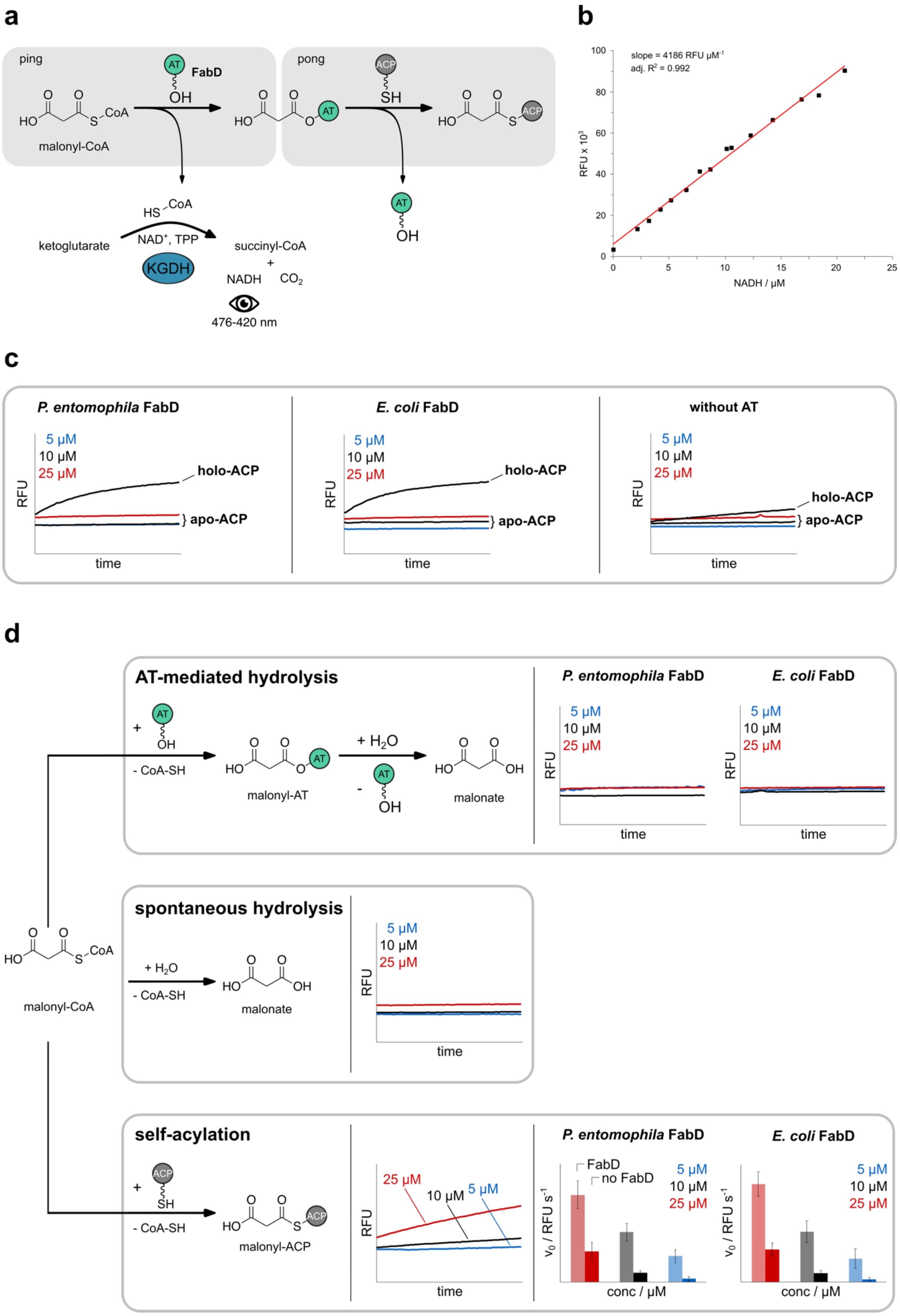

Figure S1.12. Overview of the alpha-KGDH assay. (**a**) AT-mediated transacylation reaction can be monitored via an enzyme-coupled assay. AT domains catalyze the transfer of an CoA-activated acyl-moiety (donor substrate; here: malonyl-CoA, Mal-CoA) to a holo-ACP domain

(acceptor substrate; here: PipC ACP) via a double displacement reaction (ping-pong bi-bi mechanism). Upon release of CoA-SH, the acyl-moiety is loaded onto the AT domain (ping step). Next, the acyl-moiety is transferred onto the ACP-domain to give acyl-ACP (pong step). The release of CoA-SH can be couple to the KGDH-catalyzed formation of succinyl-CoA from ketoglutarate, CoA-SH and NAD^+. The reduction of NAD^+ to NADH can be monitored fluorometrically and enables quantification of the transacylation rate. (**b**) NADH calibration curve. To quantify AT activity, a NADH calibration was used to correlate relative fluorescence units (RFU) to NADH concentration (in µM). Measurement was performed in technical triplicates (**c**) Influence of apo- and holo-ACP on AT-mediated transacylation reaction. Equimolar amounts of malonyl-CoA and PipC apo-ACP were screened in presence and absence of trans-AT domain (substrate concentrations: 5 µM: blue; 10 µM: black; 25 µM: red). RFU is blotted over time. An increase of RFU would indicate CoA-SH release. For comparison, holo-ACP was measured at a substrate concentration of 10 µM (black). PipC apo-ACP is not active in transacylation. PipC holo-ACP is required for efficient transfer of a malonyl-unit. Measurement performed in technical triplicates of biological duplicates. (**d**) Several side reactions can occur during the AT-mediated transacylation that need to be taken into account when determining kinetic parameters. Eventually, formation of large quantities of CoA-SH can falsify kinetic constants. (Top) AT-mediated hydrolysis of malonyl-CoA in the absence of PipC ACP. Steady RFU values indicate that AT-mediated hydrolysis by *P. entomophila* FabD or *E. coli* FabD does not occur in the assay setup. (Center) Spontaneous hydrolysis of malonyl-CoA in the absence of PipC ACP and trans-AT domain. Similarly, constant RFUs suggest no formation of free CoA-SH when trans-AT domain and donor substrate are absent. (Bottom) Self-acylation of malonyl-CoA onto PipC ACP in the absence of trans-AT domain. At higher substrate concentrations (≥25 µM; red curve), self-acylation is a significant background reaction. Yet, initial velocities of transacylation are substantially higher when a trans-AT domain is present demonstrating its involvement in PipC ACP loading (column graphs). Consequently, initial velocities of active measurements (in the presence of trans-AT) have been corrected by initial velocities of self-acylation reactions (absence of trans-AT). Measurements were performed in technical triplicates. Blots are shown as average of biological duplicates.

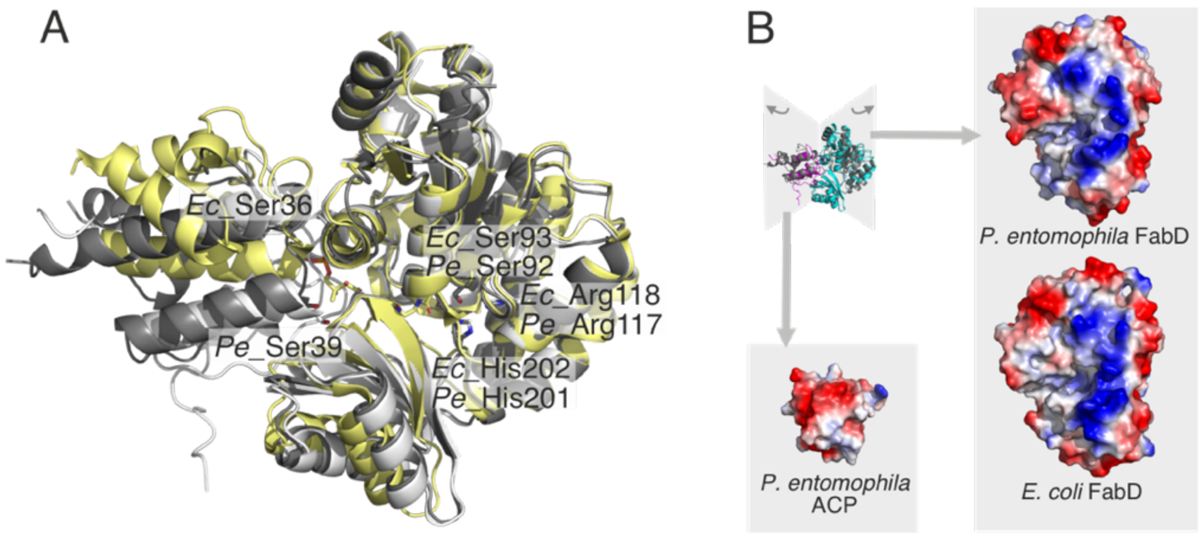

Figure S1.13. Structure of ACP-AT interaction. (**A**) ACP-AT complexes of *P. entomophila* ACP (PipC module 3) and *P. entomophila* AT (dark grey), *P. entomophila* ACP (PipC module 3) and *E. coli* FabD (white), both complexes modeled with AlphaFold3, and experimental structural data on the *E. coli* ACP-FabD complex (yellow) (PDB-ID 6u0j). Selected residues are highlighted in stick representation, as well as the covalent crosslinker in the FabD binding pocket. The predicted structures superimpose well in the ACP positioning, while the original data on the *E. coli* complex show a tilted position. The serine, carrying the phosphopantetheine after post-translational modification is plausible position, meaning that a phosphopantetheine could reach the AT active site. (**B**) Vacuum electrostatics of the ACP:AT interface. The binding interface is shown for both complexes (*P. entomophila* ACP-*E. coli* FabD complex colored superimposed with *P. entomophila* complex in grey). The ACP interface is negatively charged and the opposing AT interface positively charged, indicated molecular recognition that drives interaction. The figure is prepared with pymol. Structures have been superimposed with the pymol superposition tool and the charge distribution on the protein surfaces estimated with the pymol vacuum electrostatics tool (www.pymol.org).

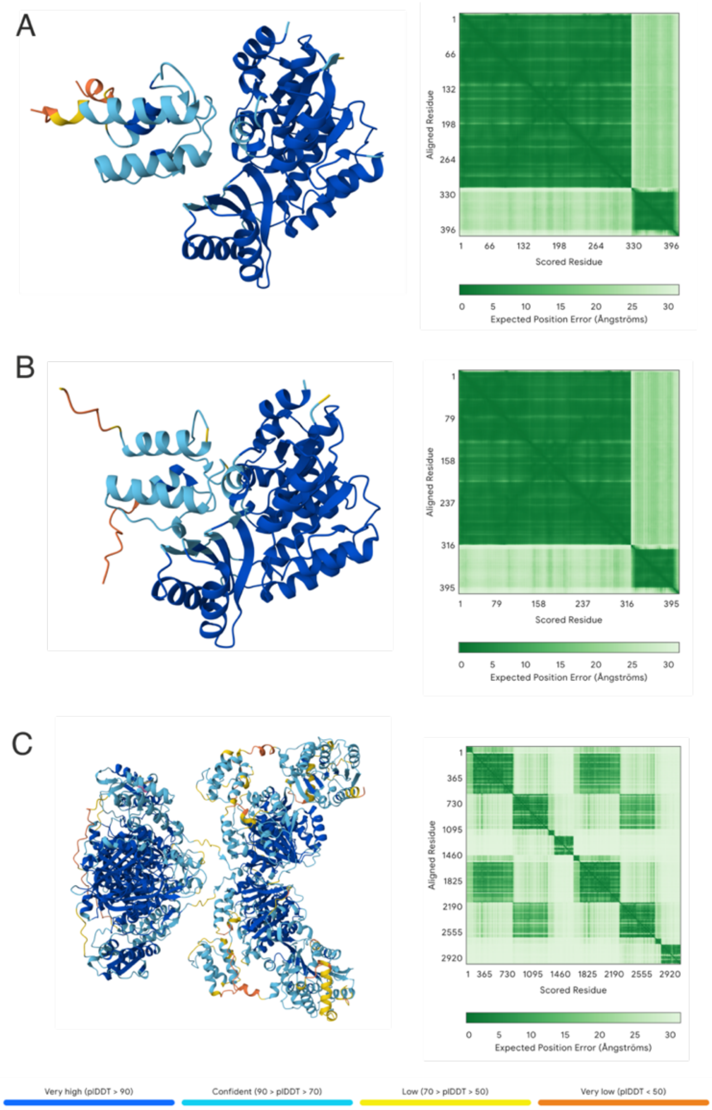

Figure S1.14. Assessment of model confidence and structural accuracy. Quality assessment for predicted structures of *P. entomophila* ACP (PipC module 3): *P. entomophila* AT (A), *P. entomophila* ACP:*E. coli* FabD (B), and PipC module 3 with N-terminal ACP domain (C). Orientation of structures as in Fig. 5. (Left panels) per-residue confidence scores (pLDDT) predicted by AlphaFold3, plotted across the sequence. Higher pLDDT values (>90) indicate regions of high structural reliability, while lower scores (<70) suggest flexible or uncertain regions. (Right panels) Predicted Alignment Error (PAE) plot showing the estimated positional error between residue pairs. Lower PAE values between residues from different domains indicate high confidence in their relative positioning, while higher values reflect greater uncertainty.

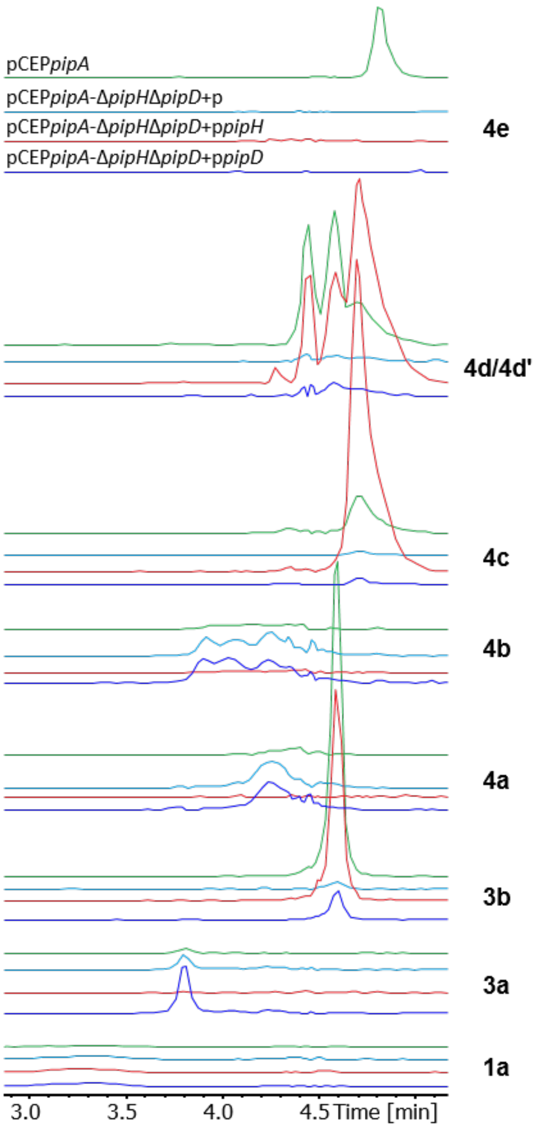

Fig. S1.15. Investigation on the activities of PipH and PipD regarding N-terminal acetylation. EICs of selected derivatives detected by HPLC/MS analysis in culture extracts of pCEP*pipA* (green), pCEP*pipA*-Δ*pipD*Δ*pipH*+p (light blue) and complemented deletion mutants pCEP*pipA*-Δ*pipD*Δ*pipH*+p*pipH* (red) and pCEP*pipA*-Δ*pipD*Δ*pipH*+p*pipD* (blue).

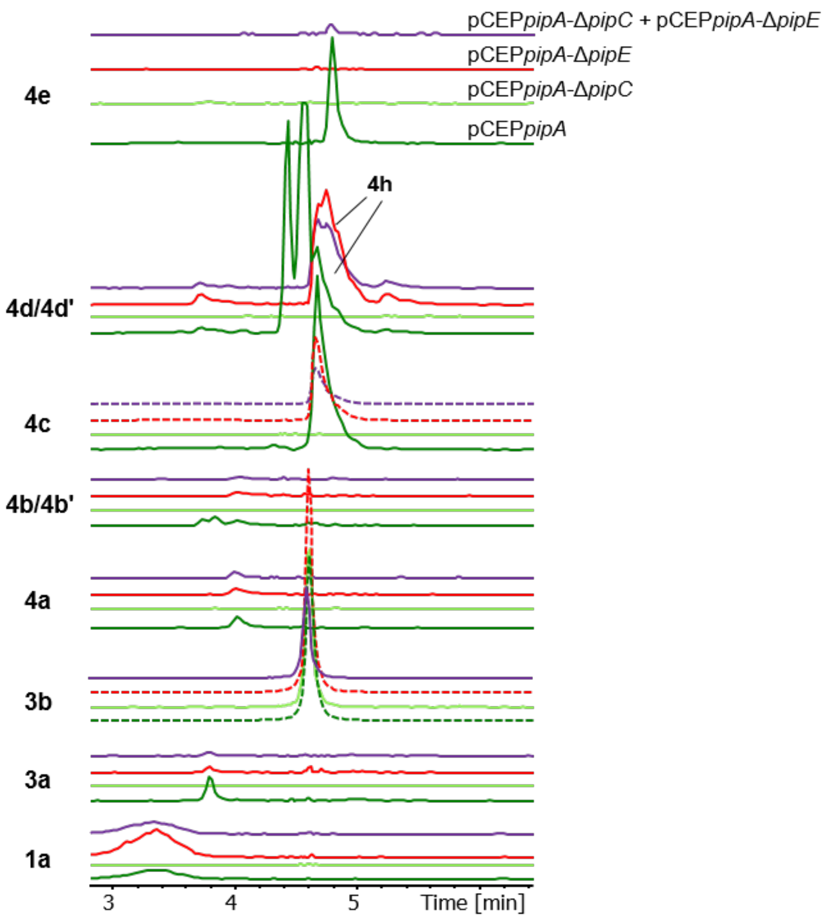

Fig. S1.16. Derivatives of pseudotetraivprolide detected in extracts of co-cultivated pCEP*pipA*-Δ*pipE* + pCEPpipA-Δ*pipC* (violet) in comparison to derivatives produced by single cultivated mutants pCEP*pipA* (green), pCEP*pipA*-Δ*pipE* (red), pCEP*pipA*-Δ*pipC* (neon green). Dashed lines indicate a 10-fold reduced signal.

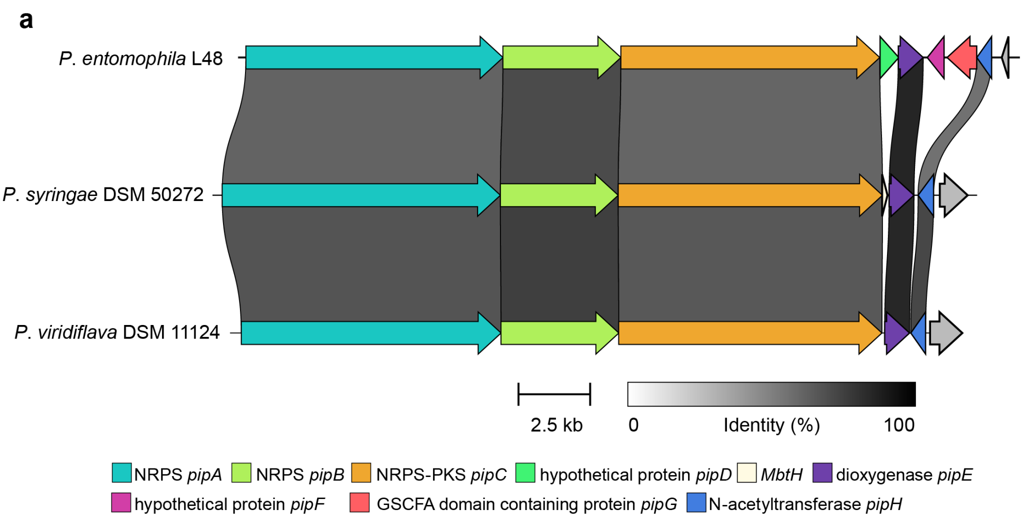

Fig. S1.17. Organization of pip BGC in *P. entomophila*, *P. syringae* and *P. viridiflava* analyzed via CAGECAT analysis.

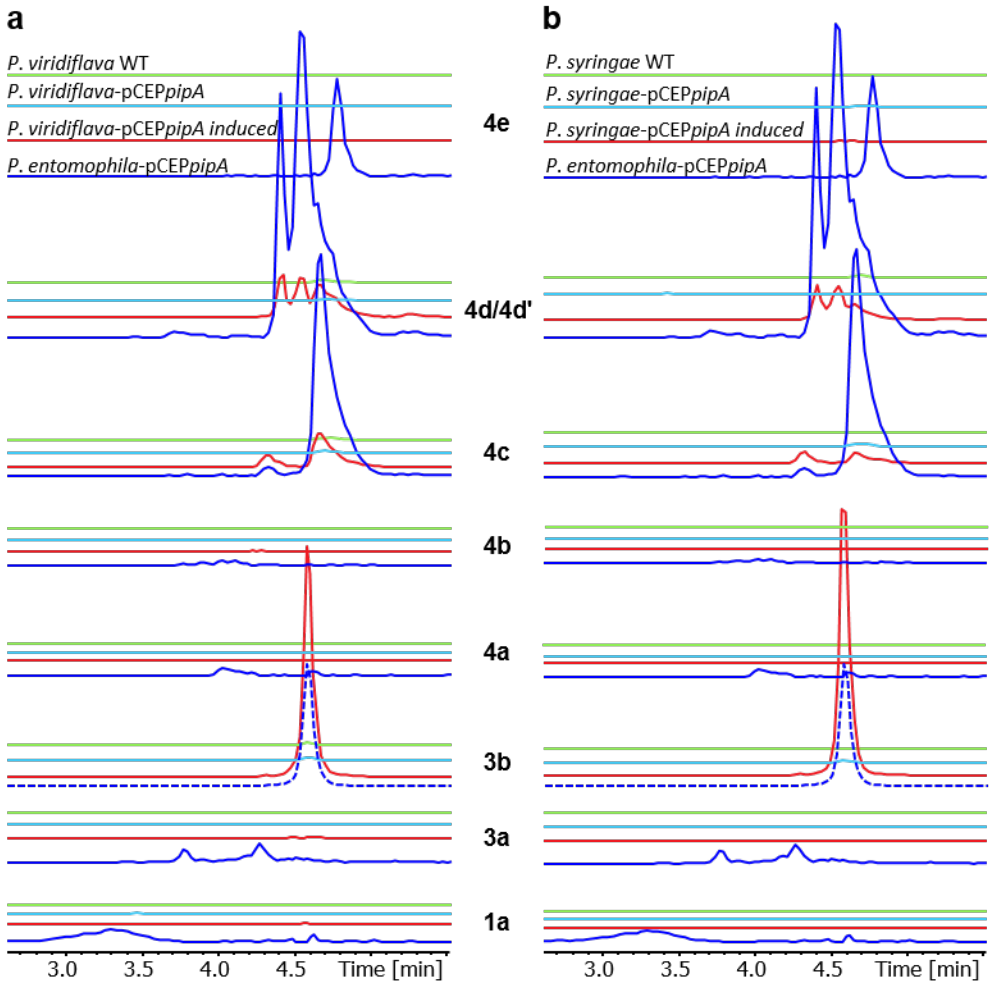

Fig. S1.18. (**a**) EICs of selected pip derivatives detected via HPLC/MS analysis in culture extracts of *P. viridiflava* WT (green), *P. viridiflava*-pCEP*pipA* non induced (turquoise), induced (red) and *P. entomophila*-pCEP*pipA* induced (blue). (**b**) *P. syringae* WT (green), *P. syringae*-pCEP*pipA* non-induced (turquoise) and *P. syringae*-pCEP*pipA* induced (red). Dashed lines indicate a 10-fold decreased signal.

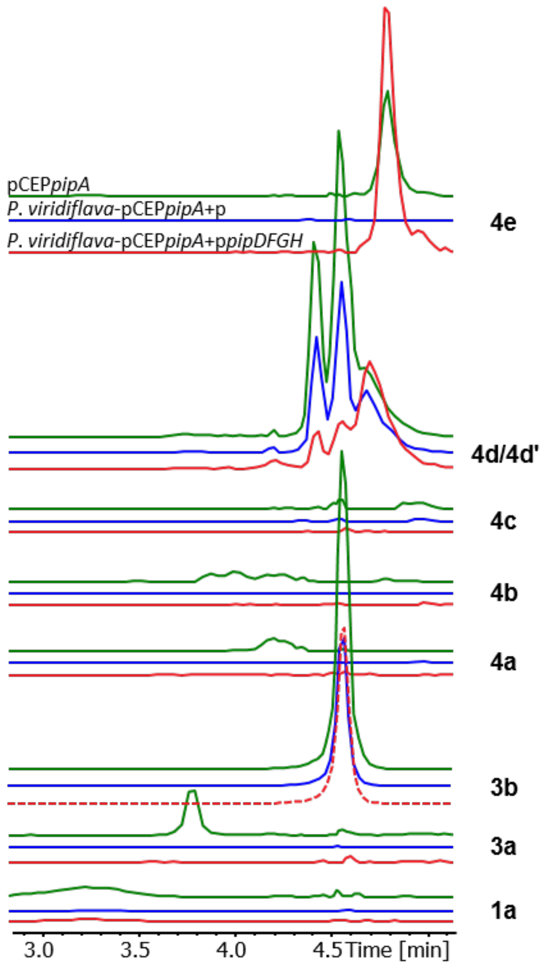

Fig. S1.19. Complementation of *P. viridiflava*-pCEP*pipA* with p*pipDFGH*. EICs of selected derivatives detected via HPLC/MS analysis in culture extracts of pCEP*pipA* (green), *P. viridiflava*-pCEP*pipA*+p induced (blue) and *P. viridiflava*-pCEP*pipA*+p*pipDFGH* induced (red).

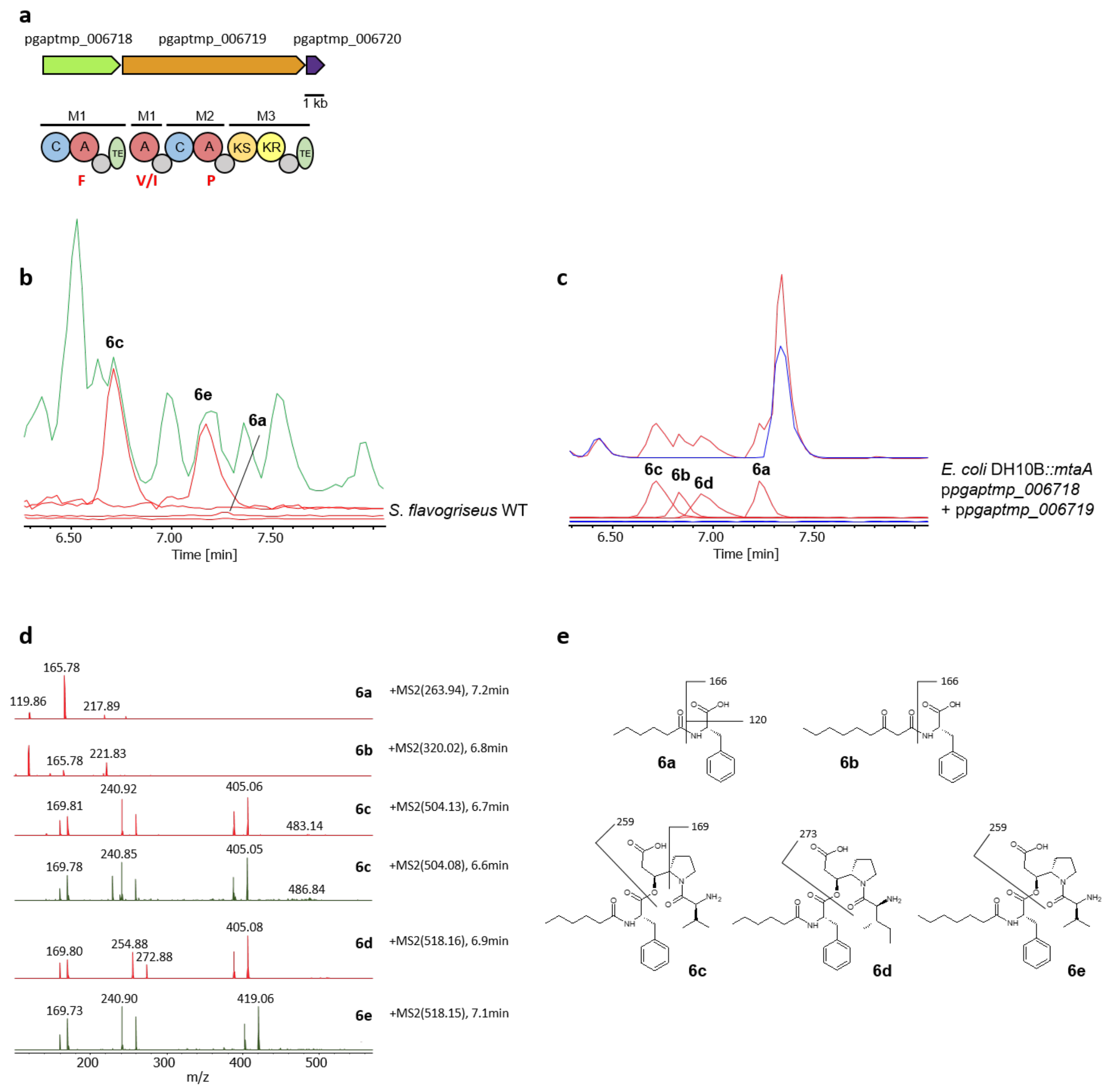

Figure S1.20. (**a**) Detoxin BGC (encoding Sf-PipBCE) of *S. flavogriseus* and the modular organization of the encoded NRPS-NRPS/PKS hybrid. (**b**) BPC (green) and EICs (red) of detoxin derivatives produced by *S. flavogriseus* WT in TSBY medium. (**c**) Detoxin derivatives from *S. flavogriseus* produced heterologously in *E. coli* DH10B::*mtaA_ppgaptmp_006718* + *ppgaptmp_006718* non-induced (blue) and induced (red). (**d**) MS^2-fragmentation pattern of **6a**, **6b**, **6c**, **6d** and **6e** detected in culture extracts of *E. coli* DH10B::*mtaA_ppgaptmp_006718* + *ppgaptmp_006718* (red) and **6c** and **6e** detected in culture extracts of *S. flavogriseus* (green). (**e**) Proposed chemical structure of **6a**, **6b**, **6c**, **6d** and **6e**.

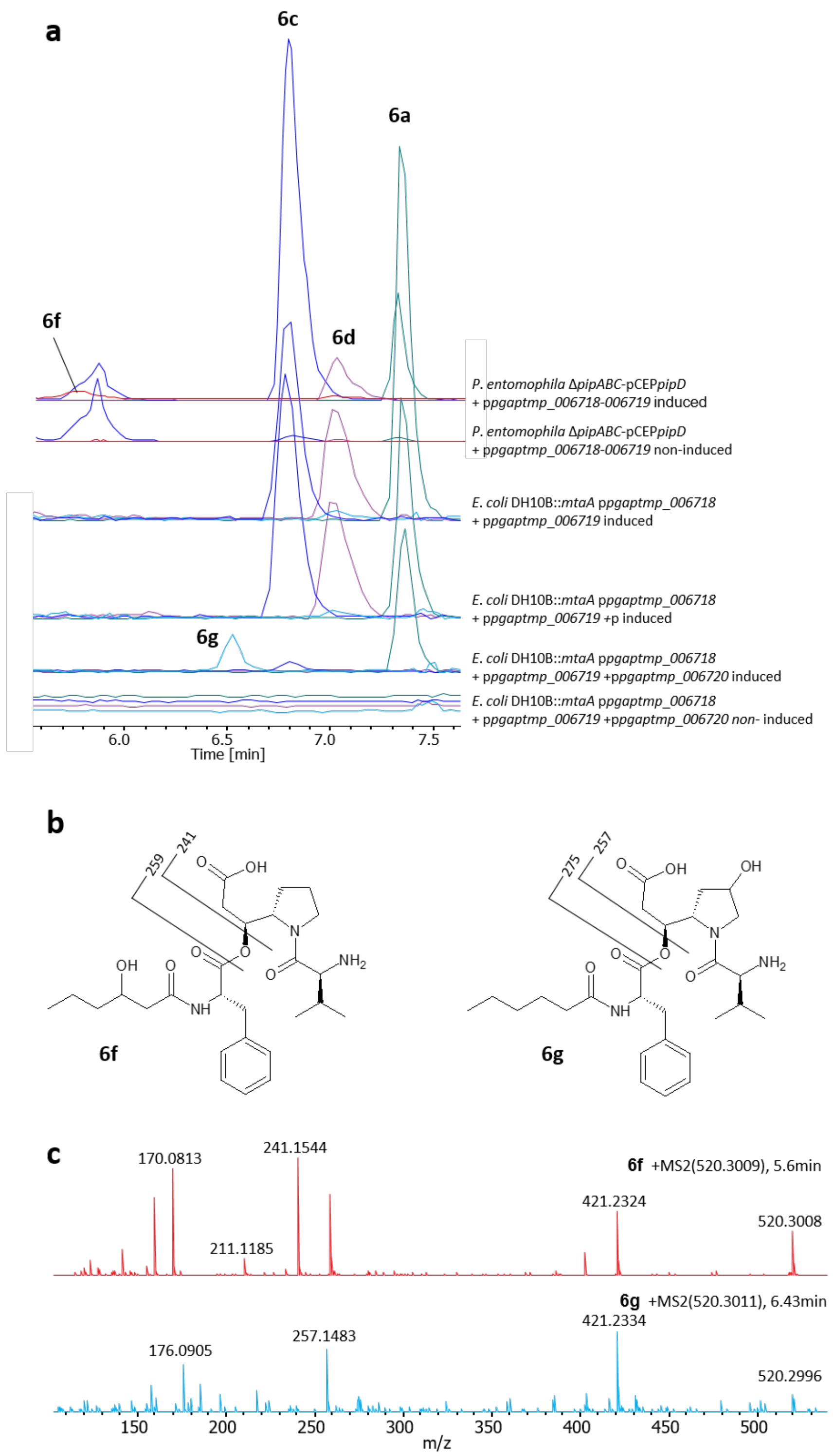

Figure S1.21. Heterologous expression of the detoxin BGC (*ppgaptmp_006718-006720*) in *P. entomophila*Δ*pipABC*-pCEP*pipD* and *E. coli* DH10B::*mtaA*. (**a**) From top to bottom: EICs of selected compounds detected in culture extracts of induced and non-induced *P. entomophila* Δ*pipABC*-pCEP*pipD* + p*pgaptmp_006718-006719*, in induced *E. coli* DH10B::*mtaA* p*pgaptmp_006718* + p*pgaptmp_006719*, induced *E. coli* DH10B::*mtaA* p*pgaptmp_006718* + p*pgaptmp_006719* +p (empty plasmid control); and induced and non-induced *E. coli* DH10B::*mtaA* p*pgaptmp_006718* + p*pgaptmp_006719* +p*pgaptmp_006720*). (**b**) Chemical structures of **6f** and **6g**. (**c**) MS^2 fragmentation pattern of **6f** (red) and **6g** (turquoise).

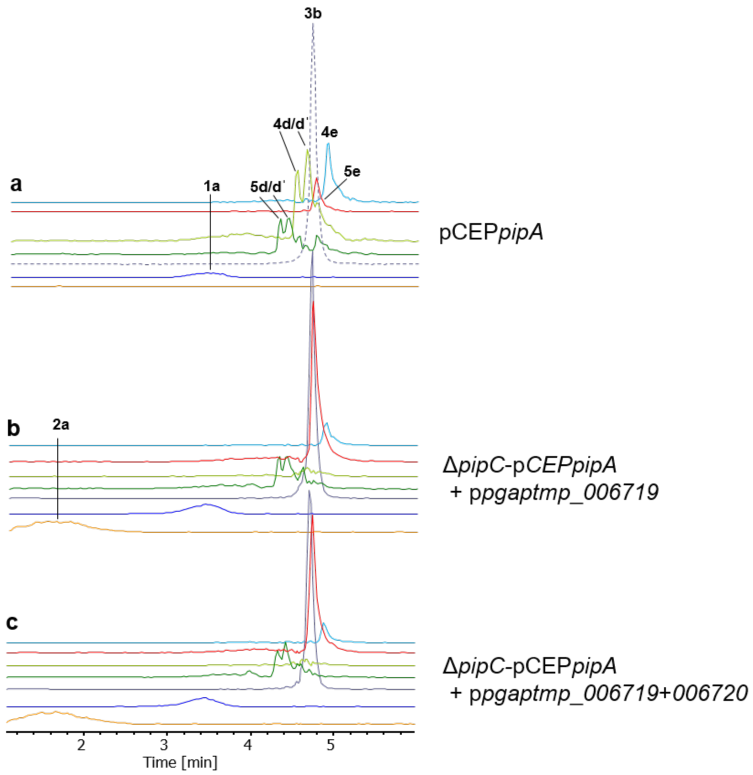

Fig. S1.22. Expression of plasmid encoded genes of the detoxin-producing BGC from *S. flavogriseus* in pCEP*pipA*-Δ*pipC*. (**a**) Full set of pseudotetraivprolide derivatives produced by induced pCEP*pipA*, (**b**) derivatives produced by Δ*pipC*-pCEP*pipA_*p*pgaptmp_006719* (**c**) derivatives produced by pCEP*pipA*-Δ*pipC* _p*pgaptmp_006719-006720*. Dashed line indicates a twofold reduced signal.

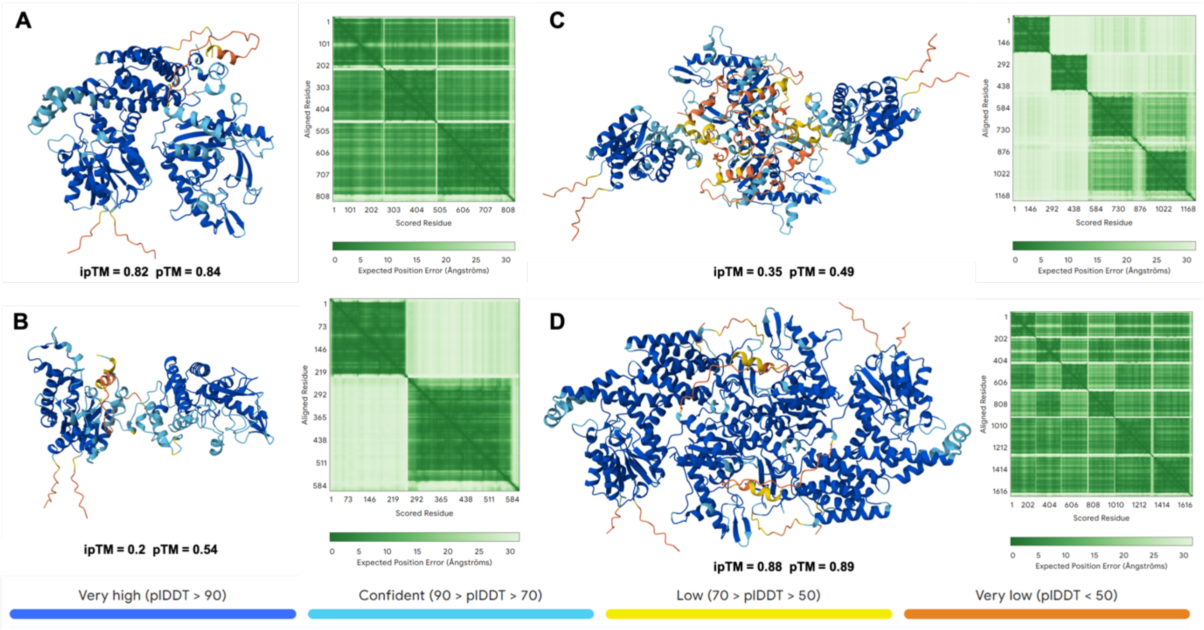

Figure S1.23. Dimeric, trimeric and higher order complexes structure predicted by AlphaFold3 and assessment of model confidence and structural accuracy. Quality assessment for predicted structures of PipDFG trimeric complexes (**A**), PipFG dimeric complexes (**B**), double copy dimeric complexes structure of PipFG (**C**) and double copy trimeric complexes structure of PipDFG (**D**). (Left panels for each sub-graph) per-residue confidence scores (pLDDT) predicted by AlphaFold3, plotted across the sequence. Higher pLDDT values (>90) indicate regions of high structural reliability, while lower scores (<70) suggest flexible or uncertain regions. (Bottom of right panels for each sub-graph) pTM and ipTM scores predicted by AlphaFold3, A pTM score above 0.5 means the overall predicted fold for the complex might be similar to the true structure. ipTM measures the accuracy of the predicted relative positions of the subunits within the complex. Values higher than 0.8 represent confident high-quality predictions, while values below 0.6 suggest likely a failed prediction. ipTM values between 0.6 and 0.8 are a gray zone where predictions could be correct or incorrect.

References

[1] E. Bode, A. K. Heinrich, M. Hirschmann, D. Abebew, Y. Shi, T. D. Vo, F. Wesche, Y. Shi, P. Grün, S. Simonyi, N. Keller, Y. Engel, S. Wenski, R. Bennet, S. Beyer, I. Bischoff, A. Buaya, S. Brandt, I. Cakmak, H. Çimen, S. Eckstein, D. Frank, R. Fürst, M. Gand, G. Geisslinger, S. Hazir, M. Henke, R. Heermann, V. Lecaudey, W. Schäfer, S. Schiffmann, A. Schüffler, R. Schwenk, M. Skaljac, E. Thines, M. Thines, T. Ulshöfer, A. Vilcinskas, T. A. Wichelhaus, H. B. Bode, *Angew. Chem. Int. Ed.* 2019, *58*, 18957–18963.

[2] E. Bode, D. Assmann, P. Happel, E. Meyer, K. Münch, N. Rössel, H. B. Bode, *Bio-Protoc.* 2023, *13*, e4709.

[3] S. Thoma, M. Schobert, *FEMS Microbiol. Lett.* 2009, *294*, 127–132.

[4] L. R. Hmelo, B. R. Borlee, H. Almblad, M. E. Love, T. E. Randall, B. S. Tseng, C. Lin, Y. Irie, K. M. Storek, J. J. Yang, R. J. Siehnel, P. L. Howell, P. K. Singh, T. Tolker-Nielsen, M. R. Parsek, H. P. Schweizer, J. J. Harrison, *Nat. Protoc.* 2015, *10*, 1820–1841.

[5] R. A. McClure, A. W. Goering, K.-S. Ju, J. A. Baccile, F. C. Schroeder, W. W. Metcalf, R. J. Thomson, Neil. L. Kelleher, *ACS Chem. Biol.* 2016, *11*, 3452–3460.

[6] R. Silva-Rocha, E. Martínez-García, B. Calles, M. Chavarría, A. Arce-Rodríguez, A. de las Heras, A. D. Páez-Espino, G. Durante-Rodríguez, J. Kim, P. I. Nikel, R. Platero, V. de Lorenzo, *Nucleic Acids Res.* 2012, *41*, D666–D675.

[7] N. Vodovar, M. Vinals, P. Liehl, A. Basset, J. Degrouard, P. Spellman, F. Boccard, B. Lemaitre, *Proc. Natl. Acad. Sci.* 2005, *102*, 11414–11419.

[8] T. Durfee, R. Nelson, S. Baldwin, G. Plunkett, V. Burland, B. Mau, J. F. Petrosino, X. Qin, D. M. Muzny, M. Ayele, R. A. Gibbs, B. Csörgő, G. Pósfai, G. M. Weinstock, F. R. Blattner, *J. Bacteriol.* 2008, *190*, 2597–2606.

[9] O. Schimming, F. Fleischhacker, F. I. Nollmann, H. B. Bode, *ChemBioChem* 2014, *15*, 1290–1294.

[10] E. Bode, A. O. Brachmann, C. Kegler, R. Simsek, C. Dauth, Q. Zhou, M. Kaiser, P. Klemmt, H. B. Bode, *ChemBioChem* 2015, *16*, 1115–1119.

[11] N. Neubacher, N. J. Tobias, M. Huber, X. Cai, T. Glatter, S. J. Pidot, T. P. Stinear, A. L. Lütticke, K. Papenfort, H. B. Bode, *Nat. Microbiol.* 2020, *5*, 1481–1489.

[12] K. A. J. Bozhueyuek, J. Watzel, N. Abbood, H. B. Bode, *Angew. Chem. Int. Ed.* 2021, *60*, 17531–17538.
